# Extent of genome-wide linkage disequilibrium in Australian Holstein-Friesian cattle based on a high-density SNP panel

**DOI:** 10.1186/1471-2164-9-187

**Published:** 2008-04-24

**Authors:** Mehar S Khatkar, Frank W Nicholas, Andrew R Collins, Kyall R Zenger, Julie AL Cavanagh, Wes Barris, Robert D Schnabel, Jeremy F Taylor, Herman W Raadsma

**Affiliations:** 1 Centre for Advanced Technologies in Animal Genetics and Reproduction (ReproGen), University of Sydney, Camden, NSW 2570, Australia; 2 Human Genetics Division, University of Southampton, Southampton General Hospital, Southampton, SO16 6YD, UK; 3 CSIRO Livestock Industries, St Lucia, QLD 4067, Australia; 4 CRC for Innovative Dairy Products, Level 1, 84 William Street, Melbourne, Vic, 3000, Australia; 5 Division of Animal Sciences, University of Missouri, Columbia, MO 65211, USA

## Abstract

**Background:**

The extent of linkage disequilibrium (LD) within a population determines the number of markers that will be required for successful association mapping and marker-assisted selection. Most studies on LD in cattle reported to date are based on microsatellite markers or small numbers of single nucleotide polymorphisms (SNPs) covering one or only a few chromosomes. This is the first comprehensive study on the extent of LD in cattle by analyzing data on 1,546 Holstein-Friesian bulls genotyped for 15,036 SNP markers covering all regions of all autosomes. Furthermore, most studies in cattle have used relatively small sample sizes and, consequently, may have had biased estimates of measures commonly used to describe LD. We examine minimum sample sizes required to estimate LD without bias and loss in accuracy. Finally, relatively little information is available on comparative LD structures including other mammalian species such as human and mouse, and we compare LD structure in cattle with public-domain data from both human and mouse.

**Results:**

We computed three LD estimates, *D*', *Dvol *and *r*^2^, for 1,566,890 syntenic SNP pairs and a sample of 365,400 non-syntenic pairs. Mean *D*' is 0.189 among syntenic SNPs, and 0.105 among non-syntenic SNPs; mean *r*^2 ^is 0.024 among syntenic SNPs and 0.0032 among non-syntenic SNPs. All three measures of LD for syntenic pairs decline with distance; the decline is much steeper for *r*^2 ^than for *D*' and *Dvol*. The value of *D*' and *Dvol *are quite similar. Significant LD in cattle extends to 40 kb (when estimated as *r*^2^) and 8.2 Mb (when estimated as *D*'). The mean values for LD at large physical distances are close to those for non-syntenic SNPs. Minor allelic frequency threshold affects the distribution and extent of LD. For unbiased and accurate estimates of LD across marker intervals spanning < 1 kb to > 50 Mb, minimum sample sizes of 400 (for *D*') and 75 (for *r*^2^) are required. The bias due to small samples sizes increases with inter-marker interval. LD in cattle is much less extensive than in a mouse population created from crossing inbred lines, and more extensive than in humans.

**Conclusion:**

For association mapping in Holstein-Friesian cattle, for a given design, at least one SNP is required for each 40 kb, giving a total requirement of at least 75,000 SNPs for a low power whole-genome scan (median *r*^2 ^> 0.19) and up to 300,000 markers at 10 kb intervals for a high power genome scan (median *r*^2 ^> 0.62). For estimation of LD by *D' *and *Dvol *with sufficient precision, a sample size of at least 400 is required, whereas for *r*^2 ^a minimum sample of 75 is adequate.

## Background

Recent developments in high-throughput genotyping technology [1-3] and the discovery of large numbers of SNPs through sequencing of the cattle genome [4,5] have generated enthusiasm and interest in genome-wide association mapping and marker-assisted selection in dairy cattle. Mapping by association requires a population-based sample rather than specific families. Association studies rely on the fact that alleles at loci that surround a quantitative trait nucleotide (QTN) tend to co-segregate. If the marker is sufficiently close to the QTN, the association remains intact in the majority of the individuals in the population, even after many generations. This non-random association between alleles at different loci is called linkage disequilibrium (LD). Such allelic associations are mostly due to physical proximity but are also affected by population history and evolutionary forces [6]. The main limitation of association analyses is the requirement for sufficient markers to provide a high chance of identifying markers in LD with all important QTN. The marker density required for a successful association analysis, and for subsequent marker assisted selection (MAS), depends on the extent of LD across the genome.

Several measures of LD have been devised [7-12], and two measures, *D' *and *r*^2^, each with different statistical properties [13], are commonly used. Both range from 0 (no disequilibrium) to 1 (complete disequilibrium), but their interpretation is different. For biallelic markers, *D' *is equal to 1 if one or more of the four possible haplotypes is absent, and is < 1 if all four possible haplotypes are present. Most studies in livestock have reported the extent of LD based on *D'*. *D' *is most useful for representing historical recombination patterns, which are central to determining the extent and pattern of LD over a genome. *D' *is especially helpful in understanding long-range LD. Recently Chen et al. [14] suggested a volume measure of LD *Dvol *equivalent to *D' *and reported that this measure is more robust when estimated from small samples.

The measure *r *represents the statistical correlation between two sites, and takes the value of 1 (perfect LD) for a pair of biallelic markers if only two haplotypes are present within a population. Hence in order for *r*^2 ^to be 1, allelic frequencies of the two SNPs in question need to be identical in addition to both being in LD. The power of association mapping is inversely proportional to *r*^2^, and to achieve the same power by typing a SNP in LD with a QTN versus typing the QTN directly, the sample size must be increased by a factor of 1/*r*^2 ^[13]. However, if more than one SNP is used to predict the effect of a QTN, then the association between the QTN and haplotypes is more informative.

The average extent of LD in the human genome has been extensively studied: it extends up to 50 kb but is highly variable, depending on the population and threshold used to measure LD [13,6,15,16]. Studies on LD in cattle [17-22], sheep [23], pig [24,25], horse [26], dog [27,28] and chicken [29] have shown that LD in livestock populations is much more extensive than in humans. This is mainly due to smaller effective population size and, in some circumstances, stronger selection that has recently occurred in livestock populations [23].

The estimates of LD reported in cattle are mostly based on microsatellite markers. LD in humans estimated from SNP markers has been reported to be smaller than estimates from microsatellites [13]. There is limited information available on the extent of LD between SNP markers in cattle and it is mainly limited to regions used in fine mapping [30,31] or for a single chromosome [32,22]. Furthermore, studies in cattle have usually involved relatively small sample sizes which are both subject to bias and loss of accuracy when estimating *D' *and *r*^2^; and such bias may vary with inter-marker distance. Finally, effects of minor allele frequency (MAF) of SNP loci on estimates of LD have not been reported in cattle.

In an ongoing gene discovery program in dairy cattle, a panel of 1546 Holstein-Friesian bulls was genotyped for 15,036 SNPs using a high-throughput genotyping service [34]. This data set was recently described and used for the construction of a bovine HapMap [33]. The present paper presents additional results of LD analyses of this genotype data. This study explores the effect of MAF and sample size on LD parameters and suggests the minimum sample size required for useful estimates. We also compare a recently suggested volume measure of LD [14] to the more commonly used statistics. Public-domain SNP data from mice and humans are also analysed to compare LD from different mammalian species using the same statistics and methods.

## Results

Of a total of 15,036 SNPs genotyped in this study, 9,195 survived filtering on MAF (> = 0.05) and HWE (P > 0.0001). The distribution of SNPs on different chromosomes is summarised in Table 1, ranging from 528 on BTA1 to 158 on BTA27. The overall mean MAF was 0.286 ± 0.0013. The distribution of MAF over the genome is approximately uniform (see Additional file 1, Figure S1), consistent with the ascertainment of SNP discovery identifying primarily the common SNPs within the bovine genome. The genomic coordinates of each SNP, together with its MAF, are available in Additional file 2.

**Table 1:** Number of SNPs, mean minor allelic frequency (MAF), spacing between adjacent SNPs and estimated of LD on different autosomes in present study.

**BTA**	**No. Snp**	**Mean MAF**	**Spacing (kb)**	**Mean *D'***	**Median *D'***	**Mean adj *D'***	**Median adj *D'***	**Swept radius based on *D' ***	**Mean *r*^2^**	**Median *r*^2^**	**Mean adj *r*^2^**	**Median adj *r*^2^**
1	528	0.27	276	0.18	0.11	0.70	0.88	11993 (11514 – 12513)	0.013	0.003	0.337	0.107
2	462	0.30	270	0.18	0.11	0.70	0.87	8968 (8607 – 9360)	0.013	0.003	0.323	0.101
3	469	0.28	248	0.18	0.11	0.70	0.89	9307 (8996 – 9640)	0.014	0.003	0.309	0.096
4	384	0.29	284	0.18	0.12	0.66	0.81	9825 (9354 – 10346)	0.014	0.003	0.317	0.090
5	417	0.28	279	0.18	0.11	0.70	0.89	8945 (8623 – 9291)	0.014	0.003	0.316	0.109
6	443	0.28	252	0.18	0.11	0.70	0.92	10311 (9878 – 10784)	0.013	0.003	0.328	0.099
7	357	0.30	282	0.17	0.11	0.70	0.84	8883 (8564 – 9227)	0.015	0.003	0.303	0.118
8	389	0.29	265	0.19	0.12	0.68	0.89	15359 (14440 – 16403)	0.015	0.003	0.293	0.106
9	265	0.29	357	0.20	0.13	0.67	0.84	9109 (8579 – 9708)	0.018	0.004	0.330	0.086
10	403	0.29	238	0.19	0.12	0.68	0.85	7725 (7455 – 8016)	0.016	0.004	0.311	0.107
11	452	0.28	224	0.18	0.11	0.70	0.88	10830 (10453 – 11235)	0.013	0.003	0.312	0.099
12	276	0.28	278	0.19	0.12	0.69	0.90	7271 (6902 – 7683)	0.015	0.003	0.325	0.120
13	406	0.28	203	0.19	0.12	0.73	0.97	8919 (8633 – 9225)	0.017	0.003	0.382	0.165
14	303	0.30	271	0.20	0.13	0.72	0.94	7309 (7002 – 7643)	0.019	0.004	0.349	0.146
15	309	0.27	242	0.21	0.13	0.67	0.89	7530 (7156 – 7945)	0.017	0.004	0.336	0.100
16	317	0.30	230	0.19	0.13	0.71	0.91	5861 (5639 – 6102)	0.019	0.004	0.368	0.168
17	302	0.28	231	0.20	0.12	0.72	0.94	7788 (7503 – 8094)	0.018	0.004	0.319	0.126
18	294	0.30	212	0.19	0.12	0.66	0.86	7342 (7009 – 7708)	0.015	0.003	0.312	0.092
19	344	0.29	183	0.19	0.12	0.72	0.92	7500 (7240 – 7778)	0.016	0.003	0.290	0.118
20	254	0.27	265	0.21	0.14	0.68	0.82	10315 (9718 – 10990)	0.021	0.005	0.345	0.150
21	181	0.29	345	0.21	0.13	0.61	0.68	5273 (4923 – 5676)	0.019	0.004	0.234	0.064
22	252	0.29	230	0.21	0.14	0.72	0.91	7681 (7358 – 8033)	0.022	0.005	0.329	0.114
23	260	0.29	183	0.25	0.16	0.73	0.94	8610 (8197 – 9065)	0.024	0.006	0.318	0.117
24	222	0.29	271	0.21	0.14	0.65	0.80	5437 (5140 – 5770)	0.019	0.005	0.331	0.083
25	225	0.30	187	0.19	0.12	0.66	0.84	4417 (4187 – 4673)	0.019	0.004	0.308	0.097
26	184	0.27	255	0.24	0.17	0.71	0.90	7191 (6672 – 7797)	0.022	0.006	0.289	0.092
27	158	0.32	271	0.19	0.13	0.65	0.78	-	0.021	0.005	0.325	0.099
28	159	0.29	249	0.22	0.14	0.66	0.79	5916 (5474 – 6435)	0.020	0.005	0.288	0.068
29	180	0.30	250	0.21	0.13	0.67	0.85	4805 (4534 – 5110)	0.024	0.004	0.313	0.095
All	9195	0.29	252	0.19	0.12	0.69	0.88	8154 (8085 – 8225)	0.016	0.003	0.321	0.110

The values in the parentheses of swept radius are confidence interval of swept radius.

adj means estimate between adjacent SNPs only.

### Linkage disequilibrium among syntenic SNP pairs

**Figure 1:**
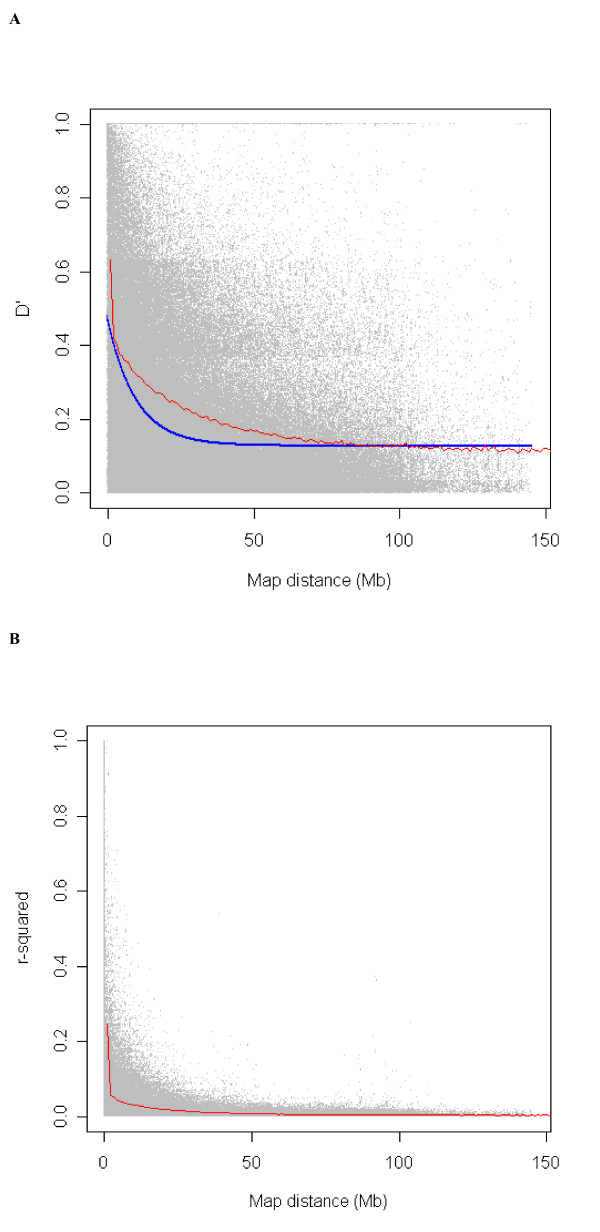
**Distribution of LD between SNP pairs in relation to the physical distance between loci (Mb), pooled over all autosomes.** The red line shows average LD in each 500 kb sliding window. Grey dots are individual LD estimates plotted again inter-marker distances. Figure 1A shows *D' *and Figure 1B shows *r*^2^. The blue line in Figure 1A shows the theoretical distribution from the Malécot model.

Two of the most commonly used measures of LD, *D' *and *r*^2^, were estimated for each pair-wise combination of SNPs on each chromosome (syntenic SNPs): a total of 1,566,890 pairs were analyzed for all autosomes. The mean values of *D' *and *r*^2 ^for individual autosomes are presented in Table 1. The mean *D' *and *r*^2^, pooled over autosomes (1–29) in different categories of map distances, are summarized in Table 2. Similar tables for individual chromosomes are provided in Additional file 3. The distribution of *D' *and *r*^2 ^with respect to the physical distance separating loci is presented in Figures 1A and 1B, respectively. As expected, there is a gradual decline in *D' *with increasing physical distance between SNPs: for SNPs up to 1 kb apart, the mean *D' *is 0.99; for SNPs separated by 200–500 kb the mean *D' *is 0.46, and for SNPs separated by more than 50 Mb, the mean *D' *is 0.11. The distribution of expected *D' *obtained from fitting the Malécot model [34,15] is also shown in Figure 1A. From this distribution, the estimated swept radius (the distance over which LD declines to ~37% of its initial value) is 8.2 Mb.

**Table 2:** Mean linkage disequilibrium among syntenic SNPs over different map distances, pooled over all autosomes.

Distance	N	Mean *D'*	SD *(D')*	Median *D'*	Mean *r*^2^	SD (*r*^2^)	Median *r*^2^	25th percentile *r*^2^	Min no SNP required to cover genome
0–1 kb	3856	0.99	0.09	1.00	0.767	0.335	0.989	0.496	3,000,000
1–10 kb	1475	0.95	0.16	1.00	0.592	0.374	0.619	0.205	300,000
10–20 kb	546	0.87	0.24	1.00	0.402	0.352	0.280	0.082	150,000
20–40 kb	1089	0.80	0.28	0.97	0.309	0.311	0.186	0.057	75,000
40–60 kb	829	0.66	0.33	0.71	0.196	0.235	0.114	0.027	50,000
60–100 kb	1529	0.61	0.34	0.64	0.153	0.210	0.066	0.015	30,000
100–200 kb	3899	0.54	0.33	0.52	0.108	0.156	0.047	0.013	15,000
200–500 kb	11202	0.46	0.31	0.41	0.073	0.106	0.034	0.009	6,000
0.5–1 Mb	18082	0.41	0.29	0.36	0.057	0.083	0.026	0.006	3,000
1–2 Mb	36272	0.38	0.28	0.33	0.049	0.076	0.022	0.005	1,500
2–5 Mb	103940	0.34	0.26	0.27	0.036	0.056	0.016	0.004	750
5–10 Mb	163439	0.27	0.23	0.21	0.024	0.038	0.010	0.002	300
10–20 Mb	285318	0.21	0.19	0.15	0.013	0.022	0.006	0.001	150
20–50	575734	0.14	0.14	0.10	0.006	0.010	0.002	0.001	60
> 50 Mb	359680	0.11	0.12	0.08	0.003	0.007	0.001	0.000	NA
Non-syntenic	365400	0.11	0.11	0.07	0.003	0.005	0.002	0.000	

Compared to *D'*, there is (as expected) a steeper decline in *r*^2 ^with increasing distance between syntenic SNPs (Table 2, Figure 1B). The mean *r*^2 ^across autosomes is 0.016 ± .00005. A mean *r*^2 ^above 0.3 was observed only for SNPs less than 40 kb apart, and *r*^2 ^declines rapidly with increasing distances. As can be seen in Table 2, starting from a maximum of 0.77 for SNPs less than 1 kb apart,*r*^2 ^reduces to less than 10% of this value for SNPs greater than 500 kb apart. The value of *r*^2 ^at a distance of more than 50 Mb (0.0034) is close to average *r*^2 ^among the non-syntenic pairs (0.0032).

**Figure 2:**
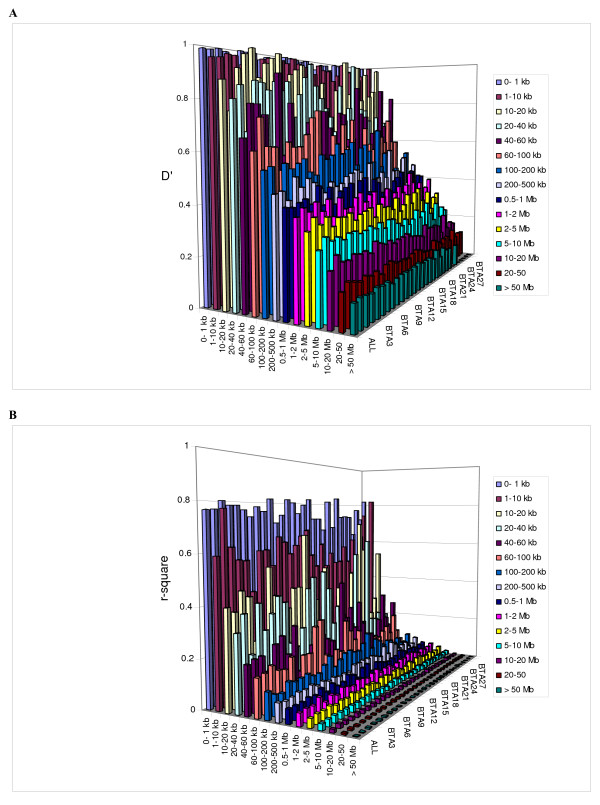
Comparison of mean LD estimates shown as height of the bar in different distance bins of inter-marker spacing on different chromosomes (BTA1-29) and combined genome-wide (ALL) where Figure 2A shows *D' *and Figure 2B shows *r*^2^.

Similar trends for *D' *and *r*^2 ^are evident for all the individual autosomes, although there is variation in the trend of decline (see Additional files 3, 4 and 5 for individual chromosomes). Heat maps for *D' *and *r*^2 ^for individual chromosomes (see Additional files 6 and 7) clearly display variation in the pattern of LD across the genome. Figure 2A and Figure 2B compare the average LD in different distance bins of individual chromosomes with combined over genome. There is more variation in *r*^2 ^across chromosomes at closer distance bins. The variation in LD across chromosomes suggests that inference of genome-wide LD based on single or few chromosomes can be biased. A different analysis of this variation in LD has recently been presented [33]. The swept radius for *D' *of individual chromosomes shows that LD is higher for the longer chromosomes than the shorter chromosomes (ρ = 0.7). This can be explained by fewer recombinations per unit of physical distance in long chromosomes than in short chromosomes, as observed in the human genome.

A more robust measure of LD termed the volume measure of *D' *(*Dvol*) has recently been proposed [35]. We computed *Dvol *for all the syntenic pairs. Since the value of *D' *and *Dvol *were quite similar (ρ = 0.99) in this data, the estimates of *Dvol *are not presented here, can be obtained from corresponding author.

### LD among non-syntenic SNP pairs

**Figure 3:**
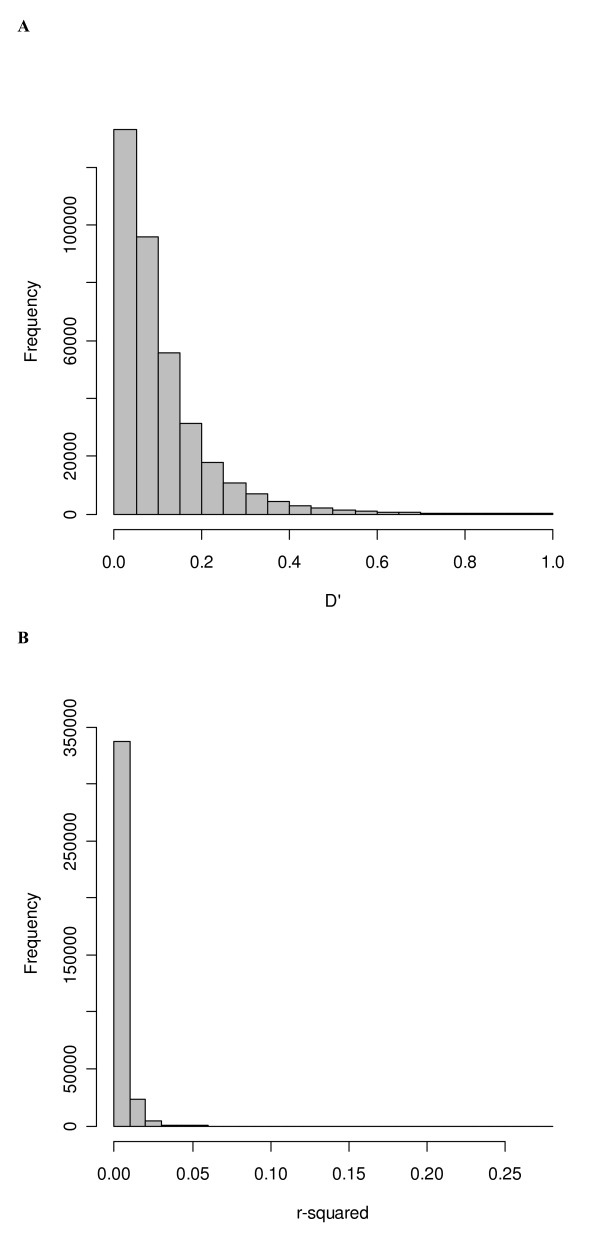
Frequency distribution of LD estimates between non-syntenic pairs of SNPs where Figure 3A shows *D' *and Figure 3B shows *r*^2^.

The LD estimates for a sample of 365,400 non-syntenic pairs (two SNPs present on different chromosome) were computed. The mean *D' *for non-syntenic SNPs is 0.105 ± 0.0002 (Table 2). The distribution of *D' *for non-syntenic SNP pairs presented in Figure 3A indicates a larger proportion of non-syntenic pairs have small values of *D'*. The mean value *r*^2 ^for non-syntenic SNPs is 0.0032 ± 0.000008. The distribution of *r*^2 ^for non-syntenic pairs is shown in Figure 3B. The comparison of *D' *and *Dvol *estimated for non-syntenic SNP pairs shows that both estimates are quite similar (ρ = 0.99) for this sample (see Additional files 1, Figure S2).

### The relationship between LD and average minor allelic frequency for non-syntenic SNPs

The estimate of *D' *seems to inflate with lower average minor allelic frequency (MAF) for non-syntenic SNP pairs (see Additional files 1, Figure S3). There are 1.27 percent of non-syntenic SNP pairs with *D' *values above 0.5. Most of these *D' *values were observed for SNP pairs with low average MAF. However, there is no relationship between MAF and LD expressed as *r*^2 ^(see Additional files 1, Figure S4). It should be noted that all *r*^2 ^values for non-syntenic pairs are very small. The maximum value for *r*^2 ^observed for non-syntenic pairs is only 0.27, and only 9 pairs (0.002 %) have an *r*^2 ^value of more than 0.1. Similarly, we also analysed *chi-squared *for non-syntenic SNP pairs and only small values of *chi-squared *were observed for the non-syntenic SNP pairs (data not shown). The proportion of non-syntenic pairs with a high *D' *value is small (1.27 %). However, of the pairs with a mean MAF less than 0.1, 18.9 percent have *D' *above 0.5 and there are no pairs with *r*^2 ^> 0.1. These results suggest that *D' *is comparatively more sensitive to changes in MAF due to the increased likelihood of a missing haplotype when MAF is low. In contrast, *r*^2 ^appears to be largely unaffected by low MAF. The relationship between *Dvol *and mean MAF shows a similar trend to that for *D' *(see Additional files 1, Figure S5), indicating that *Dvol *is also inflated for loci with rare alleles.

### Effect of MAF on the extent of syntenic LD

**Figure 4:**
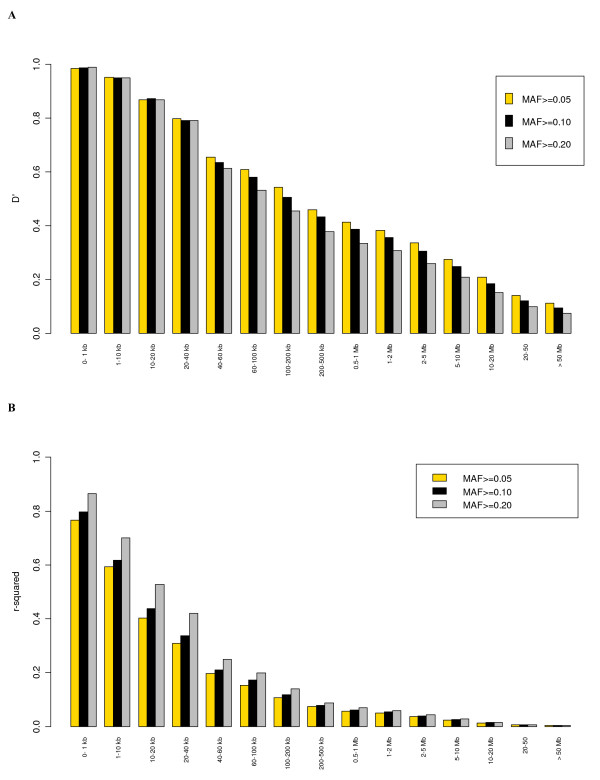
Mean LD estimates at different physical distances pooled over all the autosomes and estimated at three minimum threshold levels of cutoff for MAF where Figure 4A shows *D' *and Figure 4B shows *r*^2^.

The effect of MAF on the extent of LD amongst syntenic SNPs was studied using three different minimum MAF thresholds of 0.05, 0.1 and 0.2 (Figure 4A, B). Minimum MAF has a strong effect on mean *r*^2 ^especially at short distances (up to 40 kb) (Figure 4B). For example, for SNPs within 1–10 kb, the mean *r*^2 ^was 0.59 for MAF ≥ 0.05 but was as high as 0.70 for loci with MAF ≥ 0.2. A likely explanation is that as the MAF threshold increases, there is an increase in the number of SNP pairs with similar allele frequencies, and, consequently, an increase in *r*^2^. Compared to *r*^2^, there is an opposite effect of MAF threshold on *D' *(Figure 4A). There is almost no effect at close distances, especially up to 40 kb. However, at larger distances, higher MAF thresholds are associated with lower values of mean *D'*, which is consistent with earlier studies [23].

### Effect of sample size on accuracy and bias of LD estimators

**Figure 5:**
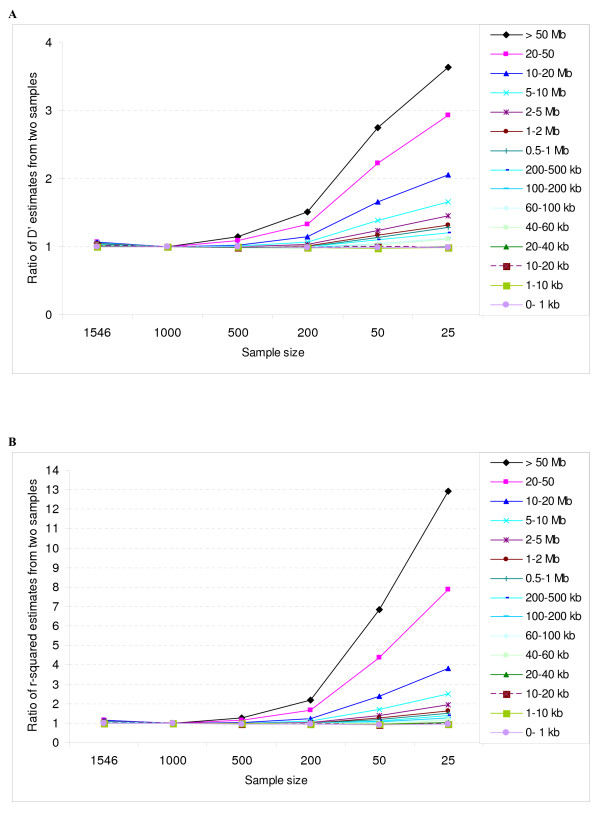
**Bias in the mean LD estimates at different physical distances pooled over all autosomes estimated using different sample sizes shown as proportion of the estimates obtained from a sample to the reference sample of 1000 animals where Figure 5A shows *D' *and Figure 5B shows *r*^2^.** The bias is expressed as a ratio of sample estimates to the reference estimate.

**Figure 6:**
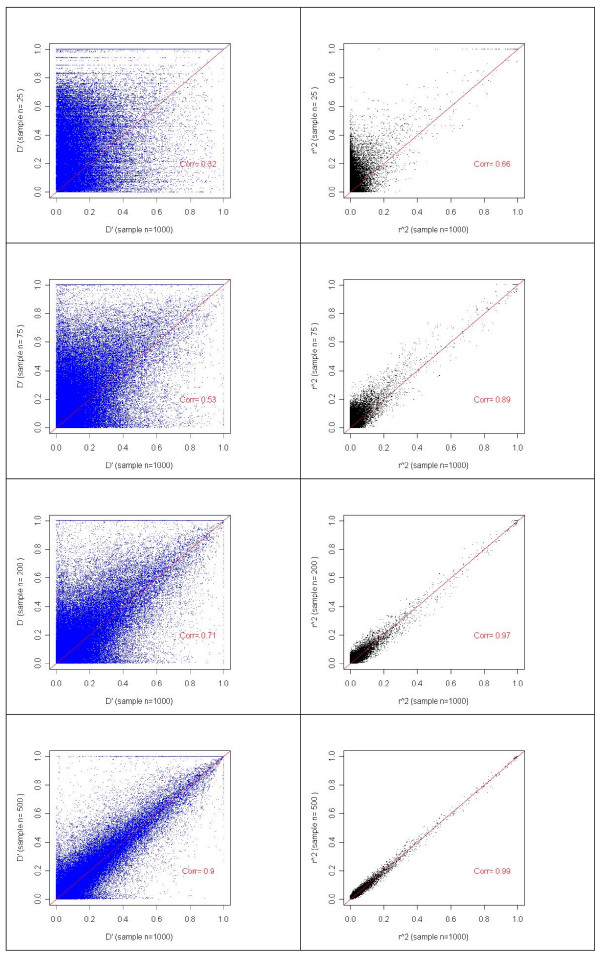
**Relationship between the pair-wise estimate of LD estimated from four sample sizes (n = 25, 75, 200 and 500) against a reference sample size of n = 1000 for one chromosome as an example.** Figure shows individual estimate as dot plots, correlation coefficient and a hypothetical line for reference where X = Y (i.e. not fitted).

Table 3, Figures 5A, B and 6 show the effect of sample size on accuracy and bias in estimation of the extent of LD (also see Additional files 1, Figure S6 and S7). In all cases of small samples sizes, estimates were biased upwards, and this trend was more noticeable for LD measured across marker intervals greater than 40 kb, and became exponentially worse for long-range LD estimated from inter-marker distances of > 10 Mb. Both estimates of LD based on the full sample of 1546 bulls were only slightly higher (%) as compared to estimates based on the reference sample of 1000 bulls. This may be because of higher average kinship between the 1546 bulls as compared with the subset of 1000 bulls. In Figures 5A and 5B, bias is expressed as the ratio of the LD estimate from the test sample of size n, to the LD estimate from the reference sample (n = 1000).

**Table 3:** Correlation between the pair-wise estimates of LD obtained from different sample sizes against a reference sample of 1000 animals.

		*Correlation between the estimates from two different samples*		
Sample 1	Sample 2	*D'*	*r*^2^	MAF

1000	25	0.33 (0.27–0.39)	0.70 (0.57–0.77)	0.90 (0.82–0.94)
1000	50	0.48 (0.40–0.58)	0.85 (0.79–0.92)	0.96 (0.94–0.97)
1000	75	0.56 (0.51–0.64)	0.91 (0.88–0.95)	0.97 (0.94–0.98)
1000	100	0.63 (0.55–0.73)	0.94 (0.91–0.96)	0.98 (0.97–0.99)
1000	150	0.72 (0.66–0.77)	0.96 (0.94–0.98)	0.98 (0.97–0.99)
1000	200	0.77 (0.71–0.83)	0.98 (0.96–0.99)	0.99 (0.98–0.99)
1000	300	0.85 (0.80–0.89)	0.99 (0.98–0.99)	0.99 (0.99–1.00)
1000	400	0.89 (0.86–0.92)	0.99 (0.99–1.00)	1.00 (0.99–1.00)
1000	500	0.93 (0.90–0.95)	0.99 (0.99–1.00)	1.00 (1.00–1.00)
1000	1546	0.95 (0.93–0.96)	0.99 (0.99–1.00)	1.00 (1.00–1.00)

The values in the parentheses are the range of correlation coefficient obtained from analysis of 29 individual autosomes.

Figure 6 and Table 3 show the relative accuracy of LD estimates from different sample sizes, expressed as correlations between the LD parameters obtained from the test sample and the LD estimates obtained from the reference sample of 1000, for all chromosomes combined. For samples sizes of 75 or less, the accuracy of *r*^2 ^appears to be significantly compromised. At samples sizes of 200, both the regression, correlation and residual variance of prediction shows goodness of fit consistent with estimates of the full sample of 1000. For *D' *a substantially larger sample size of 400 or more is required to obtain accurate estimates, compared with the estimates obtained for 1000 animals (Table 3). The accuracy of prediction, as indicated by correlations, is shown in Table 3. We compared the volume measure of *D' *(*Dvol*) [14] with *D' *estimated from different sample sizes for two chromosomes. As mentioned earlier, the estimates of *D' *and *Dvol *are quite similar especially for large samples. The correlation of *Dvol *estimated from different sample sizes was similar to that for *D' *(see Additional files 8, Table S2) indicating that accurate estimation of *Dvol *requires a sample size comparable to *D'*. Overall, we can conclude that a sample size of at least 75 is required for a reasonable estimate of *r*^2^, and a sample of at least 400 is required for *D' *and *Dvol*. In contrast to LD, MAF could be accurately estimated from samples sizes as small as 50 (Table 3). However, this comparison for MAF is based on the common SNPs with MAF more than 0.05 used in this study. Additional comparison of 1446 SNPs with less than 0.05 MAF (not included in the LD analysis) indicated that only large samples were able to detect the polymorphism in the majority of the SNPs (data not shown).

### Comparison of LD in the cattle, mouse and human genomes

**Figure 7:**
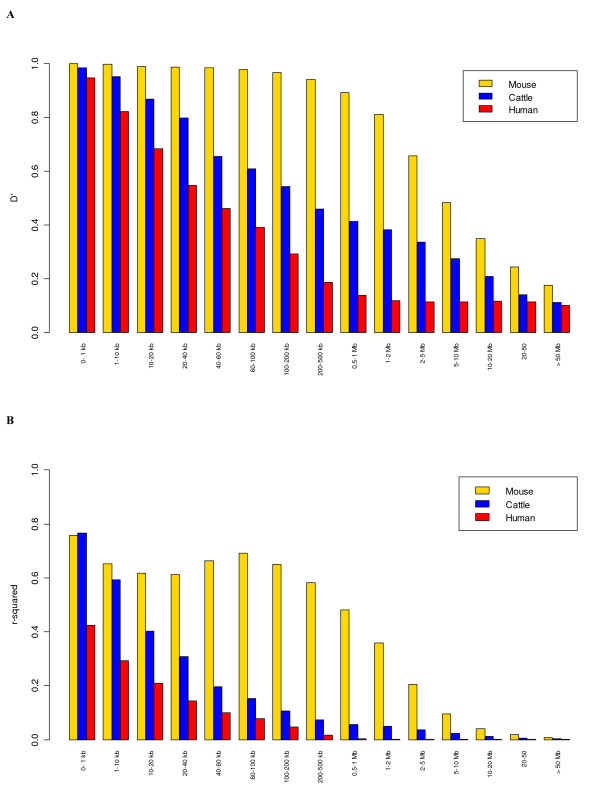
Mean LD estimates at different physical distances in cattle, mouse and human where Figure 7A shows *D' *and Figure 7B shows *r*^2^.

In order to compare the extent of LD in the bovine genome with mouse and human, we analysed some mouse and human SNP genotyping data available in the public domain. Although the extent of LD in cattle is much greater than in human (Figure 7A, B), the pattern of decay in LD with distance is similar. In contrast, LD in a mouse population derived from the intercrossing of 8 inbred lines, maintained for 50 generations by pseudorandom matting [35], shows extensive extended LD for both *D' *and *r*^2^. The extent of LD in this population extends up to 10 Mb for *D'*, and for *r*^2 ^useful LD of 0.3 extended more than 2 Mb. In the human data set, the distance to *r*^2 ^= 0.3 extends only to 10 kb.

## Discussion

This is a comprehensive study of LD with a high density SNP panel in HF dairy cattle and currently reflects the best estimates of genome-wide LD in this breed based on number of animals screened and number of SNPs genotyped. The pairwise measures of LD decline over increasing distance between SNPs. The LD estimated by *D' *appears to be quite extensive and is much higher in cattle than in humans. This may be due to random drift caused by relatively small effective population in dairy cattle [36]. Comparable estimates of extensive LD based on *D' *estimated from microsatellites have been reported in cattle [17-21], sheep [23], pig [24], horse [26], dog [27] and chicken [29]. The LD between SNP markers reported in the present study is slightly smaller than that estimated between microsatellite markers in earlier studies [17,21] and this may be explained in part by the differences in the mutation rate between these two types of markers and reflect the more recent origin of microsatellite polymorphisms. Secondly this may be due to the higher power for detecting LD when using markers with many alleles (e.g., microsatellites) as compared to biallelic SNP markers [37]. The difference in LD detected using SNP and microsatellite loci was more pronounced in humans [13].

The *D' *metric has been suggested as a good measure to explain the extent of LD in population and variation of LD over the genome [22,15]. However, individual values of *D' *are more influenced by variation in allele frequencies than for the *r*^2 ^metric. This is reflected in the inflated *D' *values at low MAF [6]. For non-syntenic SNP pairs we observed higher values of *D' *for the pairs with low mean MAF. It is known that SNPs of different MAFs have different LD properties [13]. Higher *D' *between loci with rare alleles is mainly due to population genetic effects (rare alleles are, in general, younger than common alleles, and hence may still be in LD) and to effects of sampling. Smaller samples may fail to sample rare fourth gametes and, therefore, can inflate the *D' *estimate [11]. On the other hand SNPs with rare alleles tend to have lower *r*^2 ^values. The inflated *D' *estimates between non-syntenic SNPs with rare alleles are probably due to the effect of sampling caused by random drift or the loss of rare haplotypes in sampling in the present study.

The decline in LD is much steeper for *r*^2 ^than for *D'*. Such differences in the pattern of *D' *and *r*^2 ^over physical distances have previously been observed in humans [38,39]. These two measures, which have been widely used in practice, have different statistical properties [13]: *D' *focuses on historical recombination, which is central to defining the extent and pattern of LD over a genome. However, *r*^2 ^is more useful for predicting the power of association mapping. To obtain the same power as obtained when testing the causative quantitative trait nucleotide (QTN)/mutation, the sample size required for association mapping is inversely proportional to *r*^2 ^[13,40,41]. From the pattern of decline of *r*^2 ^the average useful LD for single-point association mapping in this population extends only up to 40 kb which suggests that at least 75,000 SNPs are required for a whole genome association scan. At this distance *r*^2 ^values between adjacent SNPs decrease to an average of ~0.3 (median *r*^2 ^= 0.19). At this spacing a QTN would be at a maximum of 20 kb (located at the mid point of the interval) from an adjacent SNP which would give an average *r*^2 ^of 0.4 (median *r*^2 ^= 0.28) between SNP and QTN. However, if more stringent criteria are considered for higher power genome scans, then the number of SNPs required would be 150,158 (one SNP every 20 kb) and 300,631 (one SNP every 10 kb) to obtain average *r*^2 ^values between adjacent SNPs of 0.4 (median *r*^2 ^= 0.28), and 0.6 (median *r*^2 ^= 0.62), respectively. In addition there is a lot of variation in *r*^2^, as indicated by the large standard deviation of *r*^2 ^(Table 2), within an interval of extent of LD considered. The 25^th ^percentile of *r*^2 ^(Table 2) indicates that only 75 % of the pairs of SNPs in the 1–10 kb distance bin have *r*^2 ^of more than 0.2. Similar low values of the 25th percentile are noted for other distance bins in Table 2. The variation in *r*^2 ^in a distance bin is partly because of the variation in LD in different genomic regions. In addition *r*^2 ^is dependent upon the matching allelic frequencies and are known to have very low values between markers even at very short distances [39]. This variation has been ignored in most studies while suggesting the number of SNPs required for genome scans based on average *r*^2 ^. To accommodate this variation, more SNPs will need to be genotyped in each interval which will increase the estimate of the total number of SNPs required for association mapping. However, these partial correlations can be exploited using multi-markers haplotype analysis which provides more discriminatory power as compared to individual SNPs to detect putative causal mutation. Recently McKay et al. [42] suggested one SNP every 100 kb to obtain an average *r*^2 ^of 0.15-0.2 between adjacent SNPs. Gautier et al. [43] reported the LD analysis of 526 SNPs mostly located on one chromosome in 14 breeds and suggested a common panel of 300,000 SNPs (one SNP every 10 kb) for association mapping in different breeds similar to the requirement for a high power panel within-breed study as shown here.

The number of SNPs required for association mapping can be reduced by excluding some of the redundant SNPs, by optimally using the LD information present in the population. However, there are differences in the pattern of LD across the genome. This can be addressed more precisely by identifying tag SNPs based on haplotype block structure information, as was done in the human HapMap project [44]. An attempt to construct the haplotype block map of the bovine genome and the concomitant identification of tag SNPs from this dataset was presented by Khatkar et al. [33] but at present only covers a relatively small proportion of the bovine genome based on 15,036 markers. Genome-wide identification of tags SNPs for the whole genome would be possible from a saturated haplotype block map of the bovine genome. Until such maps become available, the extent of LD as expressed by *r*^2 ^can provide an interim guide for number and spacing of the SNPs over the genome.

The extent of LD within a genome can be affected by a number of factors. Our results confirm that MAF has direct effects on the estimation of extent of LD. The *r*^2 ^between common SNPs is higher especially for SNPs at close physical distances. On the other hand *D' *between SNPs with low MAF is higher at longer distances. Similarly, sample size also affects the estimation of LD. The results in this study clearly indicate that the estimate of *D' *is affected most by sample size. It seems that for reliable estimates of *D' *a sample of 400 or more is required. Similar observations were also made for *Dvol*. The requirement of sample size would be even higher in human because of the larger effective population size. Hence, it may be suggested that analyses utilizing *D' *matrix (like construction of LD maps and HapMap) should be based on a large sample size and preferably from within-breed group samples.

However, the identification of tag SNPs, which is generally based on *r*^2 ^values, can be accomplished using smaller samples. Similar estimates of correlations between the estimates of *r*^2 ^were obtained from different samples in a simulation study by Visscher [45]. Similarly, a small sample size of 50 and above can provide precise estimate of MAF for common SNPs in the population (Table 3).

## Conclusion

We have conducted an extensive LD analysis using 9,195 SNPs genotyped on 1546 Holstein Frisian bulls. Overall the extent of LD over different chromosomes was similar but varied in cattle. We compared three different measures of LD. All are affected by sample size although to different extents. We suggest that the sample size required to compute reliable estimates of *D' *or for any analysis (HapMap and LD maps) based on the *D' *matrix should be at least 400 samples. A similar sample size is required for *Dvol*. However, it seems that *r*^2 ^can be reliably estimated with smaller sample sizes of 75 individuals. Based on the extent of decline of *r*^2 ^we suggest more than 75,000 SNPs would be required for low power association mapping in the Holstein Friesian population and up to 300,000 SNPs for a high power study. The extent of LD in cattle is higher than in human, but much less than in a mouse population.

## Methods

### Cattle data

A total of 1,546 Holstein Friesian bulls were genotyped for 15,036 SNPs. A subset of 1,000 bulls (called the reference sample) was selected for LD analysis based on minimizing their pedigree relationship with each other. However, when studying the effect of sample size on LD estimators, estimates of LD from the entire sample of 1,546 bulls were also used. Of the total SNPs, 10,000 (MegAllele Genotyping Bovine 10,000 SNP Panel, ParAllele) were generated as part of the community project of the International Bovine Genome Sequencing Consortium (IBGSC) [46]. The remaining 5,036 custom SNPs were selected from the Interactive Bovine In Silico SNP (IBISS) database [47,48], from in-house sequencing, and from publications. A high-throughput SNP assay service provided by Affymetrix, Inc. was used for the genotyping. The locations of the SNPs were determined on the bovine sequence assembly Btau 3.1 [49] The details of the genotyping and mapping of SNPs were given in Khatkar et al. [33].

### Mouse Data

Data on 2202 mice genotyped for 13,459 SNPs were downloaded from the Wellcome Trust Centre for Human Genetics's web page [50,35] and analysed for *D' *and *r*^2 ^with Haploview [51] for all 19 autosomes. Pedigree structure was ignored for these computations and the results were pooled over all autosomes. This data set is not representative of natural populations: it resulted from the intercrossing of 8 inbred lines to create a population maintained for 50 generations of pseudo-random mating and referred to as heterogeneous stock mice. It is of interest, however, because of its central use in QTL mapping [35].

### Human Data

Data on 537 participants genotyped for 408,000 SNPs described by Fung et al. [52] were downloaded from NINDS Human Genetics DNA and Cell Line Repository [53]. A sample of 1500 SNPs on one chromosome (HSA15) was selected for estimation of LD parameters and comparison to mouse and cattle.

### Estimation of linkage disequilibrium

Estimates of the two standard descriptive linkage disequilibrium (LD) parameters, *D' *[7] and *r*^2 ^[54], were obtained via Haploview software [51] for all pair-wise combinations of SNPs on each chromosome. SNPs showing significant deviations from HWE (P < 0.0001) were excluded from analysis, as were SNPs with minor allelic frequency (MAF) < 0.05, and those genotyped in less than 50% of the bulls. The Malecot model for the relationship between LD and distance between loci [34]; [15] was fitted to the *D' *data on individual autosomes and combined over all autosomes. As shown in the results section, *D' *above 50 Mb were close to non-syntenic (background) LD, hence SNPs pairs with up to 50 Mb distance between them were included for fitting Malecot model. This also allowed similar window size for comparison of LD over the autosomes of different length. Fitting this model enabled estimation of the swept radius, the extent of LD that is useful for mapping [10] defined as the distance in kb over which LD declines to approximately 37% of its initial value. Estimates of LD statistics among a sample of non-syntenic SNP pairs were computed using the *genetics *package [55] in the R statistical software suite [56]. A random sample of 30 SNPs was chosen from each chromosome, and all non-syntenic pairs were used to assess the extent of non-syntenic LD.

We also computed a volume measure of *D' *(*Dvol*) as per the formulae given and implemented in the C codes of Chen et al. [14]. The program computes *Dvol *from the four haplotype counts. Four haplotype counts were computed with GOLD [57].

### Estimation of samples size on accuracy and bias of LD estimates

To examine the effect of sample size on the accuracy of estimation of LD parameters, samples of 25, 50, 75, 100, 150, 200, 300, 400, and 500 were randomly drawn from the total sample of 1,000 analysed in this study (Table 3, Figure 6). The effect of sample size on the accuracy and bias in estimation of the extent of LD obtained from the 1000 animal reference sample. The bias was expressed as a ratio of statistics from test sample size to the reference sample. The data consisting of all 1546 animals (which included some closely related animals) were also included for comparison here. The SNPs in each sample were evaluated for MAF and HWE. The LD between SNPs pairs for all autosomes was estimated for each sample, after excluding SNPs with MAF < 0.05 and HWE (P < 0.0001). Pair-wise estimates of LD and MAF were compared between different samples.

## Authors' contributions

MSK conceived the study, contributed in its design, data collection, analysis and was the primary author for assembling the manuscript. ARC participated in interpretation and manuscript preparation. FWN participated in data interpretation and manuscript preparation. KRZ contributed in generating SNP data and preliminary data analyses. JALC organized DNA sample preparation and participated in SNP discovery and SNP genotyping. WB contributed in SNP discovery and provided bioinformatic support in SNP positioning. RDS provided samples and edited the manuscript. JFT provided samples and edited the manuscript. HWR is project leader and contributed in project concept, design, interpretation and manuscript preparation.

## Supplementary Material

Additional file 1 **Figure S1**. Frequency distribution of MAF for 9,195 SNPs used in the LD analysis. **Figure S2**. Comparison of *Dvol *and *D' *computed for non-syntenic SNP pairs. **Figure S3**. Relationship between mean minor allelic frequency (MAF) and *D*' for non-syntenic SNP pairs. **Figure S4**. Relationship between mean minor allelic frequency and *r*^2 ^for non-syntenic SNP pairs. **Figure S5**. Relationship between mean minor allelic frequency and *Dvol *for non-syntenic SNP pairs. **Figure S6**. Mean *D' *at different physical distances pooled over all the autosomes and estimated using different sample sizes. The numbers with n_ in legend are the sample sizes randomly drawn from a total of 1000. The n_1546 are all the bulls genotyped in the present study. **Figure S7**. Mean *r*^2 ^at different physical distances pooled over all autosomes and estimated using different sample sizes. The numbers with n_ in legend are the sample sizes randomly drawn from a total of 1000. The n_1546 are all the bulls genotyped in the present study. Click here for file

Additional file 2 **Figures S8**. Positions and MAF of the SNPs included in present studies. Click here for file

Additional file 3 **Table S1**. Mean linkage disequilibrium among syntenic SNPs over different map distances on each autosome (1–29). Click here for file

Additional file 4 **Figure S9**. Distribution of *D' *between SNP pairs in relation to the physical distance between loci (Mb), for individual autosomes (1–29). The red line shows average *D' *in each 500 kb sliding window. The blue line shows the theoretical distribution from the Malécot model. Click here for file

Additional file 5 **Figure S10**. Distribution of *r*^2 ^between SNP pairs in relation to the physical distance (Mb) between loci, for individual autosomes (1–29). The red line shows average *r*^2 ^in each 500 kb sliding window. Click here for file

Additional file 6 **Figure S11**. Linkage disequilibrium (*D'*) between SNPs on all the bovine autosomal chromosomes (BTA1-BTA29) presented in the form of heatmap of *D' *. All SNP with the MAF of less than 0.05 and showing deviation from HWE were excluded in the LD measurement. These figures were prepared by Haploview software. Here bright red color indicates *D' *= 1 with LOD ≥ 2, blue *D' *= 1 with LOD < 2, shades of pink/red *D' *< 1 with LOD ≥ 2 and white *D' *< 1 with LOD < 2. Click here for file

Additional file 7 **Figure S12**. Linkage disequilibrium (*r*^2^) between SNPs on all the bovine autosomal chromosomes (BTA1-BTA29) presented in the form of heatmap of *r*^2 ^. All SNP with the MAF of less than 0.05 and showing deviation from HWE were excluded in the LD measurement. These figures were prepared by Haploview software. Here black color indicates *r*^2 ^= 1, shades of grey 0 <*r*^2 ^< 1 and white *r*^2 ^= 0. Click here for file

Additional file 8 **Table S2**. Comparison of pair-wise estimates *D' *and *Dvol *for two chromosomes obtained from different sample sizes and compared against a reference sample of 1000 animals. Click here for file
