# Supplementary material for: Extent of genome-wide linkage disequilibrium in Australian Holstein-Friesian cattle based on a high-density SNP panel
Source: BMC Genomics. 2008 Apr 24;9:187. doi: 10.1186/1471-2164-9-187 (PMC2386485; doi:10.1186/1471-2164-9-187)
Supplement: Additional file 1 — Figure S1. Frequency distribution of MAF for 9,195 SNPs used in the LD analysis. Figure S2. Comparison of Dvol and D' computed for non-syntenic SNP pairs. Figure S3. Relationship between mean minor allelic frequency (MAF) and D' for non-syntenic SNP pairs. Figure S4. Relationship between mean minor allelic frequency and r2 for non-syntenic SNP pairs. Figure S5. Relationship between mean minor allelic frequency and Dvol for non-syntenic SNP pairs. Figure S6. Mean D' at different physical distances pooled over all the autosomes and estimated using different sample sizes. The numbers with n_ in legend are the sample sizes randomly drawn from a total of 1000. The n_1546 are all the bulls genotyped in the present study. Figure S7. Mean r2 at different physical distances pooled over all autosomes and estimated using different sample sizes. The numbers with n_ in legend are the sample sizes randomly drawn from a total of 1000. The n_1546 are all the bulls genotyped in the present study. [file 1471-2164-9-187-S1.doc]

Figure S1. Frequency distribution of MAF for the 9,195 SNPs used in the LD analysis.


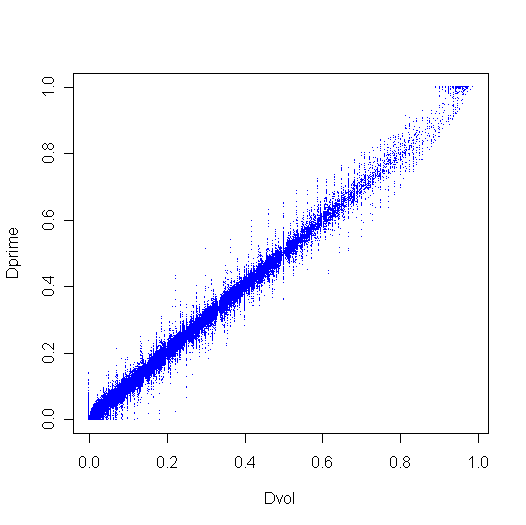


Figure S2. Comparison of *Dvol* and *D'* computed for non-syntenic SNP pairs.


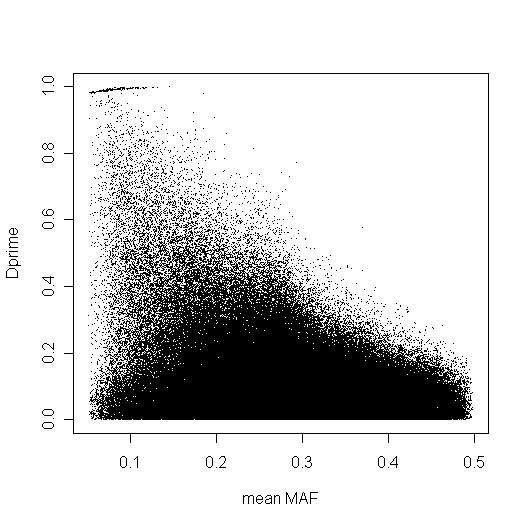


Figure S3. Relationship between mean minor allelic frequency (MAF) and *D*′ for non-syntenic SNP pairs.


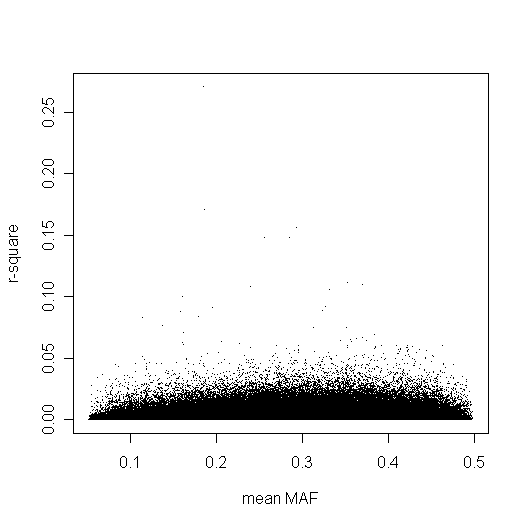


Figure S4. Relationship between mean minor allelic frequency and *r*^2^ for non-syntenic SNP pairs.


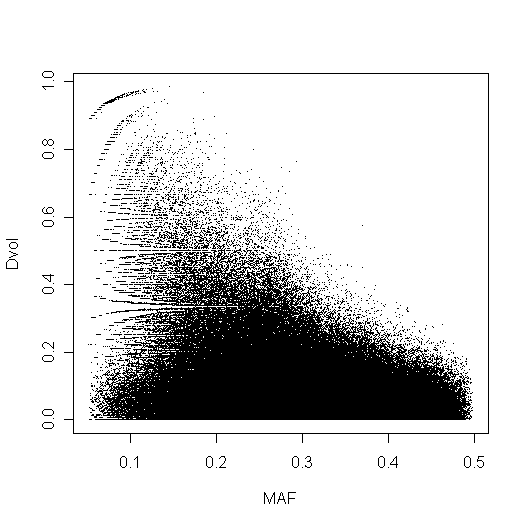


Figure S5. Relationship between mean minor allelic frequency and *Dvol* for non-syntenic SNP pairs.

Figure S6. Mean *D'* at different physical distances pooled over all the autosomes and estimated using different sample sizes. The numbers with n_ in legend are the sample sizes randomly drawn from a total of 1000. The n_1546 are all the bulls genotyped in the present study.

Figure S7. Mean *r^2^* at different physical distances pooled over all autosomes and estimated using different sample sizes. The numbers with n_ in legend are the sample sizes randomly drawn from a total of 1000. The n_1546 are all the bulls genotyped in the present study.
