# Supplementary material for: Extent of genome-wide linkage disequilibrium in Australian Holstein-Friesian cattle based on a high-density SNP panel
Source: BMC Genomics. 2008 Apr 24;9:187. doi: 10.1186/1471-2164-9-187 (PMC2386485; doi:10.1186/1471-2164-9-187)
Supplement: Additional file 2 — Figures S8. Positions and MAF of the SNPs included in present studies. [file 1471-2164-9-187-S2.pdf]

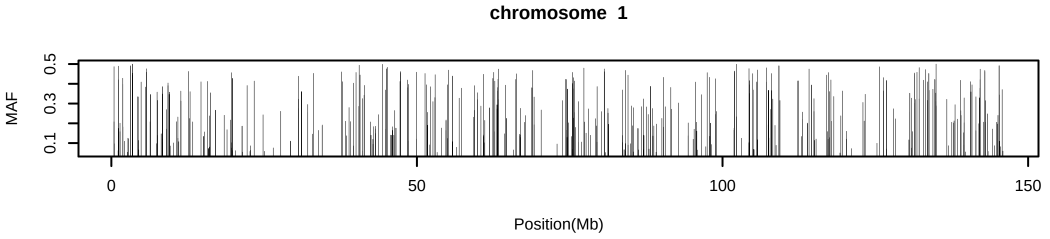
chromosome 1
MAF
0.1
0.3
0.5
0
50
100
150
Position(Mb)

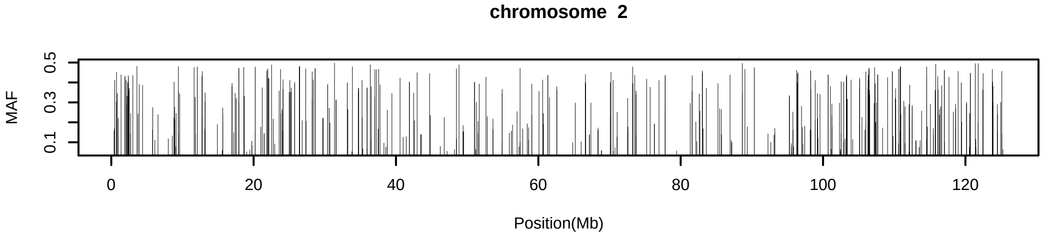
chromosome 2
MAF
0.1
0.3
0.5
0
20
40
60
80
100
120
Position(Mb)

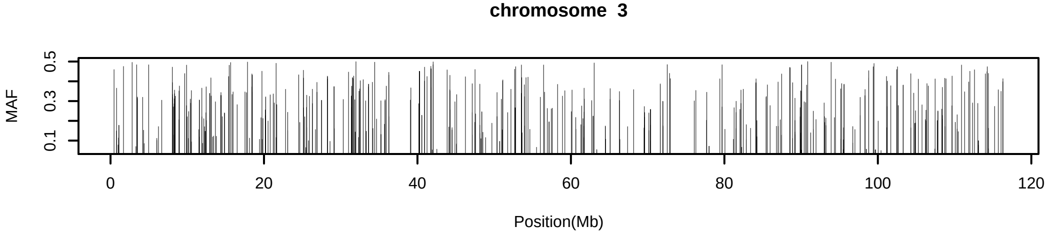
chromosome 3
MAF
0.1
0.3
0.5
0
20
40
60
80
100
120
Position(Mb)

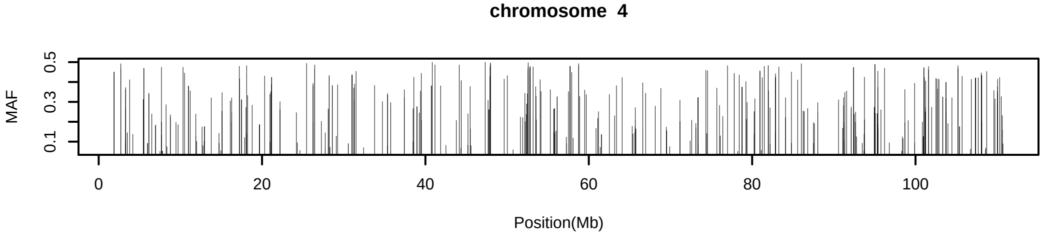
chromosome 4
MAF
0.1
0.3
0.5
0
20
40
60
80
100
Position(Mb)

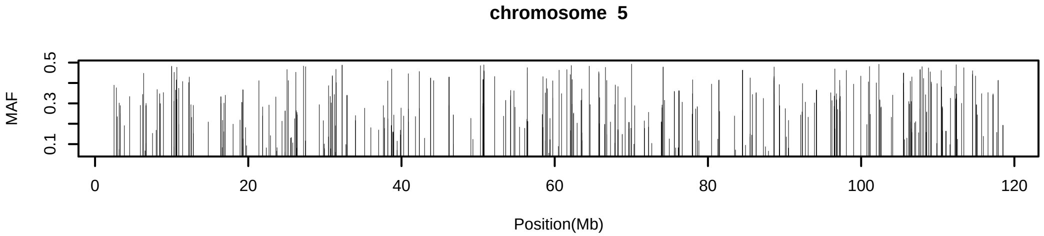
chromosome 5
MAF
0.1
0.3
0.5
0
20
40
60
80
100
120
Position(Mb)

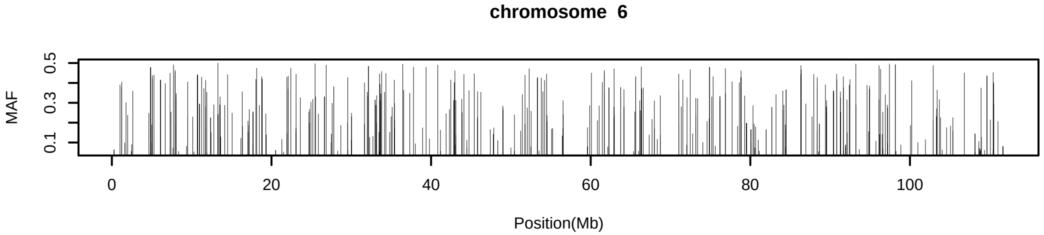
chromosome 6
MAF
0.1
0.3
0.5
0
20
40
60
80
100
Position(Mb)

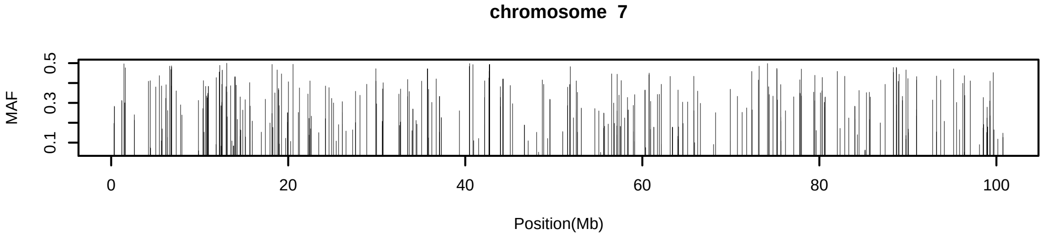
chromosome 7
MAF
0.1
0.3
0.5
0
20
40
60
80
100
Position(Mb)

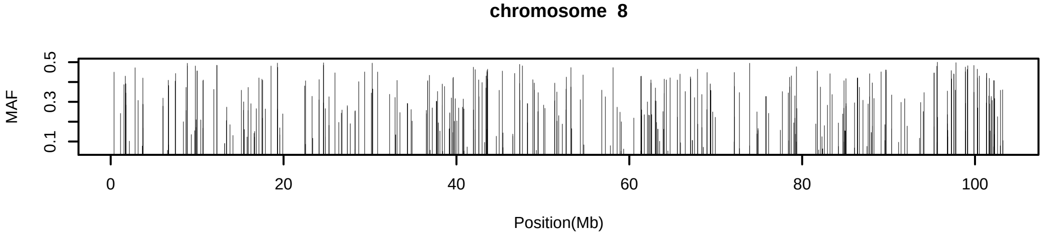
chromosome 8
MAF
0.1
0.3
0.5
0
20
40
60
80
100
Position(Mb)

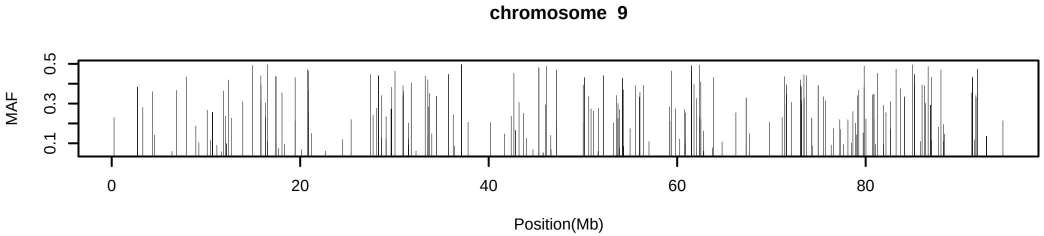
chromosome 9
MAF
0.1
0.3
0.5
0
20
40
60
80
Position(Mb)

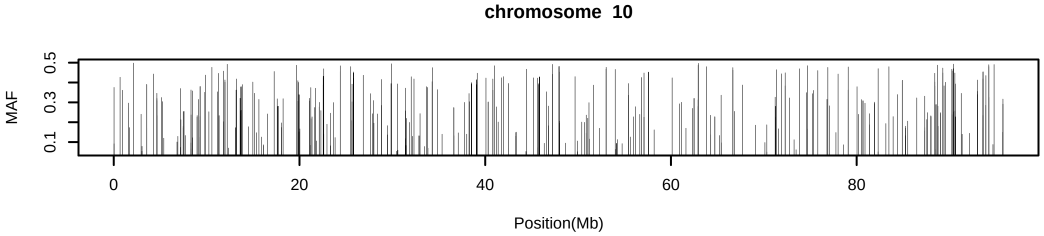
chromosome 10
MAF
0.1
0.3
0.5
0
20
40
60
80
Position(Mb)

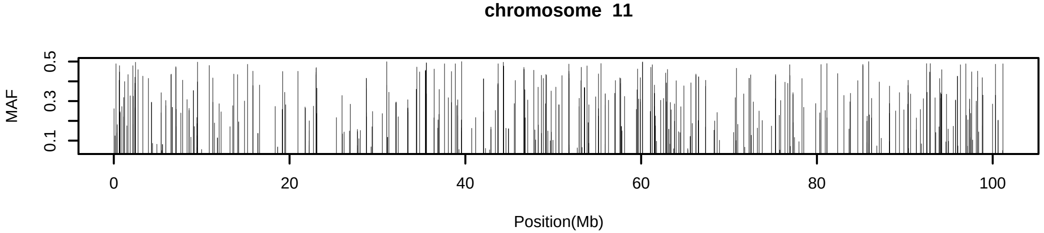
chromosome 11
MAF
0.1
0.3
0.5
0
20
40
60
80
100
Position(Mb)

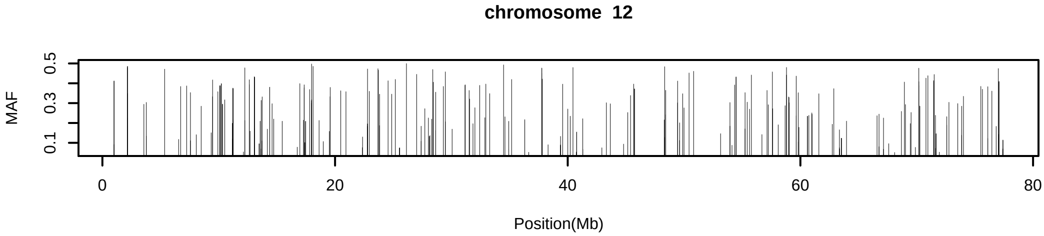
chromosome 12
MAF
0.1
0.3
0.5
0
20
40
60
80
Position(Mb)

chromosome 13

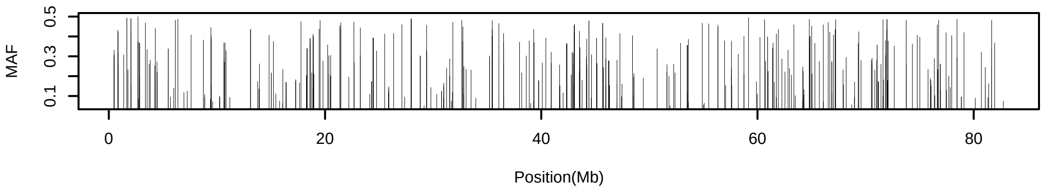

chromosome 14

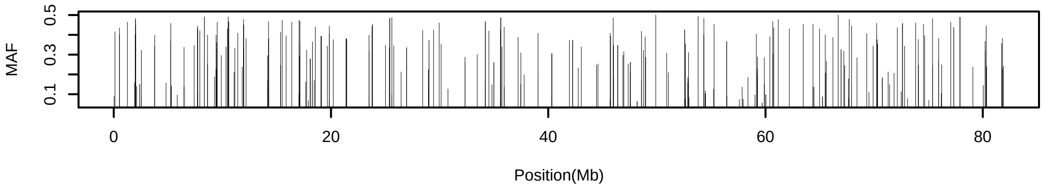

chromosome 15

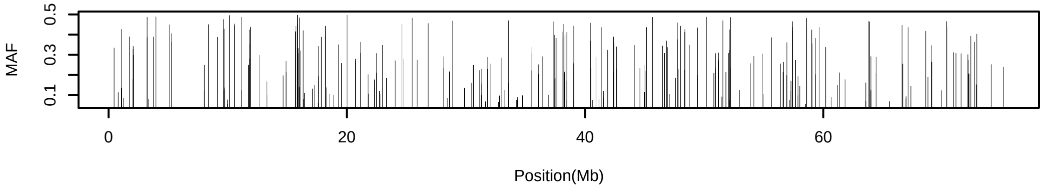

chromosome 16

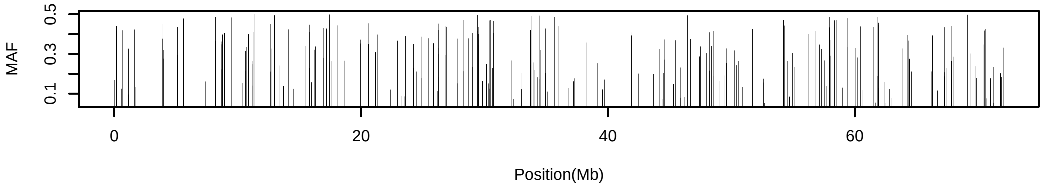

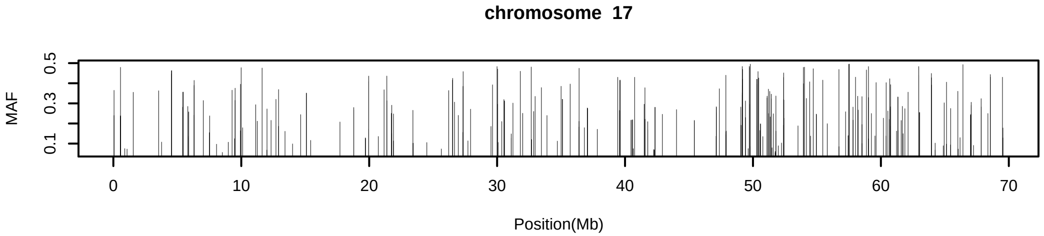
chromosome 17
MAF
0.1
0.3
0.5
0
10
20
30
40
50
60
70
Position(Mb)

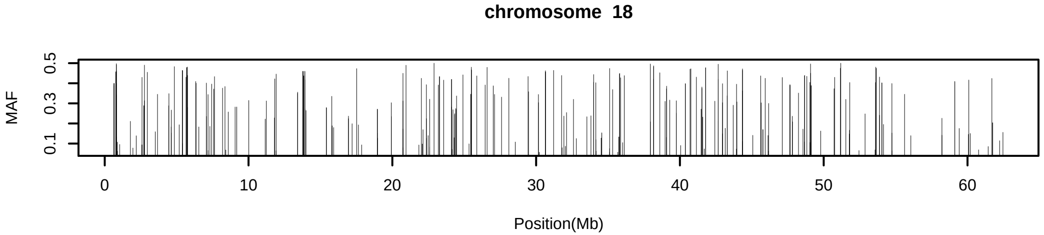
chromosome 18
MAF
0.1
0.3
0.5
0
10
20
30
40
50
60
Position(Mb)

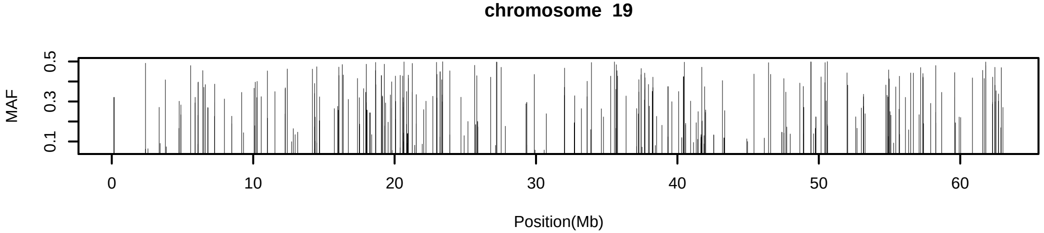
chromosome 19
MAF
0.1
0.3
0.5
0
10
20
30
40
50
60
Position(Mb)

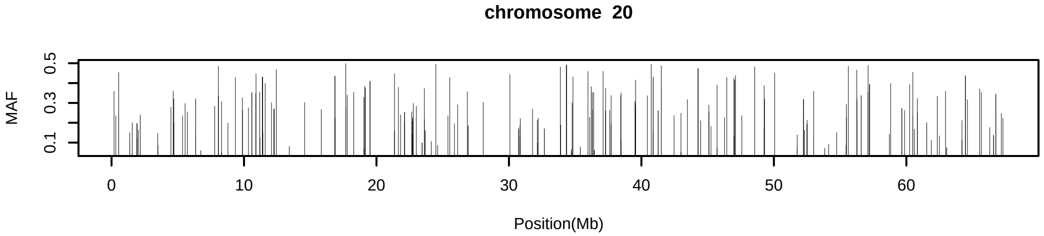
chromosome 20
MAF
0.1
0.3
0.5
0
10
20
30
40
50
60
Position(Mb)

chromosome 21

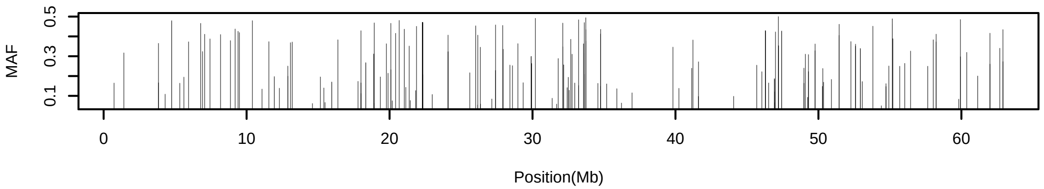

chromosome 22

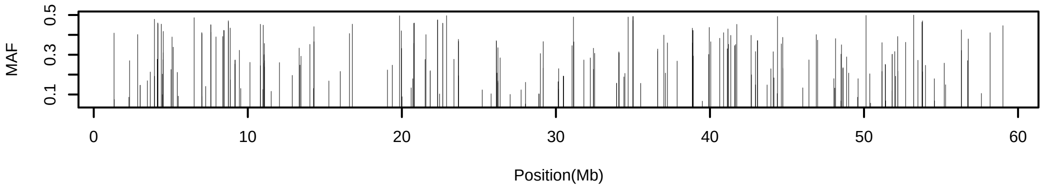

chromosome 23

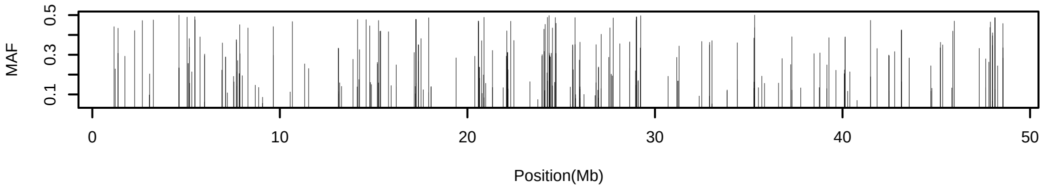

chromosome 24

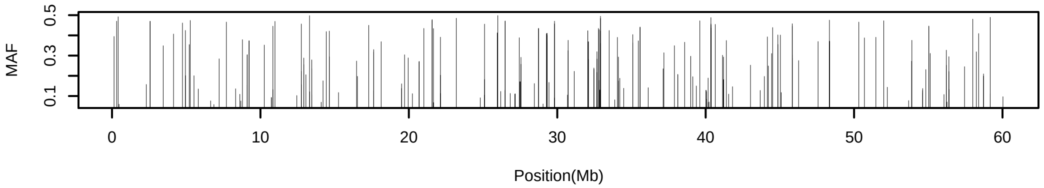

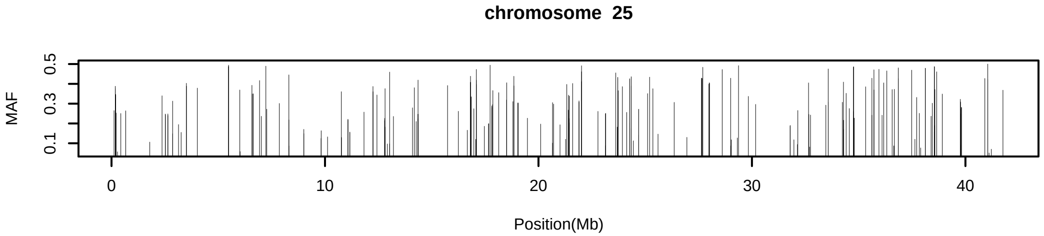
chromosome 25
MAF
0.1
0.3
0.5
0
10
20
30
40
Position(Mb)

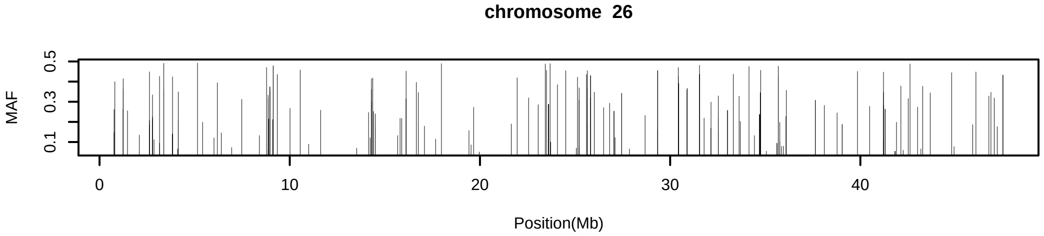
chromosome 26
MAF
0.1
0.3
0.5
0
10
20
30
40
Position(Mb)

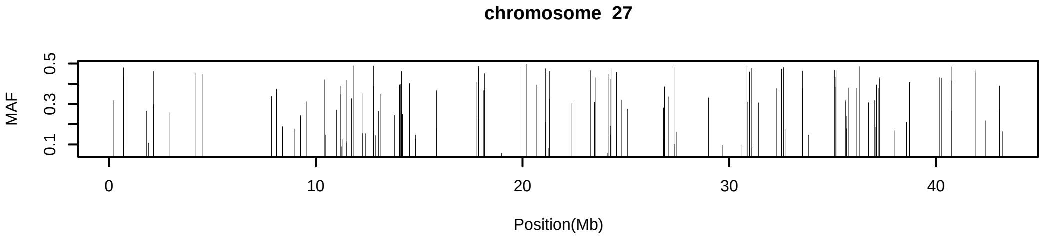
chromosome 27
MAF
0.1
0.3
0.5
0
10
20
30
40
Position(Mb)

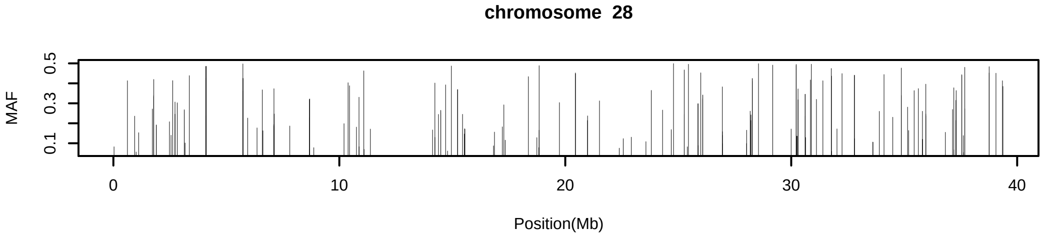
chromosome 28
MAF
0.1
0.3
0.5
0
10
20
30
40
Position(Mb)

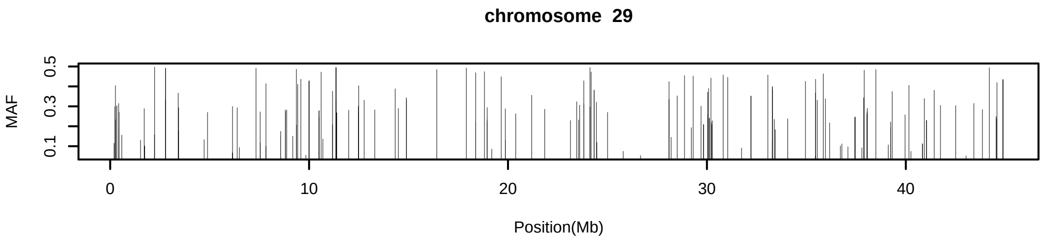

chromosome 29
MAF
0.1
0.3
0.5
0
10
20
30
40
Position(Mb)
