# Supplementary material for: Extent of genome-wide linkage disequilibrium in Australian Holstein-Friesian cattle based on a high-density SNP panel
Source: BMC Genomics. 2008 Apr 24;9:187. doi: 10.1186/1471-2164-9-187 (PMC2386485; doi:10.1186/1471-2164-9-187)
Supplement: Additional file 3 — Table S1. Mean linkage disequilibrium among syntenic SNPs over different map distances on each autosome (1–29). [file 1471-2164-9-187-S3.doc]

Table S1. Mean linkage disequilibrium measures (*D'* and *r^2^*  ) among syntenic SNPs over different map distances for individual chromosome (1-29).

BTA1

| Distance | N | Mean *D'* | SD *(D')* | Median *D'* | Mean *r^2^* | SD *(r^2^)* | Median *r^2^* |
| --- | --- | --- | --- | --- | --- | --- | --- |
| 0- 1 kb | 259 | 0.9866 | 0.0667 | 1.0000 | 0.7703 | 0.3409 | 0.9930 |
| 1-10 kb | 59 | 0.9556 | 0.1624 | 1.0000 | 0.7724 | 0.3509 | 0.9900 |
| 10-20 kb | 34 | 0.7542 | 0.2817 | 0.8735 | 0.3786 | 0.3530 | 0.3260 |
| 20-40 kb | 45 | 0.8512 | 0.1952 | 0.9060 | 0.3750 | 0.2884 | 0.2470 |
| 40-60 kb | 41 | 0.7819 | 0.2572 | 0.9350 | 0.2067 | 0.1638 | 0.1910 |
| 60-100 kb | 100 | 0.7306 | 0.2766 | 0.8405 | 0.1591 | 0.1886 | 0.0675 |
| 100-200 kb | 239 | 0.5519 | 0.3242 | 0.5290 | 0.0856 | 0.1147 | 0.0330 |
| 200-500 kb | 556 | 0.4644 | 0.3108 | 0.3905 | 0.0668 | 0.1131 | 0.0270 |
| 0.5-1 Mb | 1087 | 0.4077 | 0.2777 | 0.3420 | 0.0549 | 0.0998 | 0.0240 |
| 1-2 Mb | 1905 | 0.4098 | 0.2825 | 0.3630 | 0.0525 | 0.0792 | 0.0240 |
| 2-5 Mb | 5145 | 0.3795 | 0.2737 | 0.3330 | 0.0411 | 0.0663 | 0.0180 |
| 5-10 Mb | 8287 | 0.3131 | 0.2480 | 0.2520 | 0.0280 | 0.0480 | 0.0120 |
| 10-20 Mb | 14653 | 0.2537 | 0.2130 | 0.1970 | 0.0169 | 0.0280 | 0.0070 |
| 20-50 | 40830 | 0.1658 | 0.1630 | 0.1160 | 0.0072 | 0.0126 | 0.0030 |
| > 50 Mb | 62220 | 0.1163 | 0.1260 | 0.0780 | 0.0033 | 0.0054 | 0.0010 |

BTA2

| Distance | N | Mean *D'* | SD *(D')* | Median *D'* | Mean *r^2^* | SD *(r^2^)* | Median *r^2^* |
| --- | --- | --- | --- | --- | --- | --- | --- |
| 0- 1 kb | 216 | 0.9930 | 0.0585 | 1.0000 | 0.8014 | 0.3170 | 0.9930 |
| 1-10 kb | 45 | 0.9704 | 0.0963 | 1.0000 | 0.6223 | 0.3447 | 0.7780 |
| 10-20 kb | 22 | 0.9167 | 0.2133 | 1.0000 | 0.4551 | 0.3658 | 0.4125 |
| 20-40 kb | 44 | 0.6757 | 0.3078 | 0.7720 | 0.2101 | 0.2461 | 0.1390 |
| 40-60 kb | 22 | 0.7807 | 0.2643 | 0.8530 | 0.2095 | 0.2824 | 0.0850 |
| 60-100 kb | 66 | 0.6220 | 0.3074 | 0.5695 | 0.1786 | 0.2026 | 0.0840 |
| 100-200 kb | 165 | 0.4458 | 0.3398 | 0.3480 | 0.0781 | 0.1309 | 0.0460 |
| 200-500 kb | 612 | 0.4245 | 0.2970 | 0.3530 | 0.0701 | 0.0930 | 0.0400 |
| 0.5-1 Mb | 861 | 0.4199 | 0.2889 | 0.3600 | 0.0564 | 0.0717 | 0.0300 |
| 1-2 Mb | 1583 | 0.3885 | 0.2904 | 0.3160 | 0.0488 | 0.0722 | 0.0230 |
| 2-5 Mb | 4890 | 0.3452 | 0.2585 | 0.2850 | 0.0388 | 0.0563 | 0.0200 |
| 5-10 Mb | 7899 | 0.2915 | 0.2313 | 0.2330 | 0.0275 | 0.0404 | 0.0130 |
| 10-20 Mb | 13855 | 0.2207 | 0.2013 | 0.1580 | 0.0141 | 0.0224 | 0.0060 |
| 20-50 | 30247 | 0.1544 | 0.1533 | 0.1080 | 0.0067 | 0.0103 | 0.0030 |
| > 50 Mb | 42304 | 0.1116 | 0.1192 | 0.0760 | 0.0036 | 0.0052 | 0.0020 |

BTA3

| Distance | N | Mean *D'* | SD *(D')* | Median *D'* | Mean *r^2^* | SD *(r^2^)* | Median *r^2^* |
| --- | --- | --- | --- | --- | --- | --- | --- |
| 0- 1 kb | 176 | 0.9858 | 0.0830 | 1.0000 | 0.7857 | 0.3347 | 0.9925 |
| 1-10 kb | 51 | 0.9690 | 0.1316 | 1.0000 | 0.5705 | 0.3779 | 0.5600 |
| 10-20 kb | 17 | 0.9736 | 0.0740 | 1.0000 | 0.4273 | 0.3233 | 0.3710 |
| 20-40 kb | 68 | 0.8707 | 0.2742 | 0.9940 | 0.4043 | 0.3391 | 0.2365 |
| 40-60 kb | 39 | 0.7404 | 0.3126 | 0.8950 | 0.1406 | 0.1981 | 0.0840 |
| 60-100 kb | 89 | 0.6180 | 0.3440 | 0.6140 | 0.1507 | 0.2144 | 0.0370 |
| 100-200 kb | 181 | 0.5573 | 0.3442 | 0.5640 | 0.1113 | 0.2008 | 0.0420 |
| 200-500 kb | 572 | 0.5085 | 0.3319 | 0.4680 | 0.0767 | 0.1134 | 0.0390 |
| 0.5-1 Mb | 874 | 0.4267 | 0.3078 | 0.3545 | 0.0613 | 0.0852 | 0.0360 |
| 1-2 Mb | 1903 | 0.4187 | 0.3009 | 0.3810 | 0.0690 | 0.1273 | 0.0260 |
| 2-5 Mb | 5387 | 0.3673 | 0.2648 | 0.3230 | 0.0431 | 0.0625 | 0.0210 |
| 5-10 Mb | 9002 | 0.2958 | 0.2391 | 0.2340 | 0.0265 | 0.0404 | 0.0120 |
| 10-20 Mb | 15610 | 0.2155 | 0.1959 | 0.1560 | 0.0144 | 0.0236 | 0.0060 |
| 20-50 | 36631 | 0.1431 | 0.1462 | 0.0980 | 0.0057 | 0.0090 | 0.0020 |
| > 50 Mb | 36816 | 0.1062 | 0.1164 | 0.0710 | 0.0029 | 0.0042 | 0.0010 |

BTA4

| Distance | N | Mean *D'* | SD *(D')* | Median *D'* | Mean *r^2^* | SD *(r^2^)* | Median *r^2^* |
| --- | --- | --- | --- | --- | --- | --- | --- |
| 0- 1 kb | 148 | 0.9638 | 0.1532 | 1.0000 | 0.7875 | 0.3332 | 0.9935 |
| 1-10 kb | 63 | 0.9370 | 0.1599 | 1.0000 | 0.5652 | 0.3981 | 0.6650 |
| 10-20 kb | 16 | 0.9994 | 0.0016 | 1.0000 | 0.2429 | 0.2073 | 0.1930 |
| 20-40 kb | 22 | 0.8645 | 0.1503 | 0.9125 | 0.2906 | 0.3322 | 0.1315 |
| 40-60 kb | 28 | 0.5926 | 0.3278 | 0.6185 | 0.2378 | 0.2809 | 0.0520 |
| 60-100 kb | 49 | 0.5256 | 0.3374 | 0.4740 | 0.1423 | 0.2400 | 0.0530 |
| 100-200 kb | 126 | 0.5323 | 0.3262 | 0.4750 | 0.0992 | 0.1675 | 0.0525 |
| 200-500 kb | 474 | 0.4634 | 0.2981 | 0.4320 | 0.0744 | 0.0860 | 0.0500 |
| 0.5-1 Mb | 574 | 0.4020 | 0.2751 | 0.3375 | 0.0577 | 0.0821 | 0.0270 |
| 1-2 Mb | 1239 | 0.3776 | 0.2890 | 0.3300 | 0.0396 | 0.0577 | 0.0180 |
| 2-5 Mb | 3652 | 0.3236 | 0.2590 | 0.2560 | 0.0335 | 0.0494 | 0.0160 |
| 5-10 Mb | 6059 | 0.2779 | 0.2266 | 0.2210 | 0.0246 | 0.0365 | 0.0110 |
| 10-20 Mb | 10281 | 0.2290 | 0.2042 | 0.1690 | 0.0164 | 0.0273 | 0.0070 |
| 20-50 | 25176 | 0.1532 | 0.1504 | 0.1080 | 0.0071 | 0.0116 | 0.0030 |
| > 50 Mb | 23724 | 0.1172 | 0.1211 | 0.0830 | 0.0039 | 0.0059 | 0.0020 |

BTA5

| Distance | N | Mean *D'* | SD *(D')* | Median *D'* | Mean *r^2^* | SD *(r^2^)* | Median *r^2^* |
| --- | --- | --- | --- | --- | --- | --- | --- |
| 0- 1 kb | 171 | 0.9976 | 0.0094 | 1.0000 | 0.7675 | 0.3221 | 0.9910 |
| 1-10 kb | 56 | 0.9251 | 0.2516 | 1.0000 | 0.4341 | 0.3781 | 0.2770 |
| 10-20 kb | 23 | 0.8859 | 0.1902 | 1.0000 | 0.2557 | 0.1759 | 0.2800 |
| 20-40 kb | 34 | 0.8362 | 0.1989 | 0.9235 | 0.3505 | 0.2937 | 0.2100 |
| 40-60 kb | 11 | 0.9027 | 0.1056 | 0.9160 | 0.2639 | 0.1955 | 0.2120 |
| 60-100 kb | 69 | 0.6328 | 0.3321 | 0.6470 | 0.1836 | 0.2380 | 0.1110 |
| 100-200 kb | 170 | 0.5508 | 0.3505 | 0.5595 | 0.1038 | 0.1620 | 0.0365 |
| 200-500 kb | 438 | 0.4942 | 0.3113 | 0.4565 | 0.0819 | 0.1216 | 0.0350 |
| 0.5-1 Mb | 723 | 0.4677 | 0.2979 | 0.4400 | 0.0726 | 0.0998 | 0.0320 |
| 1-2 Mb | 1307 | 0.4187 | 0.3017 | 0.3410 | 0.0545 | 0.0804 | 0.0270 |
| 2-5 Mb | 4251 | 0.3515 | 0.2713 | 0.2940 | 0.0400 | 0.0628 | 0.0180 |
| 5-10 Mb | 6809 | 0.2940 | 0.2361 | 0.2390 | 0.0262 | 0.0395 | 0.0120 |
| 10-20 Mb | 12267 | 0.2189 | 0.1998 | 0.1580 | 0.0146 | 0.0260 | 0.0060 |
| 20-50 | 30939 | 0.1421 | 0.1428 | 0.0980 | 0.0056 | 0.0087 | 0.0030 |
| > 50 Mb | 28223 | 0.1108 | 0.1206 | 0.0740 | 0.0043 | 0.0155 | 0.0010 |

BTA6

| Distance | N | Mean *D'* | SD *(D')* | Median *D'* | Mean *r^2^* | SD *(r^2^)* | Median *r^2^* |
| --- | --- | --- | --- | --- | --- | --- | --- |
| 0- 1 kb | 185 | 0.9962 | 0.0205 | 1.0000 | 0.7400 | 0.3461 | 0.9780 |
| 1-10 kb | 85 | 0.8882 | 0.2714 | 1.0000 | 0.5991 | 0.4214 | 0.7270 |
| 10-20 kb | 29 | 0.9472 | 0.1508 | 1.0000 | 0.5343 | 0.3927 | 0.5320 |
| 20-40 kb | 52 | 0.8721 | 0.2239 | 0.9975 | 0.4167 | 0.3705 | 0.3275 |
| 40-60 kb | 54 | 0.5758 | 0.3908 | 0.3975 | 0.1794 | 0.2927 | 0.0510 |
| 60-100 kb | 41 | 0.6102 | 0.3345 | 0.5540 | 0.0790 | 0.0864 | 0.0400 |
| 100-200 kb | 122 | 0.5656 | 0.3662 | 0.6180 | 0.1094 | 0.1598 | 0.0460 |
| 200-500 kb | 532 | 0.4728 | 0.3211 | 0.4140 | 0.0671 | 0.0966 | 0.0300 |
| 0.5-1 Mb | 858 | 0.3852 | 0.3084 | 0.2845 | 0.0474 | 0.0717 | 0.0200 |
| 1-2 Mb | 1712 | 0.3434 | 0.2727 | 0.2620 | 0.0410 | 0.0675 | 0.0140 |
| 2-5 Mb | 4561 | 0.3462 | 0.2802 | 0.2650 | 0.0347 | 0.0601 | 0.0130 |
| 5-10 Mb | 7832 | 0.2943 | 0.2472 | 0.2210 | 0.0247 | 0.0432 | 0.0100 |
| 10-20 Mb | 14752 | 0.2209 | 0.2020 | 0.1610 | 0.0136 | 0.0224 | 0.0060 |
| 20-50 | 33484 | 0.1483 | 0.1540 | 0.1010 | 0.0060 | 0.0141 | 0.0020 |
| > 50 Mb | 30531 | 0.1145 | 0.1235 | 0.0770 | 0.0030 | 0.0044 | 0.0010 |

BTA7

| Distance | N | Mean *D'* | SD *(D')* | Median *D'* | Mean *r^2^* | SD *(r^2^)* | Median *r^2^* |
| --- | --- | --- | --- | --- | --- | --- | --- |
| 0- 1 kb | 106 | 0.9951 | 0.0220 | 1.0000 | 0.7825 | 0.3110 | 0.9910 |
| 1-10 kb | 46 | 0.9683 | 0.1531 | 1.0000 | 0.6020 | 0.3878 | 0.6210 |
| 10-20 kb | 21 | 0.9705 | 0.1008 | 1.0000 | 0.4106 | 0.3884 | 0.2750 |
| 20-40 kb | 48 | 0.8383 | 0.2551 | 0.9890 | 0.3853 | 0.3620 | 0.2235 |
| 40-60 kb | 36 | 0.7861 | 0.2666 | 0.8410 | 0.2896 | 0.3015 | 0.2220 |
| 60-100 kb | 51 | 0.6101 | 0.3349 | 0.7070 | 0.1721 | 0.2377 | 0.0690 |
| 100-200 kb | 123 | 0.5090 | 0.3277 | 0.4060 | 0.0912 | 0.1143 | 0.0450 |
| 200-500 kb | 370 | 0.4450 | 0.2902 | 0.4000 | 0.0744 | 0.1099 | 0.0410 |
| 0.5-1 Mb | 659 | 0.4514 | 0.2882 | 0.4230 | 0.0702 | 0.0942 | 0.0360 |
| 1-2 Mb | 1364 | 0.4127 | 0.2722 | 0.3915 | 0.0601 | 0.0827 | 0.0300 |
| 2-5 Mb | 3496 | 0.3551 | 0.2647 | 0.2900 | 0.0442 | 0.0644 | 0.0200 |
| 5-10 Mb | 6100 | 0.2771 | 0.2228 | 0.2220 | 0.0266 | 0.0420 | 0.0120 |
| 10-20 Mb | 10024 | 0.2034 | 0.1793 | 0.1510 | 0.0142 | 0.0225 | 0.0060 |
| 20-50 | 24545 | 0.1267 | 0.1213 | 0.0920 | 0.0056 | 0.0099 | 0.0020 |
| > 50 Mb | 15846 | 0.0958 | 0.0996 | 0.0670 | 0.0029 | 0.0042 | 0.0010 |

BTA8

| Distance | N | Mean *D'* | SD *(D')* | Median *D'* | Mean *r^2^* | SD *(r^2^)* | Median *r^2^* |
| --- | --- | --- | --- | --- | --- | --- | --- |
| 0- 1 kb | 122 | 0.9708 | 0.1476 | 1.0000 | 0.7638 | 0.3336 | 0.9855 |
| 1-10 kb | 45 | 0.9756 | 0.1349 | 1.0000 | 0.5317 | 0.3430 | 0.3760 |
| 10-20 kb | 20 | 0.9926 | 0.0178 | 1.0000 | 0.2694 | 0.1438 | 0.2445 |
| 20-40 kb | 50 | 0.8760 | 0.2312 | 0.9890 | 0.2051 | 0.2462 | 0.1000 |
| 40-60 kb | 42 | 0.4614 | 0.2778 | 0.4450 | 0.0900 | 0.1299 | 0.0550 |
| 60-100 kb | 67 | 0.6587 | 0.3175 | 0.6780 | 0.1688 | 0.1903 | 0.1100 |
| 100-200 kb | 130 | 0.4922 | 0.3668 | 0.4470 | 0.0905 | 0.1537 | 0.0280 |
| 200-500 kb | 445 | 0.4216 | 0.3098 | 0.3530 | 0.0644 | 0.0995 | 0.0240 |
| 0.5-1 Mb | 686 | 0.3785 | 0.2889 | 0.3290 | 0.0492 | 0.0742 | 0.0190 |
| 1-2 Mb | 1537 | 0.3623 | 0.2668 | 0.3060 | 0.0517 | 0.0765 | 0.0230 |
| 2-5 Mb | 4115 | 0.3193 | 0.2607 | 0.2490 | 0.0334 | 0.0538 | 0.0140 |
| 5-10 Mb | 6231 | 0.2811 | 0.2383 | 0.2120 | 0.0269 | 0.0427 | 0.0100 |
| 10-20 Mb | 11783 | 0.2425 | 0.2111 | 0.1830 | 0.0190 | 0.0314 | 0.0080 |
| 20-50 | 28276 | 0.1542 | 0.1511 | 0.1080 | 0.0073 | 0.0118 | 0.0030 |
| > 50 Mb | 20756 | 0.1060 | 0.1100 | 0.0740 | 0.0033 | 0.0050 | 0.0020 |

BTA9

| Distance | N | Mean *D'* | SD *(D')* | Median *D'* | Mean *r^2^* | SD *(r^2^)* | Median *r^2^* |
| --- | --- | --- | --- | --- | --- | --- | --- |
| 0- 1 kb | 113 | 0.9848 | 0.0973 | 1.0000 | 0.8181 | 0.3208 | 0.9960 |
| 1-10 kb | 24 | 0.9005 | 0.2593 | 0.9980 | 0.6524 | 0.4039 | 0.8870 |
| 10-20 kb | 26 | 0.7911 | 0.2454 | 0.8645 | 0.3464 | 0.2362 | 0.2875 |
| 20-40 kb | 13 | 0.7290 | 0.3507 | 0.9370 | 0.1826 | 0.2046 | 0.1040 |
| 40-60 kb | 14 | 0.4171 | 0.2835 | 0.2440 | 0.0305 | 0.0180 | 0.0255 |
| 60-100 kb | 23 | 0.7261 | 0.2697 | 0.8550 | 0.2747 | 0.1295 | 0.2930 |
| 100-200 kb | 68 | 0.5723 | 0.3080 | 0.6260 | 0.1153 | 0.1557 | 0.0350 |
| 200-500 kb | 226 | 0.4618 | 0.3075 | 0.4200 | 0.0680 | 0.1059 | 0.0285 |
| 0.5-1 Mb | 371 | 0.4258 | 0.2869 | 0.4040 | 0.0538 | 0.0695 | 0.0350 |
| 1-2 Mb | 783 | 0.3619 | 0.2594 | 0.3250 | 0.0445 | 0.0662 | 0.0210 |
| 2-5 Mb | 2093 | 0.3362 | 0.2624 | 0.2740 | 0.0364 | 0.0562 | 0.0160 |
| 5-10 Mb | 3433 | 0.2912 | 0.2256 | 0.2400 | 0.0302 | 0.0451 | 0.0140 |
| 10-20 Mb | 6378 | 0.2254 | 0.1930 | 0.1690 | 0.0182 | 0.0303 | 0.0080 |
| 20-50 | 13715 | 0.1575 | 0.1450 | 0.1180 | 0.0082 | 0.0131 | 0.0040 |
| > 50 Mb | 7436 | 0.1101 | 0.1163 | 0.0760 | 0.0033 | 0.0045 | 0.0020 |

BTA10

| Distance | N | Mean *D'* | SD *(D')* | Median *D'* | Mean *r^2^* | SD *(r^2^)* | Median *r^2^* |
| --- | --- | --- | --- | --- | --- | --- | --- |
| 0- 1 kb | 165 | 0.9831 | 0.0746 | 1.0000 | 0.7112 | 0.3381 | 0.9080 |
| 1-10 kb | 72 | 0.9747 | 0.0878 | 1.0000 | 0.6294 | 0.3639 | 0.7885 |
| 10-20 kb | 27 | 0.9120 | 0.1795 | 1.0000 | 0.4272 | 0.3447 | 0.3200 |
| 20-40 kb | 29 | 0.8256 | 0.1451 | 0.8290 | 0.2543 | 0.2578 | 0.1420 |
| 40-60 kb | 38 | 0.7053 | 0.3444 | 0.8695 | 0.1841 | 0.1620 | 0.1585 |
| 60-100 kb | 61 | 0.7574 | 0.3195 | 0.9360 | 0.2967 | 0.3050 | 0.1790 |
| 100-200 kb | 178 | 0.5523 | 0.3235 | 0.5290 | 0.1441 | 0.1832 | 0.0690 |
| 200-500 kb | 409 | 0.4389 | 0.2932 | 0.3880 | 0.0623 | 0.0782 | 0.0340 |
| 0.5-1 Mb | 849 | 0.4364 | 0.2968 | 0.3850 | 0.0737 | 0.0970 | 0.0350 |
| 1-2 Mb | 1643 | 0.3967 | 0.2768 | 0.3460 | 0.0582 | 0.0908 | 0.0250 |
| 2-5 Mb | 5048 | 0.3436 | 0.2622 | 0.2840 | 0.0414 | 0.0662 | 0.0190 |
| 5-10 Mb | 7756 | 0.2746 | 0.2272 | 0.2160 | 0.0256 | 0.0411 | 0.0110 |
| 10-20 Mb | 13101 | 0.2127 | 0.1838 | 0.1620 | 0.0156 | 0.0261 | 0.0060 |
| 20-50 | 30762 | 0.1411 | 0.1387 | 0.1000 | 0.0063 | 0.0105 | 0.0030 |
| > 50 Mb | 19662 | 0.1104 | 0.1147 | 0.0770 | 0.0038 | 0.0056 | 0.0020 |

BTA11

| Distance | N | Mean *D'* | SD *(D')* | Median *D'* | Mean *r^2^* | SD *(r^2^)* | Median *r^2^* |
| --- | --- | --- | --- | --- | --- | --- | --- |
| 0- 1 kb | 162 | 0.9834 | 0.0970 | 1.0000 | 0.7479 | 0.3396 | 0.9660 |
| 1-10 kb | 87 | 0.9633 | 0.1049 | 1.0000 | 0.5759 | 0.3622 | 0.6770 |
| 10-20 kb | 29 | 0.9354 | 0.1451 | 1.0000 | 0.4271 | 0.2895 | 0.3700 |
| 20-40 kb | 40 | 0.9003 | 0.2399 | 0.9975 | 0.4229 | 0.3946 | 0.2340 |
| 40-60 kb | 41 | 0.6494 | 0.3830 | 0.8540 | 0.1816 | 0.2141 | 0.1270 |
| 60-100 kb | 65 | 0.5512 | 0.3837 | 0.4230 | 0.1358 | 0.2047 | 0.0520 |
| 100-200 kb | 158 | 0.5662 | 0.3230 | 0.5750 | 0.0875 | 0.1212 | 0.0400 |
| 200-500 kb | 565 | 0.4463 | 0.2987 | 0.4110 | 0.0626 | 0.0882 | 0.0280 |
| 0.5-1 Mb | 924 | 0.4492 | 0.3122 | 0.4210 | 0.0611 | 0.0884 | 0.0300 |
| 1-2 Mb | 1889 | 0.4121 | 0.2723 | 0.3820 | 0.0527 | 0.0800 | 0.0250 |
| 2-5 Mb | 5675 | 0.3495 | 0.2642 | 0.2900 | 0.0381 | 0.0586 | 0.0170 |
| 5-10 Mb | 8752 | 0.2909 | 0.2382 | 0.2260 | 0.0238 | 0.0350 | 0.0110 |
| 10-20 Mb | 15576 | 0.2199 | 0.1938 | 0.1640 | 0.0133 | 0.0204 | 0.0060 |
| 20-50 | 39526 | 0.1368 | 0.1414 | 0.0930 | 0.0051 | 0.0081 | 0.0020 |
| > 50 Mb | 25746 | 0.1066 | 0.1142 | 0.0730 | 0.0029 | 0.0043 | 0.0010 |

BTA12

| Distance | N | Mean *D'* | SD *(D')* | Median *D'* | Mean *r^2^* | SD *(r^2^)* | Median *r^2^* |
| --- | --- | --- | --- | --- | --- | --- | --- |
| 0- 1 kb | 123 | 0.9978 | 0.0076 | 1.0000 | 0.8177 | 0.3154 | 0.9940 |
| 1-10 kb | 32 | 0.9889 | 0.0528 | 1.0000 | 0.6118 | 0.3405 | 0.6690 |
| 10-20 kb | 44 | 0.9579 | 0.0799 | 0.9940 | 0.6556 | 0.3774 | 0.8505 |
| 20-40 kb | 17 | 0.9600 | 0.0600 | 0.9850 | 0.4692 | 0.2775 | 0.5070 |
| 40-60 kb | 25 | 0.5638 | 0.4283 | 0.7400 | 0.2125 | 0.3082 | 0.0160 |
| 60-100 kb | 37 | 0.5358 | 0.3747 | 0.5590 | 0.0624 | 0.0773 | 0.0300 |
| 100-200 kb | 129 | 0.6120 | 0.3377 | 0.6690 | 0.1097 | 0.1334 | 0.0560 |
| 200-500 kb | 263 | 0.4719 | 0.3304 | 0.4020 | 0.0594 | 0.0884 | 0.0260 |
| 0.5-1 Mb | 518 | 0.3530 | 0.2920 | 0.2650 | 0.0386 | 0.0593 | 0.0150 |
| 1-2 Mb | 995 | 0.3674 | 0.2815 | 0.2950 | 0.0429 | 0.0627 | 0.0190 |
| 2-5 Mb | 2973 | 0.3031 | 0.2480 | 0.2400 | 0.0257 | 0.0384 | 0.0120 |
| 5-10 Mb | 4231 | 0.2606 | 0.2270 | 0.1900 | 0.0170 | 0.0239 | 0.0090 |
| 10-20 Mb | 7270 | 0.1996 | 0.1863 | 0.1430 | 0.0107 | 0.0156 | 0.0050 |
| 20-50 | 14937 | 0.1349 | 0.1395 | 0.0930 | 0.0046 | 0.0070 | 0.0020 |
| > 50 Mb | 6081 | 0.1100 | 0.1123 | 0.0740 | 0.0028 | 0.0040 | 0.0010 |

BTA13

| Distance | N | Mean *D'* | SD *(D')* | Median *D'* | Mean *r^2^* | SD *(r^2^)* | Median *r^2^* |
| --- | --- | --- | --- | --- | --- | --- | --- |
| 0- 1 kb | 209 | 0.9955 | 0.0526 | 1.0000 | 0.8044 | 0.3030 | 0.9960 |
| 1-10 kb | 78 | 0.9692 | 0.1298 | 1.0000 | 0.6512 | 0.3748 | 0.9005 |
| 10-20 kb | 32 | 0.8024 | 0.2786 | 0.9770 | 0.4117 | 0.3656 | 0.3280 |
| 20-40 kb | 42 | 0.8676 | 0.2475 | 1.0000 | 0.2435 | 0.2448 | 0.1390 |
| 40-60 kb | 38 | 0.7554 | 0.3233 | 0.9490 | 0.1859 | 0.1566 | 0.1410 |
| 60-100 kb | 44 | 0.6462 | 0.3340 | 0.6265 | 0.1416 | 0.1950 | 0.1120 |
| 100-200 kb | 189 | 0.5671 | 0.3393 | 0.6000 | 0.1296 | 0.1768 | 0.0480 |
| 200-500 kb | 503 | 0.4827 | 0.3226 | 0.4450 | 0.0825 | 0.1059 | 0.0450 |
| 0.5-1 Mb | 1011 | 0.4518 | 0.2966 | 0.4230 | 0.0722 | 0.0944 | 0.0380 |
| 1-2 Mb | 1969 | 0.3934 | 0.2856 | 0.3400 | 0.0536 | 0.0809 | 0.0230 |
| 2-5 Mb | 5604 | 0.3529 | 0.2704 | 0.2810 | 0.0407 | 0.0634 | 0.0170 |
| 5-10 Mb | 8599 | 0.2895 | 0.2334 | 0.2280 | 0.0252 | 0.0407 | 0.0100 |
| 10-20 Mb | 15184 | 0.2085 | 0.1830 | 0.1550 | 0.0128 | 0.0200 | 0.0060 |
| 20-50 | 33641 | 0.1313 | 0.1315 | 0.0930 | 0.0049 | 0.0074 | 0.0020 |
| > 50 Mb | 13458 | 0.1163 | 0.1263 | 0.0780 | 0.0033 | 0.0048 | 0.0020 |

BTA14

| Distance | N | Mean *D'* | SD *(D')* | Median *D'* | Mean *r^2^* | SD *(r^2^)* | Median *r^2^* |
| --- | --- | --- | --- | --- | --- | --- | --- |
| 0- 1 kb | 160 | 0.9912 | 0.0592 | 1.0000 | 0.7553 | 0.3571 | 0.9865 |
| 1-10 kb | 56 | 0.9565 | 0.1313 | 1.0000 | 0.6773 | 0.3526 | 0.7720 |
| 10-20 kb | 32 | 0.8162 | 0.3360 | 0.9970 | 0.3550 | 0.2903 | 0.3920 |
| 20-40 kb | 32 | 0.8124 | 0.2952 | 0.9680 | 0.4867 | 0.3728 | 0.5010 |
| 40-60 kb | 19 | 0.6817 | 0.4040 | 0.9540 | 0.1695 | 0.1784 | 0.1220 |
| 60-100 kb | 58 | 0.6086 | 0.3101 | 0.6410 | 0.1244 | 0.1643 | 0.0795 |
| 100-200 kb | 84 | 0.5838 | 0.3020 | 0.5605 | 0.1323 | 0.1299 | 0.0900 |
| 200-500 kb | 277 | 0.4937 | 0.3478 | 0.4980 | 0.0809 | 0.1228 | 0.0280 |
| 0.5-1 Mb | 482 | 0.4607 | 0.2953 | 0.4015 | 0.0806 | 0.1070 | 0.0480 |
| 1-2 Mb | 1204 | 0.4008 | 0.2738 | 0.3525 | 0.0609 | 0.0814 | 0.0300 |
| 2-5 Mb | 2796 | 0.3334 | 0.2698 | 0.2520 | 0.0393 | 0.0565 | 0.0150 |
| 5-10 Mb | 5259 | 0.2845 | 0.2259 | 0.2230 | 0.0288 | 0.0439 | 0.0120 |
| 10-20 Mb | 8191 | 0.2104 | 0.1822 | 0.1620 | 0.0143 | 0.0213 | 0.0070 |
| 20-50 | 17323 | 0.1413 | 0.1356 | 0.1030 | 0.0060 | 0.0090 | 0.0030 |
| > 50 Mb | 8578 | 0.1052 | 0.1065 | 0.0770 | 0.0037 | 0.0052 | 0.0020 |

BTA15

| Distance | N | Mean *D'* | SD *(D')* | Median *D'* | Mean *r^2^* | SD *(r^2^)* | Median *r^2^* |
| --- | --- | --- | --- | --- | --- | --- | --- |
| 0- 1 kb | 133 | 0.9871 | 0.0885 | 1.0000 | 0.7957 | 0.3240 | 0.9890 |
| 1-10 kb | 51 | 0.8848 | 0.2379 | 0.9980 | 0.5592 | 0.3895 | 0.6360 |
| 10-20 kb | 14 | 0.7664 | 0.3848 | 1.0000 | 0.1519 | 0.1933 | 0.1090 |
| 20-40 kb | 50 | 0.7695 | 0.3411 | 0.9780 | 0.4032 | 0.3767 | 0.2915 |
| 40-60 kb | 25 | 0.4422 | 0.2742 | 0.4820 | 0.1031 | 0.0896 | 0.0740 |
| 60-100 kb | 55 | 0.6943 | 0.2871 | 0.7030 | 0.2567 | 0.2792 | 0.2080 |
| 100-200 kb | 173 | 0.6139 | 0.3492 | 0.6870 | 0.1164 | 0.1397 | 0.0630 |
| 200-500 kb | 391 | 0.4799 | 0.3212 | 0.4030 | 0.0840 | 0.1141 | 0.0350 |
| 0.5-1 Mb | 712 | 0.3936 | 0.3021 | 0.2800 | 0.0394 | 0.0580 | 0.0205 |
| 1-2 Mb | 1254 | 0.3408 | 0.2687 | 0.2685 | 0.0384 | 0.0609 | 0.0155 |
| 2-5 Mb | 3491 | 0.3309 | 0.2728 | 0.2590 | 0.0286 | 0.0429 | 0.0120 |
| 5-10 Mb | 5926 | 0.2598 | 0.2188 | 0.2000 | 0.0189 | 0.0296 | 0.0080 |
| 10-20 Mb | 10857 | 0.2151 | 0.2002 | 0.1540 | 0.0115 | 0.0186 | 0.0050 |
| 20-50 | 19629 | 0.1486 | 0.1580 | 0.0990 | 0.0062 | 0.0168 | 0.0020 |
| > 50 Mb | 3295 | 0.1079 | 0.1135 | 0.0700 | 0.0033 | 0.0060 | 0.0010 |

BTA16

| Distance | N | Mean *D'* | SD *(D')* | Median *D'* | Mean *r^2^* | SD *(r^2^)* | Median *r^2^* |
| --- | --- | --- | --- | --- | --- | --- | --- |
| 0- 1 kb | 162 | 0.9939 | 0.0392 | 1.0000 | 0.8205 | 0.3209 | 0.9960 |
| 1-10 kb | 62 | 0.9292 | 0.1464 | 1.0000 | 0.5044 | 0.3863 | 0.2895 |
| 10-20 kb | 17 | 0.8874 | 0.2078 | 1.0000 | 0.4969 | 0.4323 | 0.4200 |
| 20-40 kb | 67 | 0.7283 | 0.2223 | 0.7610 | 0.2339 | 0.1652 | 0.1600 |
| 40-60 kb | 12 | 0.7462 | 0.3102 | 0.8835 | 0.4275 | 0.3625 | 0.3215 |
| 60-100 kb | 37 | 0.5904 | 0.3384 | 0.5810 | 0.2043 | 0.2336 | 0.1120 |
| 100-200 kb | 142 | 0.5075 | 0.3164 | 0.4940 | 0.1127 | 0.1447 | 0.0695 |
| 200-500 kb | 402 | 0.4233 | 0.2935 | 0.3805 | 0.0805 | 0.1021 | 0.0345 |
| 0.5-1 Mb | 699 | 0.4452 | 0.2844 | 0.4140 | 0.0690 | 0.0855 | 0.0400 |
| 1-2 Mb | 1124 | 0.3655 | 0.2628 | 0.2925 | 0.0479 | 0.0633 | 0.0250 |
| 2-5 Mb | 3859 | 0.3128 | 0.2384 | 0.2690 | 0.0388 | 0.0545 | 0.0180 |
| 5-10 Mb | 6280 | 0.2410 | 0.2051 | 0.1900 | 0.0220 | 0.0336 | 0.0090 |
| 10-20 Mb | 10743 | 0.1977 | 0.1712 | 0.1510 | 0.0139 | 0.0215 | 0.0060 |
| 20-50 | 21173 | 0.1378 | 0.1312 | 0.1000 | 0.0064 | 0.0107 | 0.0030 |
| > 50 Mb | 4676 | 0.1077 | 0.1157 | 0.0760 | 0.0035 | 0.0053 | 0.0010 |

BTA17

| Distance | N | Mean *D'* | SD *(D')* | Median *D'* | Mean *r^2^* | SD *(r^2^)* | Median *r^2^* |
| --- | --- | --- | --- | --- | --- | --- | --- |
| 0- 1 kb | 138 | 0.9845 | 0.0937 | 1.0000 | 0.7302 | 0.3432 | 0.9645 |
| 1-10 kb | 64 | 0.9738 | 0.1241 | 1.0000 | 0.6070 | 0.3726 | 0.5910 |
| 10-20 kb | 10 | 0.5277 | 0.3333 | 0.2915 | 0.1124 | 0.3114 | 0.0085 |
| 20-40 kb | 45 | 0.6604 | 0.3256 | 0.7300 | 0.3079 | 0.3480 | 0.1380 |
| 40-60 kb | 26 | 0.7394 | 0.1920 | 0.6875 | 0.2560 | 0.1941 | 0.2815 |
| 60-100 kb | 41 | 0.7146 | 0.3238 | 0.8850 | 0.1533 | 0.1990 | 0.0770 |
| 100-200 kb | 154 | 0.5849 | 0.2820 | 0.5310 | 0.1393 | 0.1946 | 0.0670 |
| 200-500 kb | 358 | 0.5147 | 0.3065 | 0.5390 | 0.0844 | 0.1133 | 0.0430 |
| 0.5-1 Mb | 780 | 0.4251 | 0.2865 | 0.3845 | 0.0599 | 0.0781 | 0.0260 |
| 1-2 Mb | 1357 | 0.3988 | 0.2765 | 0.3520 | 0.0471 | 0.0643 | 0.0210 |
| 2-5 Mb | 3766 | 0.3584 | 0.2640 | 0.3060 | 0.0379 | 0.0528 | 0.0200 |
| 5-10 Mb | 5793 | 0.2635 | 0.2155 | 0.2110 | 0.0201 | 0.0300 | 0.0090 |
| 10-20 Mb | 10342 | 0.1913 | 0.1685 | 0.1430 | 0.0110 | 0.0165 | 0.0050 |
| 20-50 | 18096 | 0.1197 | 0.1233 | 0.0820 | 0.0041 | 0.0064 | 0.0020 |
| > 50 Mb | 3581 | 0.0948 | 0.0977 | 0.0660 | 0.0025 | 0.0034 | 0.0010 |

BTA18

| Distance | N | Mean *D'* | SD *(D')* | Median *D'* | Mean *r^2^* | SD *(r^2^)* | Median *r^2^* |
| --- | --- | --- | --- | --- | --- | --- | --- |
| 0- 1 kb | 128 | 0.9593 | 0.1540 | 1.0000 | 0.7272 | 0.3625 | 0.9755 |
| 1-10 kb | 44 | 0.9528 | 0.1416 | 0.9980 | 0.4997 | 0.3769 | 0.4175 |
| 10-20 kb | 10 | 0.6031 | 0.4299 | 0.7630 | 0.1932 | 0.3148 | 0.0800 |
| 20-40 kb | 38 | 0.8761 | 0.2483 | 0.9960 | 0.2615 | 0.2129 | 0.2585 |
| 40-60 kb | 55 | 0.6218 | 0.3589 | 0.7090 | 0.2990 | 0.3729 | 0.1020 |
| 60-100 kb | 67 | 0.4967 | 0.3978 | 0.3690 | 0.1892 | 0.3019 | 0.0460 |
| 100-200 kb | 179 | 0.5975 | 0.3210 | 0.6560 | 0.1781 | 0.2533 | 0.0740 |
| 200-500 kb | 368 | 0.4181 | 0.2773 | 0.3475 | 0.0726 | 0.1120 | 0.0405 |
| 0.5-1 Mb | 580 | 0.3747 | 0.2871 | 0.3270 | 0.0475 | 0.0783 | 0.0160 |
| 1-2 Mb | 1429 | 0.3082 | 0.2476 | 0.2530 | 0.0295 | 0.0407 | 0.0160 |
| 2-5 Mb | 3756 | 0.2919 | 0.2515 | 0.2120 | 0.0246 | 0.0343 | 0.0120 |
| 5-10 Mb | 5868 | 0.2320 | 0.2007 | 0.1705 | 0.0166 | 0.0244 | 0.0080 |
| 10-20 Mb | 10668 | 0.1833 | 0.1770 | 0.1300 | 0.0090 | 0.0135 | 0.0040 |
| 20-50 | 17696 | 0.1164 | 0.1260 | 0.0780 | 0.0039 | 0.0059 | 0.0020 |
| > 50 Mb | 1309 | 0.1240 | 0.1362 | 0.0770 | 0.0032 | 0.0045 | 0.0010 |

BTA19

| Distance | N | Mean *D'* | SD *(D')* | Median *D'* | Mean *r^2^* | SD *(r^2^)* | Median *r^2^* |
| --- | --- | --- | --- | --- | --- | --- | --- |
| 0- 1 kb | 128 | 0.9832 | 0.0927 | 1.0000 | 0.6748 | 0.3454 | 0.7670 |
| 1-10 kb | 95 | 0.9514 | 0.1493 | 0.9980 | 0.5578 | 0.3549 | 0.5630 |
| 10-20 kb | 30 | 0.8592 | 0.2177 | 0.9910 | 0.3860 | 0.3899 | 0.2330 |
| 20-40 kb | 65 | 0.7110 | 0.3119 | 0.8140 | 0.1662 | 0.2344 | 0.0650 |
| 40-60 kb | 90 | 0.6234 | 0.2780 | 0.6480 | 0.1957 | 0.1825 | 0.1535 |
| 60-100 kb | 103 | 0.5080 | 0.3637 | 0.3840 | 0.0903 | 0.1223 | 0.0150 |
| 100-200 kb | 231 | 0.5660 | 0.3128 | 0.5720 | 0.0987 | 0.1270 | 0.0610 |
| 200-500 kb | 808 | 0.4370 | 0.3016 | 0.3885 | 0.0616 | 0.0959 | 0.0300 |
| 0.5-1 Mb | 943 | 0.3864 | 0.2943 | 0.3080 | 0.0447 | 0.0648 | 0.0180 |
| 1-2 Mb | 1991 | 0.3713 | 0.2767 | 0.3150 | 0.0409 | 0.0570 | 0.0190 |
| 2-5 Mb | 5791 | 0.3155 | 0.2461 | 0.2560 | 0.0296 | 0.0447 | 0.0140 |
| 5-10 Mb | 7547 | 0.2504 | 0.2075 | 0.1930 | 0.0181 | 0.0267 | 0.0090 |
| 10-20 Mb | 14185 | 0.1727 | 0.1602 | 0.1250 | 0.0089 | 0.0135 | 0.0040 |
| 20-50 | 23674 | 0.1136 | 0.1184 | 0.0780 | 0.0036 | 0.0054 | 0.0020 |
| > 50 Mb | 1610 | 0.0854 | 0.0965 | 0.0590 | 0.0024 | 0.0036 | 0.0010 |

BTA20

| Distance | N | Mean *D'* | SD *(D')* | Median *D'* | Mean *r^2^* | SD *(r^2^)* | Median *r^2^* |
| --- | --- | --- | --- | --- | --- | --- | --- |
| 0- 1 kb | 117 | 0.9850 | 0.0745 | 1.0000 | 0.8161 | 0.2967 | 0.9950 |
| 1-10 kb | 24 | 0.9053 | 0.2132 | 1.0000 | 0.5099 | 0.3314 | 0.4040 |
| 10-20 kb | 8 | 0.6471 | 0.3878 | 0.7165 | 0.1766 | 0.3401 | 0.0380 |
| 20-40 kb | 27 | 0.7704 | 0.3440 | 0.9570 | 0.2027 | 0.1956 | 0.1640 |
| 40-60 kb | 12 | 0.8696 | 0.2280 | 0.9945 | 0.3127 | 0.2905 | 0.1770 |
| 60-100 kb | 27 | 0.6727 | 0.3722 | 0.9060 | 0.1620 | 0.2265 | 0.0780 |
| 100-200 kb | 83 | 0.5092 | 0.3051 | 0.4900 | 0.1356 | 0.1791 | 0.0320 |
| 200-500 kb | 247 | 0.4606 | 0.2954 | 0.4450 | 0.1018 | 0.1503 | 0.0380 |
| 0.5-1 Mb | 476 | 0.3711 | 0.2763 | 0.3010 | 0.0473 | 0.0670 | 0.0180 |
| 1-2 Mb | 815 | 0.3943 | 0.2767 | 0.3340 | 0.0492 | 0.0711 | 0.0250 |
| 2-5 Mb | 2581 | 0.3586 | 0.2591 | 0.3110 | 0.0419 | 0.0632 | 0.0170 |
| 5-10 Mb | 3676 | 0.2867 | 0.2251 | 0.2270 | 0.0288 | 0.0451 | 0.0120 |
| 10-20 Mb | 6663 | 0.2181 | 0.1862 | 0.1660 | 0.0151 | 0.0222 | 0.0070 |
| 20-50 | 13342 | 0.1482 | 0.1470 | 0.1040 | 0.0070 | 0.0141 | 0.0030 |
| > 50 Mb | 2778 | 0.1178 | 0.1233 | 0.0760 | 0.0041 | 0.0091 | 0.0020 |

BTA21

| Distance | N | Mean *D'* | SD *(D')* | Median *D'* | Mean *r^2^* | SD *(r^2^)* | Median *r^2^* |
| --- | --- | --- | --- | --- | --- | --- | --- |
| 0- 1 kb | 54 | 0.9659 | 0.1323 | 1.0000 | 0.6854 | 0.3638 | 0.9625 |
| 1-10 kb | 33 | 0.9358 | 0.2011 | 0.9980 | 0.5122 | 0.3537 | 0.4480 |
| 10-20 kb | 1 | 1.0000 | NA | 1.0000 | 0.0480 | NA | 0.0480 |
| 20-40 kb | 47 | 0.9781 | 0.1372 | 1.0000 | 0.5492 | 0.3303 | 0.4470 |
| 40-60 kb | 8 | 0.7450 | 0.2714 | 0.8240 | 0.2023 | 0.2373 | 0.1240 |
| 60-100 kb | 18 | 0.5934 | 0.3519 | 0.6615 | 0.0701 | 0.0756 | 0.0470 |
| 100-200 kb | 35 | 0.6616 | 0.3093 | 0.6820 | 0.1198 | 0.1558 | 0.0650 |
| 200-500 kb | 208 | 0.4312 | 0.3201 | 0.3530 | 0.0580 | 0.0807 | 0.0290 |
| 0.5-1 Mb | 272 | 0.4149 | 0.2947 | 0.3875 | 0.0524 | 0.0654 | 0.0260 |
| 1-2 Mb | 526 | 0.3606 | 0.2688 | 0.3125 | 0.0459 | 0.0590 | 0.0275 |
| 2-5 Mb | 1584 | 0.3114 | 0.2541 | 0.2420 | 0.0271 | 0.0372 | 0.0120 |
| 5-10 Mb | 2429 | 0.2455 | 0.2155 | 0.1790 | 0.0174 | 0.0242 | 0.0080 |
| 10-20 Mb | 4204 | 0.1955 | 0.1829 | 0.1400 | 0.0110 | 0.0192 | 0.0050 |
| 20-50 | 6556 | 0.1337 | 0.1448 | 0.0880 | 0.0061 | 0.0155 | 0.0020 |
| > 50 Mb | 315 | 0.0982 | 0.1112 | 0.0610 | 0.0036 | 0.0080 | 0.0010 |

BTA22

| Distance | N | Mean *D'* | SD *(D')* | Median *D'* | Mean *r^2^* | SD *(r^2^)* | Median *r^2^* |
| --- | --- | --- | --- | --- | --- | --- | --- |
| 0- 1 kb | 111 | 0.9930 | 0.0522 | 1.0000 | 0.8337 | 0.2982 | 0.9980 |
| 1-10 kb | 81 | 0.9914 | 0.0420 | 1.0000 | 0.5828 | 0.3019 | 0.6160 |
| 10-20 kb | 10 | 0.8410 | 0.2752 | 0.9905 | 0.4500 | 0.3970 | 0.3645 |
| 20-40 kb | 27 | 0.6310 | 0.3919 | 0.7620 | 0.2226 | 0.2443 | 0.0920 |
| 40-60 kb | 27 | 0.7270 | 0.2836 | 0.7210 | 0.1365 | 0.1855 | 0.0730 |
| 60-100 kb | 51 | 0.5651 | 0.2930 | 0.5770 | 0.1273 | 0.1947 | 0.0700 |
| 100-200 kb | 104 | 0.4802 | 0.2946 | 0.3975 | 0.0915 | 0.1096 | 0.0470 |
| 200-500 kb | 304 | 0.5612 | 0.3104 | 0.5910 | 0.1062 | 0.1276 | 0.0580 |
| 0.5-1 Mb | 520 | 0.4290 | 0.2866 | 0.3885 | 0.0707 | 0.1023 | 0.0280 |
| 1-2 Mb | 1086 | 0.3927 | 0.2826 | 0.3400 | 0.0562 | 0.0855 | 0.0210 |
| 2-5 Mb | 2939 | 0.3561 | 0.2627 | 0.2930 | 0.0412 | 0.0616 | 0.0190 |
| 5-10 Mb | 4633 | 0.2661 | 0.2100 | 0.2140 | 0.0242 | 0.0355 | 0.0110 |
| 10-20 Mb | 8157 | 0.1936 | 0.1650 | 0.1450 | 0.0120 | 0.0174 | 0.0050 |
| 20-50 | 12488 | 0.1185 | 0.1190 | 0.0840 | 0.0043 | 0.0065 | 0.0020 |
| > 50 Mb | 338 | 0.0969 | 0.1026 | 0.0695 | 0.0025 | 0.0036 | 0.0010 |

BTA23

| Distance | N | Mean *D'* | SD *(D')* | Median *D'* | Mean *r^2^* | SD *(r^2^)* | Median *r^2^* |
| --- | --- | --- | --- | --- | --- | --- | --- |
| 0- 1 kb | 108 | 0.9911 | 0.0620 | 1.0000 | 0.7344 | 0.3537 | 0.9770 |
| 1-10 kb | 47 | 0.9649 | 0.1255 | 1.0000 | 0.5066 | 0.4110 | 0.3090 |
| 10-20 kb | 16 | 0.7983 | 0.2555 | 0.9285 | 0.2983 | 0.2450 | 0.3035 |
| 20-40 kb | 63 | 0.7687 | 0.2786 | 0.8850 | 0.1914 | 0.2443 | 0.0830 |
| 40-60 kb | 26 | 0.5871 | 0.3159 | 0.6180 | 0.1338 | 0.1871 | 0.0685 |
| 60-100 kb | 77 | 0.4941 | 0.3655 | 0.4420 | 0.1177 | 0.1967 | 0.0310 |
| 100-200 kb | 264 | 0.5454 | 0.3230 | 0.5320 | 0.1035 | 0.1596 | 0.0410 |
| 200-500 kb | 466 | 0.4568 | 0.3207 | 0.3845 | 0.0898 | 0.1576 | 0.0320 |
| 0.5-1 Mb | 557 | 0.4509 | 0.3181 | 0.3980 | 0.0588 | 0.0887 | 0.0280 |
| 1-2 Mb | 1333 | 0.4481 | 0.3048 | 0.4050 | 0.0562 | 0.0921 | 0.0270 |
| 2-5 Mb | 4190 | 0.3732 | 0.2813 | 0.3090 | 0.0409 | 0.0626 | 0.0200 |
| 5-10 Mb | 5902 | 0.3024 | 0.2363 | 0.2420 | 0.0264 | 0.0409 | 0.0130 |
| 10-20 Mb | 10216 | 0.1941 | 0.1710 | 0.1440 | 0.0111 | 0.0173 | 0.0050 |
| 20-50 | 9888 | 0.1328 | 0.1295 | 0.0940 | 0.0057 | 0.0089 | 0.0020 |
| > 50 Mb | 0 | NA | NA | NA | NA | NA | NA |

BTA24

| Distance | N | Mean *D'* | SD *(D')* | Median *D'* | Mean *r^2^* | SD *(r^2^)* | Median *r^2^* |
| --- | --- | --- | --- | --- | --- | --- | --- |
| 0- 1 kb | 103 | 0.9750 | 0.1183 | 1.0000 | 0.7407 | 0.3516 | 0.9460 |
| 1-10 kb | 32 | 0.9593 | 0.1529 | 1.0000 | 0.6178 | 0.3990 | 0.8205 |
| 10-20 kb | 6 | 0.9812 | 0.0170 | 0.9805 | 0.6982 | 0.3309 | 0.7865 |
| 20-40 kb | 24 | 0.7612 | 0.3850 | 0.9655 | 0.6107 | 0.3739 | 0.7445 |
| 40-60 kb | 14 | 0.6420 | 0.3266 | 0.6875 | 0.1129 | 0.1113 | 0.0805 |
| 60-100 kb | 27 | 0.6244 | 0.3977 | 0.6710 | 0.1409 | 0.1671 | 0.0910 |
| 100-200 kb | 86 | 0.5029 | 0.3207 | 0.4175 | 0.0964 | 0.1286 | 0.0475 |
| 200-500 kb | 269 | 0.4551 | 0.3065 | 0.4090 | 0.0593 | 0.0726 | 0.0330 |
| 0.5-1 Mb | 398 | 0.3279 | 0.2584 | 0.2380 | 0.0293 | 0.0442 | 0.0140 |
| 1-2 Mb | 750 | 0.3966 | 0.2863 | 0.3405 | 0.0462 | 0.0624 | 0.0230 |
| 2-5 Mb | 2339 | 0.3069 | 0.2455 | 0.2430 | 0.0308 | 0.0474 | 0.0140 |
| 5-10 Mb | 3665 | 0.2455 | 0.2081 | 0.1890 | 0.0198 | 0.0305 | 0.0080 |
| 10-20 Mb | 6632 | 0.1835 | 0.1626 | 0.1400 | 0.0107 | 0.0152 | 0.0050 |
| 20-50 | 8911 | 0.1352 | 0.1359 | 0.0930 | 0.0054 | 0.0084 | 0.0020 |
| > 50 Mb | 397 | 0.0952 | 0.1024 | 0.0680 | 0.0029 | 0.0047 | 0.0010 |

BTA25

| Distance | N | Mean *D'* | SD *(D')* | Median *D'* | Mean *r^2^* | SD *(r^2^)* | Median *r^2^* |
| --- | --- | --- | --- | --- | --- | --- | --- |
| 0- 1 kb | 82 | 0.9671 | 0.1632 | 1.0000 | 0.7367 | 0.3425 | 0.9705 |
| 1-10 kb | 30 | 0.9887 | 0.0235 | 1.0000 | 0.4949 | 0.3799 | 0.3570 |
| 10-20 kb | 19 | 0.8149 | 0.3121 | 0.9940 | 0.5622 | 0.4304 | 0.7150 |
| 20-40 kb | 36 | 0.7069 | 0.3298 | 0.8195 | 0.1948 | 0.2115 | 0.1025 |
| 40-60 kb | 32 | 0.5352 | 0.3429 | 0.5745 | 0.1913 | 0.1867 | 0.1885 |
| 60-100 kb | 43 | 0.5929 | 0.3697 | 0.5770 | 0.1588 | 0.2493 | 0.0620 |
| 100-200 kb | 109 | 0.4534 | 0.2984 | 0.4590 | 0.0771 | 0.1220 | 0.0420 |
| 200-500 kb | 343 | 0.4103 | 0.2792 | 0.3660 | 0.0685 | 0.0837 | 0.0340 |
| 0.5-1 Mb | 544 | 0.3531 | 0.2583 | 0.2995 | 0.0507 | 0.0660 | 0.0240 |
| 1-2 Mb | 1082 | 0.3224 | 0.2661 | 0.2500 | 0.0356 | 0.0465 | 0.0170 |
| 2-5 Mb | 3326 | 0.2632 | 0.2226 | 0.2090 | 0.0274 | 0.0476 | 0.0120 |
| 5-10 Mb | 4707 | 0.2071 | 0.1894 | 0.1510 | 0.0175 | 0.0332 | 0.0060 |
| 10-20 Mb | 8072 | 0.1441 | 0.1360 | 0.1050 | 0.0077 | 0.0119 | 0.0030 |
| 20-50 | 6328 | 0.1073 | 0.1100 | 0.0770 | 0.0036 | 0.0052 | 0.0020 |
| > 50 Mb | 0 | NA | NA | NA | NA | NA | NA |

BTA26

| Distance | N | Mean *D'* | SD *(D')* | Median *D'* | Mean *r^2^* | SD *(r^2^)* | Median *r^2^* |
| --- | --- | --- | --- | --- | --- | --- | --- |
| 0- 1 kb | 62 | 0.9693 | 0.1079 | 1.0000 | 0.6428 | 0.3863 | 0.8800 |
| 1-10 kb | 32 | 0.9250 | 0.2053 | 1.0000 | 0.6305 | 0.3776 | 0.8510 |
| 10-20 kb | 7 | 0.9527 | 0.1233 | 1.0000 | 0.4749 | 0.4812 | 0.1650 |
| 20-40 kb | 18 | 0.5899 | 0.3177 | 0.7085 | 0.1884 | 0.2329 | 0.0545 |
| 40-60 kb | 5 | 0.8622 | 0.1258 | 0.7710 | 0.3186 | 0.2534 | 0.5010 |
| 60-100 kb | 63 | 0.7055 | 0.3257 | 0.8290 | 0.1180 | 0.1654 | 0.0670 |
| 100-200 kb | 80 | 0.4567 | 0.2954 | 0.4075 | 0.0910 | 0.1358 | 0.0430 |
| 200-500 kb | 236 | 0.5148 | 0.3178 | 0.4750 | 0.0732 | 0.0912 | 0.0410 |
| 0.5-1 Mb | 325 | 0.4463 | 0.3045 | 0.3890 | 0.0505 | 0.0809 | 0.0210 |
| 1-2 Mb | 662 | 0.3857 | 0.2871 | 0.3290 | 0.0465 | 0.0629 | 0.0220 |
| 2-5 Mb | 1624 | 0.3391 | 0.2633 | 0.2725 | 0.0337 | 0.0537 | 0.0130 |
| 5-10 Mb | 3099 | 0.2821 | 0.2278 | 0.2220 | 0.0215 | 0.0318 | 0.0100 |
| 10-20 Mb | 4432 | 0.2236 | 0.1935 | 0.1650 | 0.0144 | 0.0215 | 0.0065 |
| 20-50 | 5826 | 0.1507 | 0.1487 | 0.1060 | 0.0059 | 0.0088 | 0.0020 |
| > 50 Mb | 0 | NA | NA | NA | NA | NA | NA |

BTA27

| Distance | N | Mean *D'* | SD *(D')* | Median *D'* | Mean *r^2^* | SD *(r^2^)* | Median *r^2^* |
| --- | --- | --- | --- | --- | --- | --- | --- |
| 0- 1 kb | 68 | 0.9959 | 0.0133 | 1.0000 | 0.7742 | 0.3257 | 0.9675 |
| 1-10 kb | 17 | 0.9467 | 0.1920 | 1.0000 | 0.7440 | 0.3352 | 0.9580 |
| 10-20 kb | 5 | 0.8354 | 0.1544 | 0.7820 | 0.2096 | 0.1433 | 0.2060 |
| 20-40 kb | 21 | 0.7221 | 0.2172 | 0.7140 | 0.2132 | 0.1814 | 0.1770 |
| 40-60 kb | 31 | 0.6763 | 0.2964 | 0.7460 | 0.1909 | 0.2506 | 0.0700 |
| 60-100 kb | 32 | 0.5951 | 0.3528 | 0.6810 | 0.2047 | 0.2164 | 0.1410 |
| 100-200 kb | 68 | 0.4142 | 0.2874 | 0.3250 | 0.0606 | 0.0968 | 0.0450 |
| 200-500 kb | 167 | 0.3793 | 0.3023 | 0.3090 | 0.0456 | 0.0794 | 0.0160 |
| 0.5-1 Mb | 231 | 0.3129 | 0.2557 | 0.2320 | 0.0365 | 0.0663 | 0.0180 |
| 1-2 Mb | 521 | 0.2902 | 0.2384 | 0.2240 | 0.0358 | 0.0469 | 0.0170 |
| 2-5 Mb | 1655 | 0.2482 | 0.2150 | 0.1890 | 0.0230 | 0.0339 | 0.0100 |
| 5-10 Mb | 2512 | 0.2099 | 0.1883 | 0.1555 | 0.0165 | 0.0252 | 0.0070 |
| 10-20 Mb | 3742 | 0.1535 | 0.1430 | 0.1170 | 0.0087 | 0.0126 | 0.0040 |
| 20-50 | 3020 | 0.1106 | 0.1063 | 0.0820 | 0.0044 | 0.0063 | 0.0020 |
| > 50 Mb | 0 | NA | NA | NA | NA | NA | NA |

BTA28

| Distance | N | Mean *D'* | SD *(D')* | Median *D'* | Mean *r^2^* | SD *(r^2^)* | Median *r^2^* |
| --- | --- | --- | --- | --- | --- | --- | --- |
| 0- 1 kb | 74 | 0.9929 | 0.0264 | 1.0000 | 0.7216 | 0.3720 | 0.9785 |
| 1-10 kb | 13 | 0.8704 | 0.2335 | 0.9980 | 0.4725 | 0.3733 | 0.4550 |
| 10-20 kb | 10 | 0.8639 | 0.1987 | 0.9705 | 0.2852 | 0.2870 | 0.1345 |
| 20-40 kb | 11 | 0.7918 | 0.1985 | 0.8510 | 0.1591 | 0.2155 | 0.0560 |
| 40-60 kb | 8 | 0.5751 | 0.3836 | 0.5480 | 0.1445 | 0.2397 | 0.0405 |
| 60-100 kb | 26 | 0.5911 | 0.3094 | 0.6245 | 0.1166 | 0.1347 | 0.0515 |
| 100-200 kb | 58 | 0.4951 | 0.3376 | 0.4840 | 0.0574 | 0.0581 | 0.0340 |
| 200-500 kb | 205 | 0.4059 | 0.3022 | 0.3450 | 0.0512 | 0.0640 | 0.0300 |
| 0.5-1 Mb | 275 | 0.4167 | 0.2815 | 0.3510 | 0.0488 | 0.0727 | 0.0320 |
| 1-2 Mb | 591 | 0.3525 | 0.2697 | 0.2820 | 0.0423 | 0.0566 | 0.0200 |
| 2-5 Mb | 1613 | 0.3015 | 0.2290 | 0.2540 | 0.0264 | 0.0353 | 0.0140 |
| 5-10 Mb | 2318 | 0.2520 | 0.2084 | 0.1980 | 0.0190 | 0.0264 | 0.0090 |
| 10-20 Mb | 3455 | 0.1755 | 0.1634 | 0.1260 | 0.0082 | 0.0114 | 0.0040 |
| 20-50 | 3746 | 0.1210 | 0.1209 | 0.0850 | 0.0046 | 0.0071 | 0.0020 |
| > 50 Mb | 0 | NA | NA | NA | NA | NA | NA |

BTA29

| Distance | N | Mean *D'* | SD *(D')* | Median *D'* | Mean *r^2^* | SD *(r^2^)* | Median *r^2^* |
| --- | --- | --- | --- | --- | --- | --- | --- |
| 0- 1 kb | 73 | 0.9975 | 0.0146 | 1.0000 | 0.8220 | 0.3126 | 0.9960 |
| 1-10 kb | 51 | 0.9951 | 0.0184 | 1.0000 | 0.8228 | 0.3005 | 0.9930 |
| 10-20 kb | 11 | 0.9786 | 0.0428 | 0.9970 | 0.5311 | 0.4142 | 0.3630 |
| 20-40 kb | 14 | 0.6924 | 0.3504 | 0.8565 | 0.1956 | 0.2802 | 0.0450 |
| 40-60 kb | 10 | 0.8274 | 0.2469 | 0.8840 | 0.2601 | 0.2440 | 0.1730 |
| 60-100 kb | 42 | 0.4259 | 0.2415 | 0.3925 | 0.0648 | 0.0597 | 0.0525 |
| 100-200 kb | 71 | 0.5303 | 0.3271 | 0.4260 | 0.0901 | 0.0974 | 0.0560 |
| 200-500 kb | 188 | 0.4672 | 0.3155 | 0.3935 | 0.0750 | 0.0991 | 0.0295 |
| 0.5-1 Mb | 293 | 0.4056 | 0.2599 | 0.3570 | 0.0534 | 0.0571 | 0.0330 |
| 1-2 Mb | 718 | 0.3909 | 0.2833 | 0.3395 | 0.0502 | 0.0747 | 0.0280 |
| 2-5 Mb | 1740 | 0.3180 | 0.2505 | 0.2750 | 0.0371 | 0.0589 | 0.0140 |
| 5-10 Mb | 2835 | 0.2312 | 0.2022 | 0.1760 | 0.0197 | 0.0344 | 0.0080 |
| 10-20 Mb | 4025 | 0.1630 | 0.1530 | 0.1200 | 0.0085 | 0.0122 | 0.0040 |
| 20-50 | 5329 | 0.1176 | 0.1218 | 0.0820 | 0.0037 | 0.0055 | 0.0020 |
| > 50 Mb | 0 | NA | NA | NA | NA | NA | NA |
