# Supplementary material for: Extent of genome-wide linkage disequilibrium in Australian Holstein-Friesian cattle based on a high-density SNP panel
Source: BMC Genomics. 2008 Apr 24;9:187. doi: 10.1186/1471-2164-9-187 (PMC2386485; doi:10.1186/1471-2164-9-187)

**Figure S9 :** Distribution of *D′* between SNP pairs in relation to the physical distance between loci (Mb), for individual autosomes (1-29). The red line shows average *D'* in each 500 kb sliding window. The blue line shows the theoretical distribution from the Malécot model.


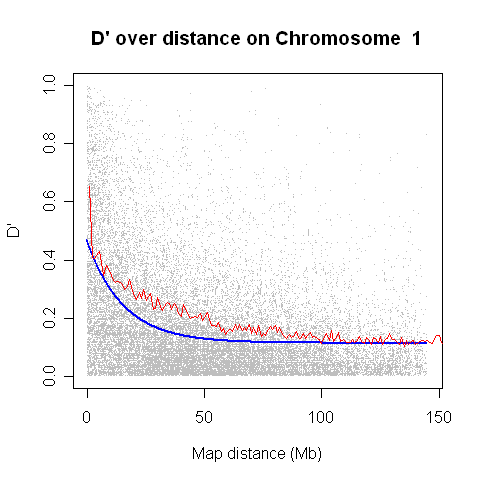


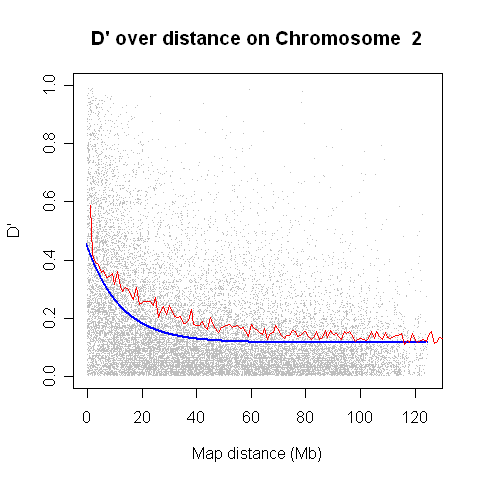

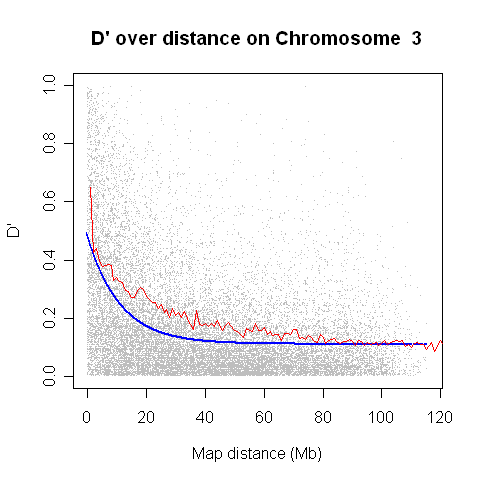

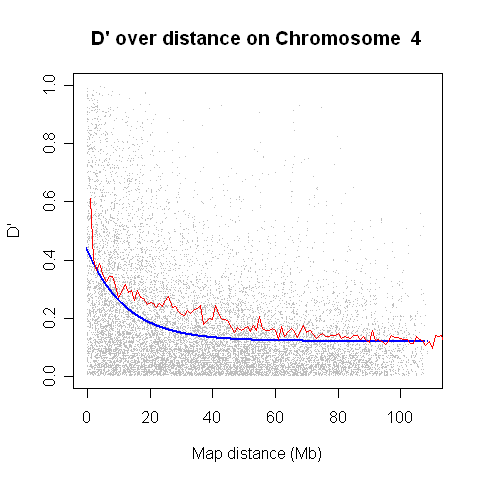

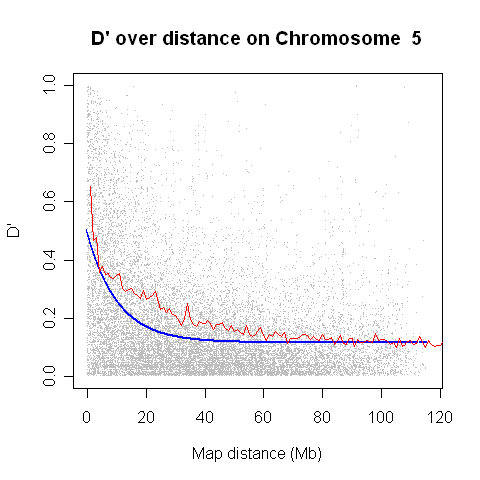

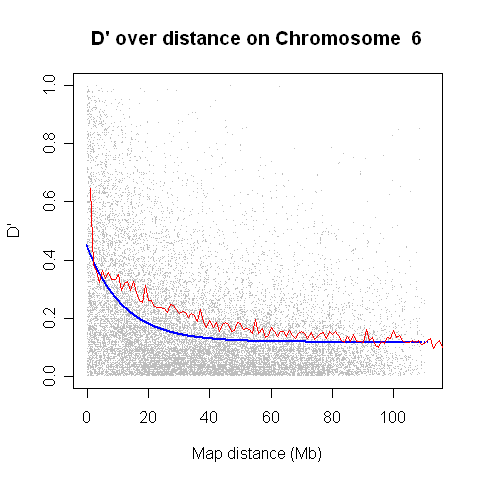

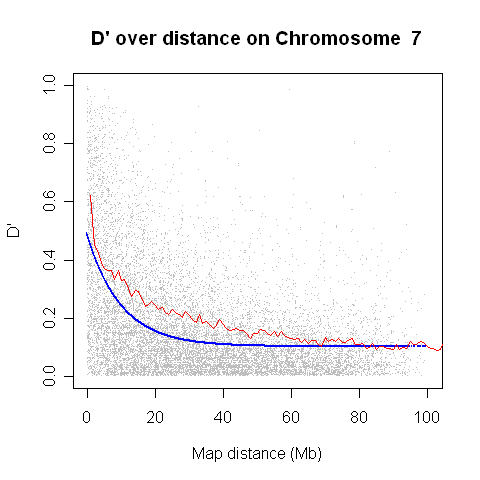

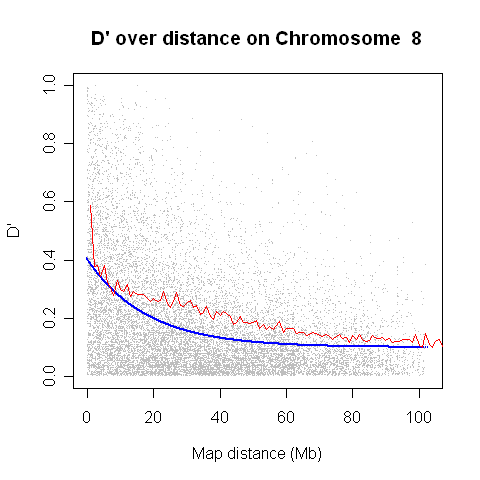

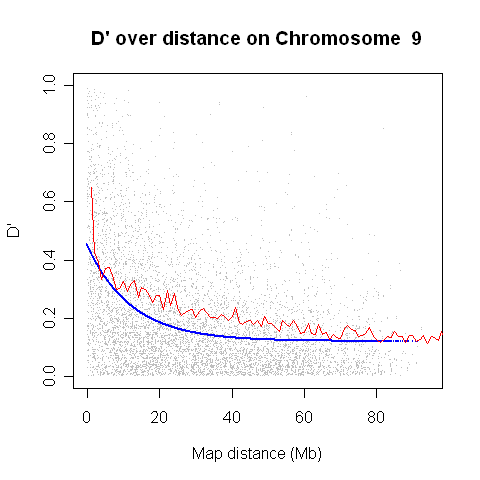

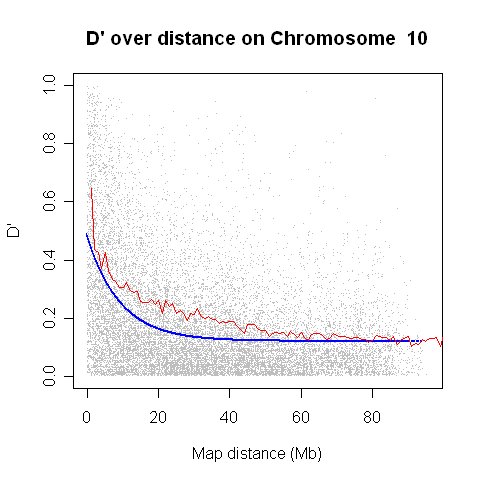

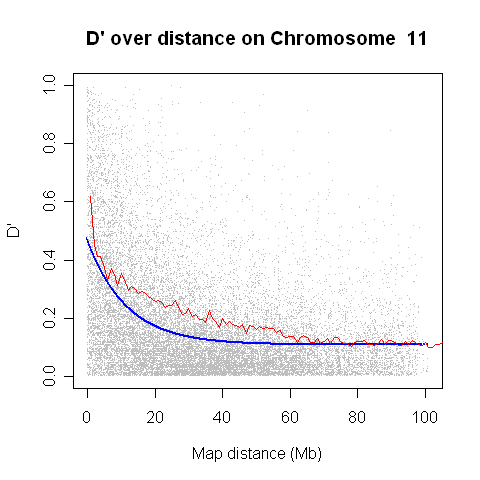

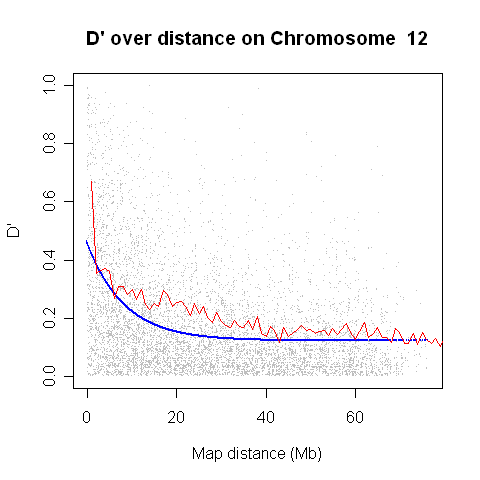

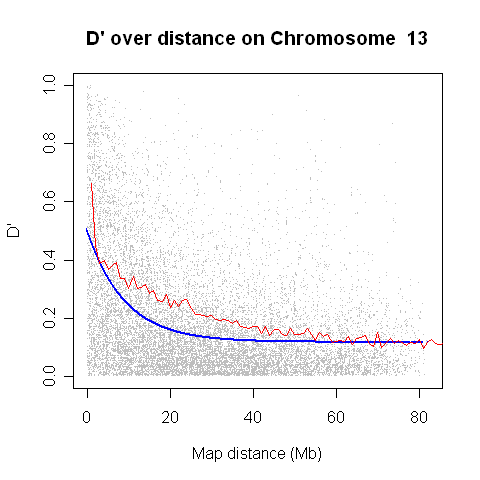

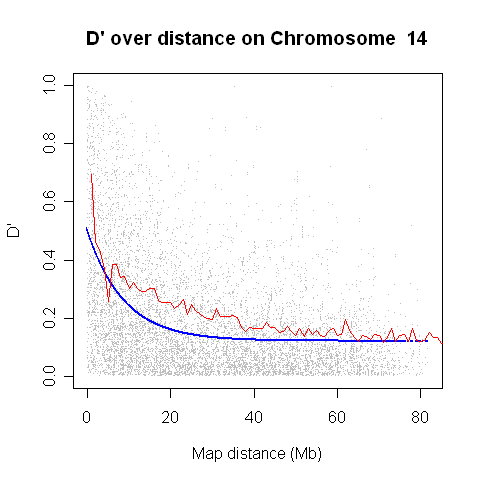

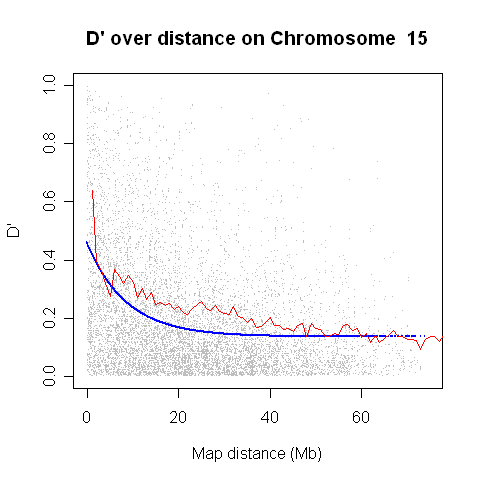

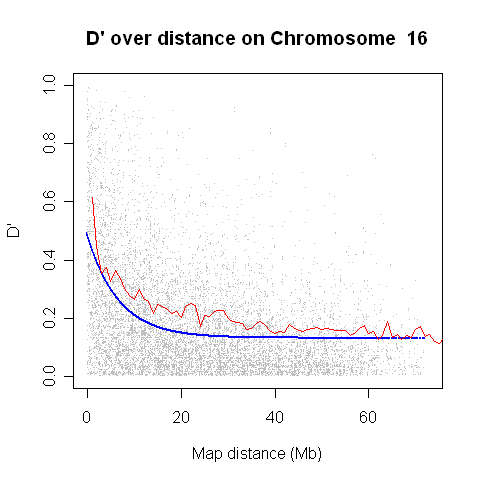

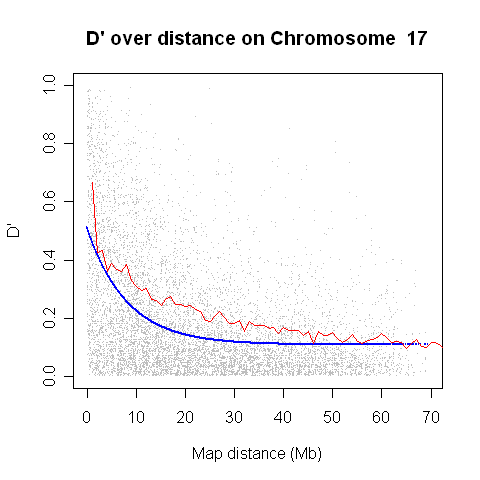

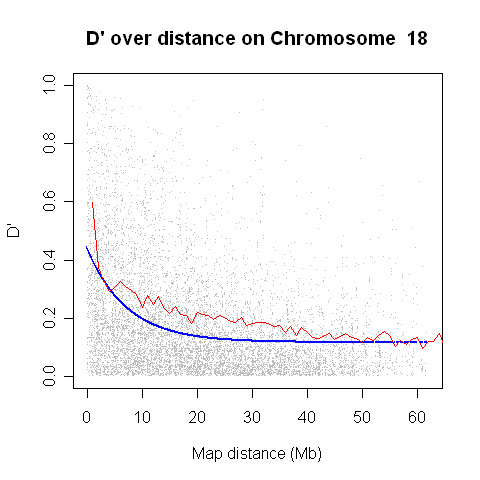

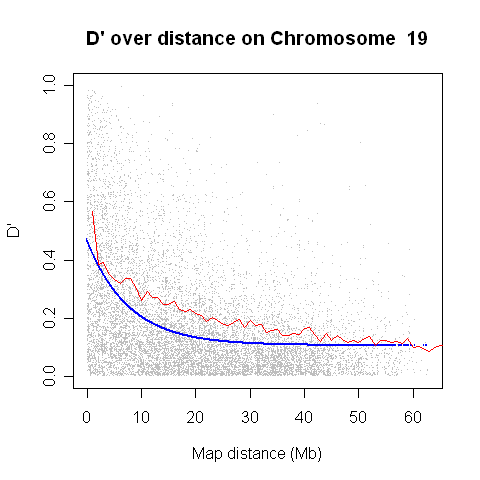

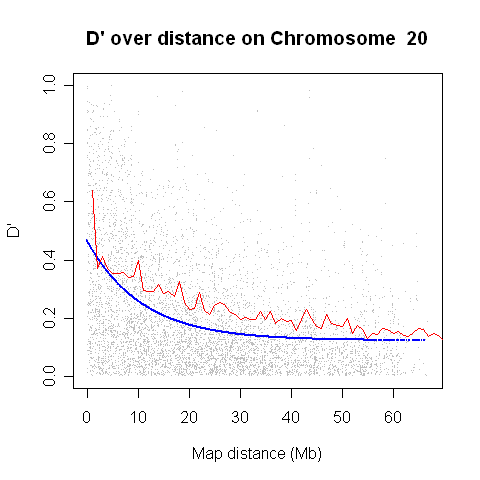

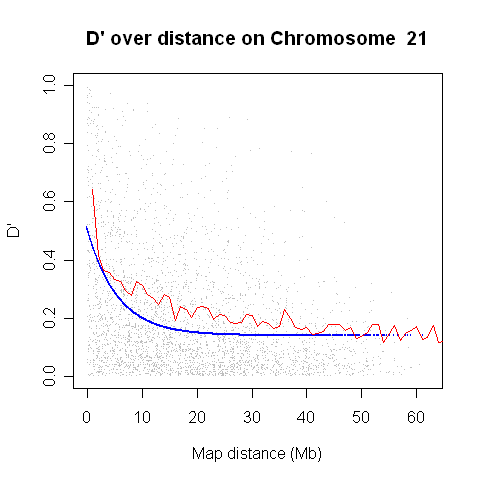

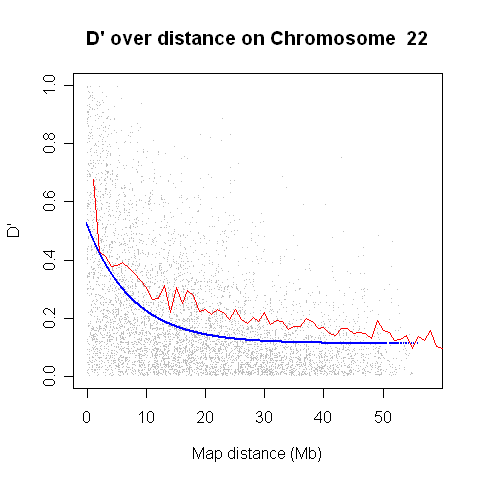

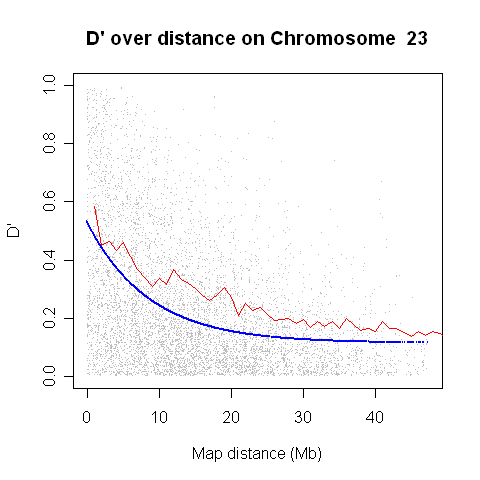

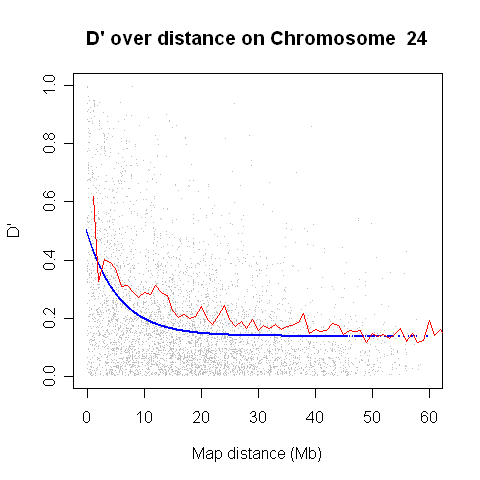

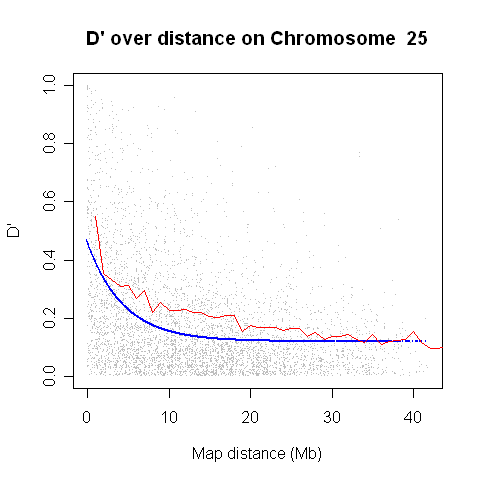


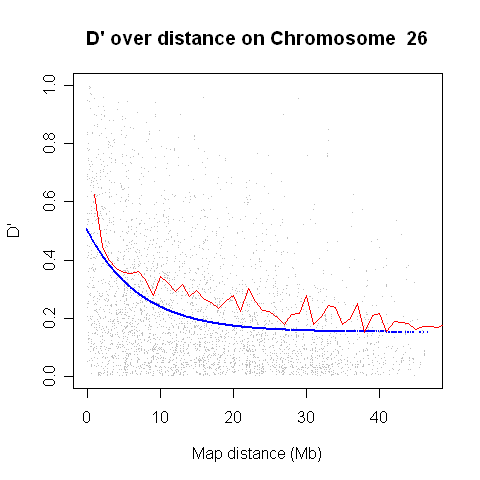


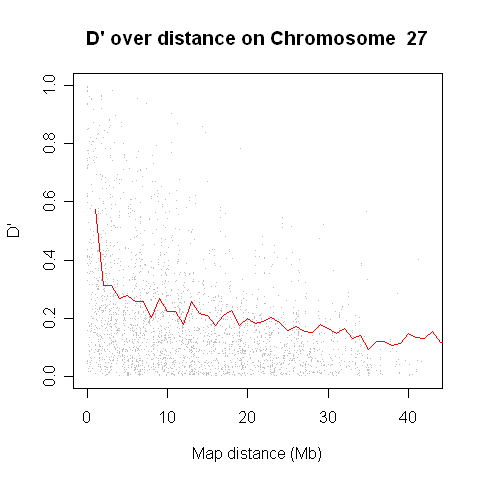


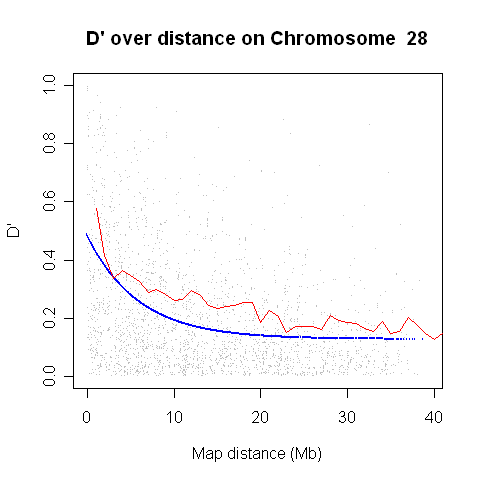

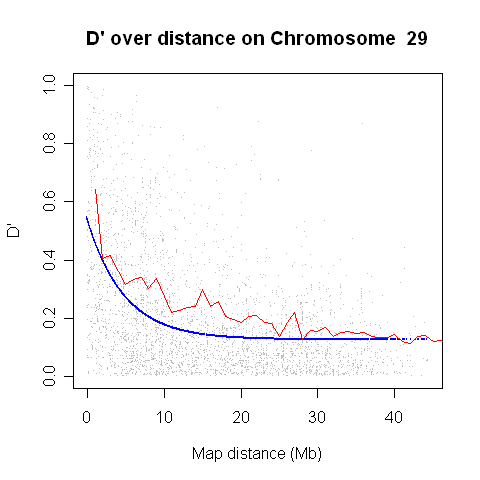

Supplement: Additional file 4 — Figure S9. Distribution of D' between SNP pairs in relation to the physical distance between loci (Mb), for individual autosomes (1–29). The red line shows average D' in each 500 kb sliding window. The blue line shows the theoretical distribution from the Malécot model. [file 1471-2164-9-187-S4.doc]
