# Supplementary material for: Extent of genome-wide linkage disequilibrium in Australian Holstein-Friesian cattle based on a high-density SNP panel
Source: BMC Genomics. 2008 Apr 24;9:187. doi: 10.1186/1471-2164-9-187 (PMC2386485; doi:10.1186/1471-2164-9-187)
Supplement: Additional file 5 — Figure S10. Distribution of r2 between SNP pairs in relation to the physical distance (Mb) between loci, for individual autosomes (1–29). The red line shows average r2 in each 500 kb sliding window. [file 1471-2164-9-187-S5.doc]

**Figure S10:** Distribution of *r^2^* between SNP pairs in relation to the physical distance (Mb) between loci, for individual autosomes (1-29). The red line shows average *r^2^* in each 500 kb sliding window.

**
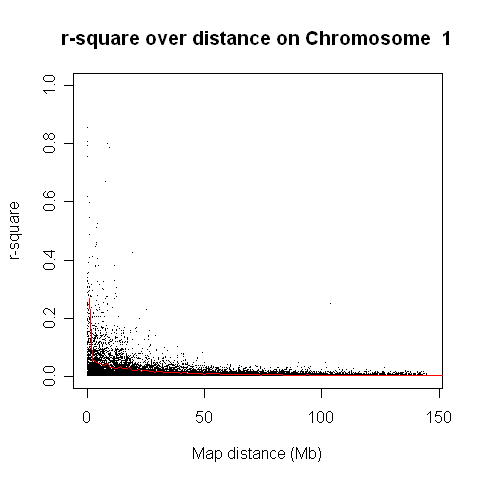
**

**
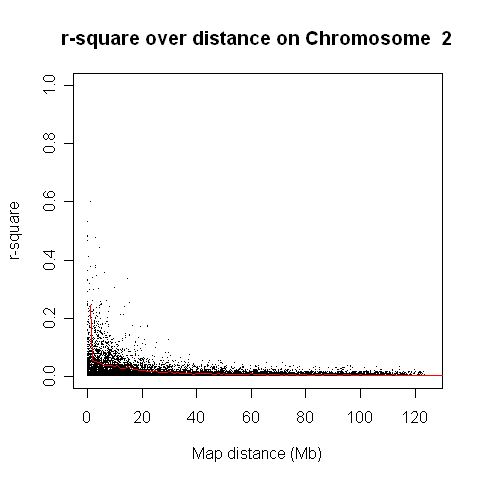

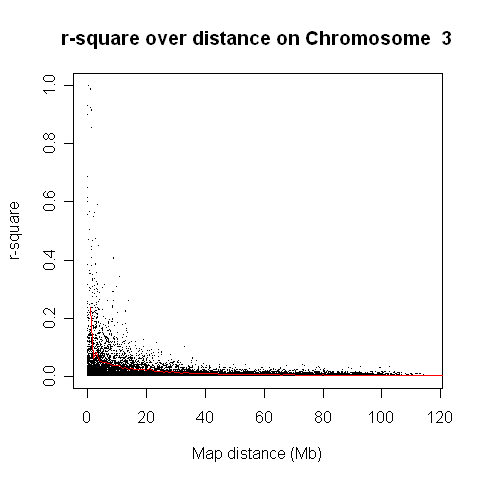

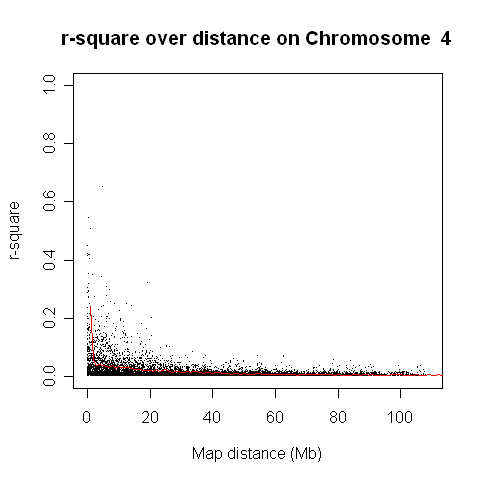

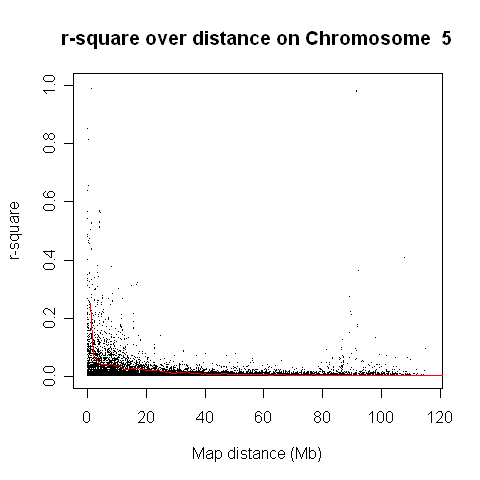

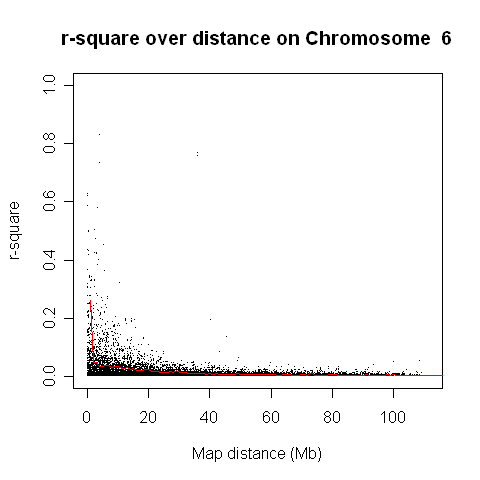

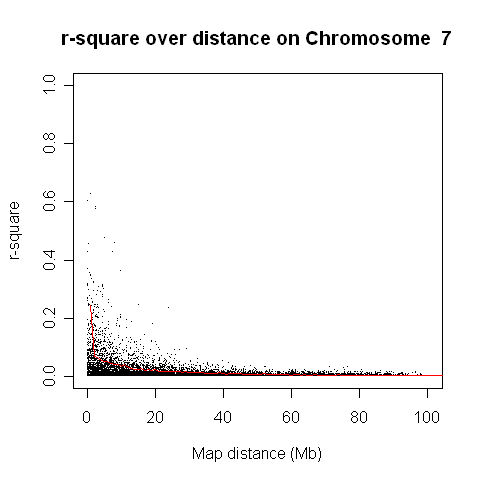

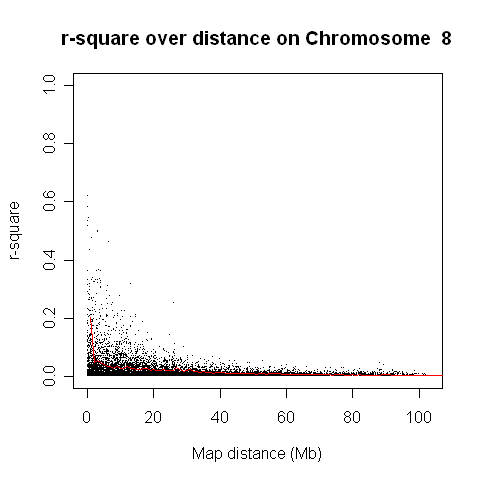

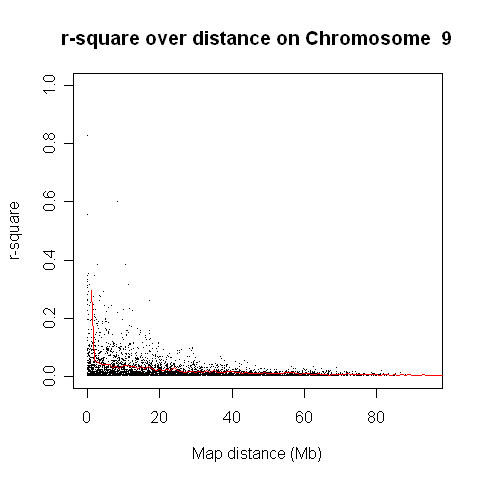

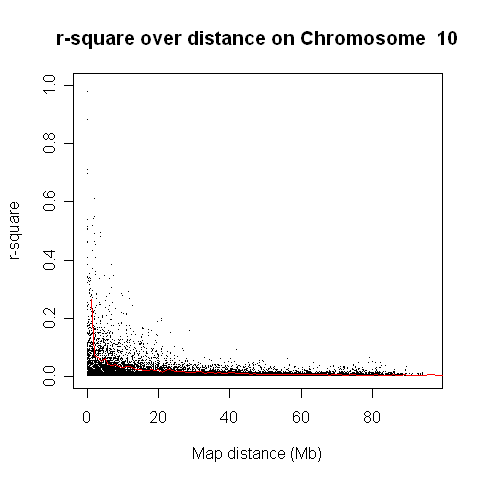

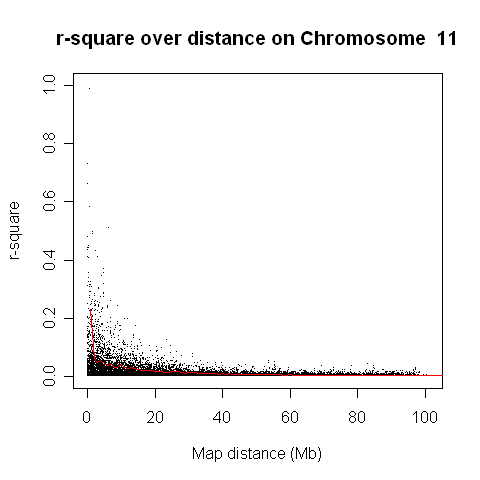

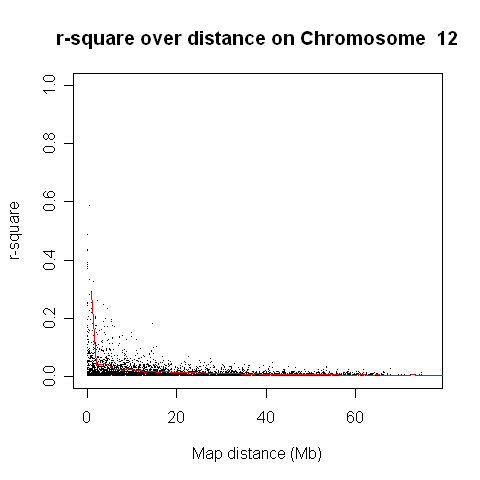

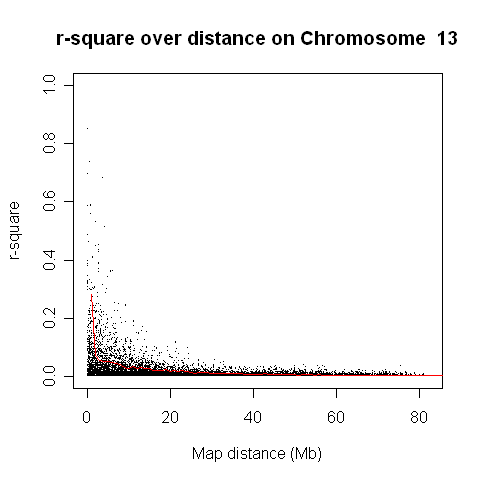

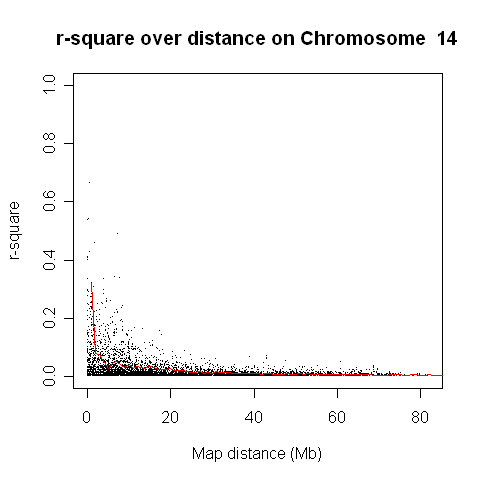

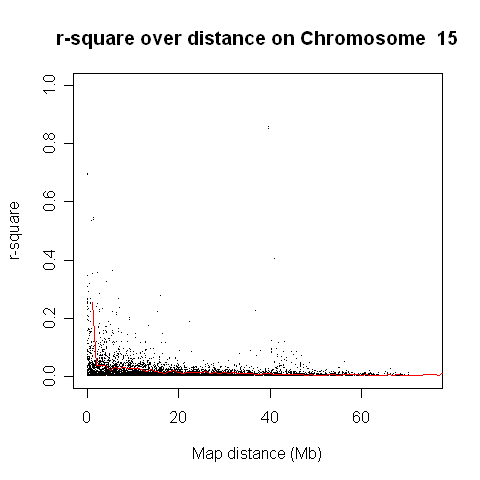

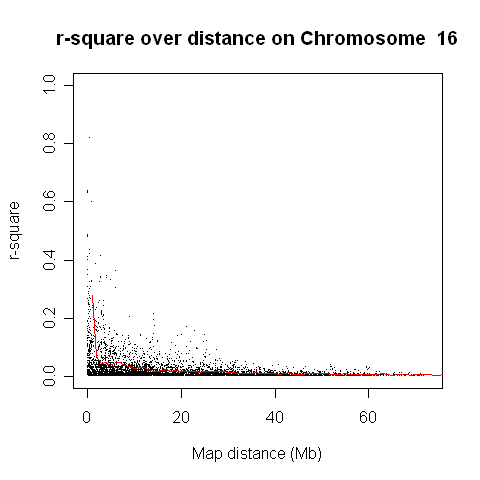

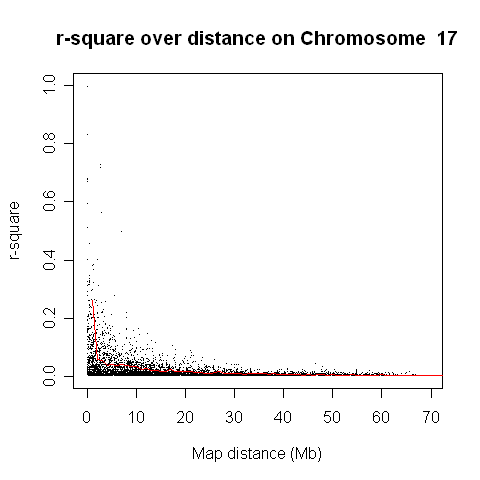

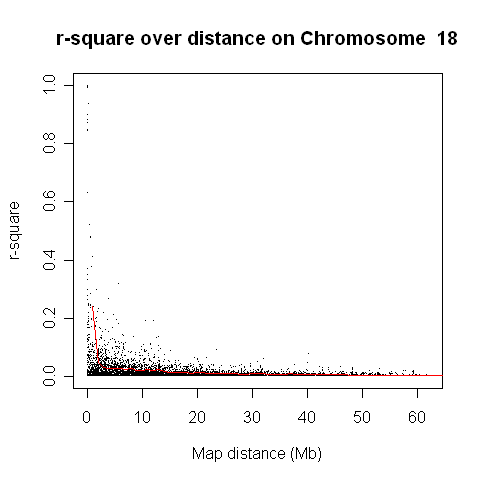

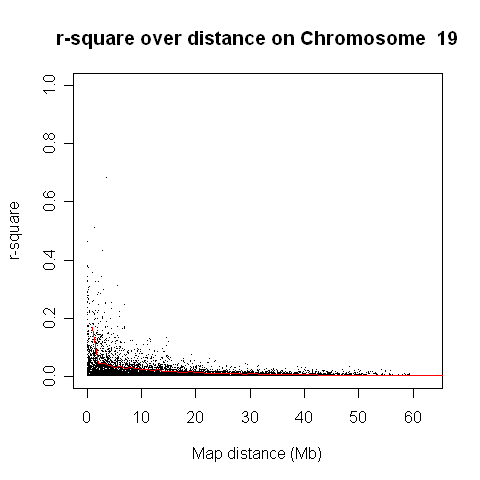

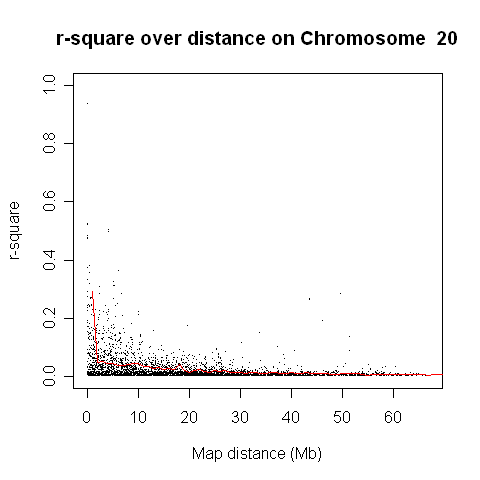

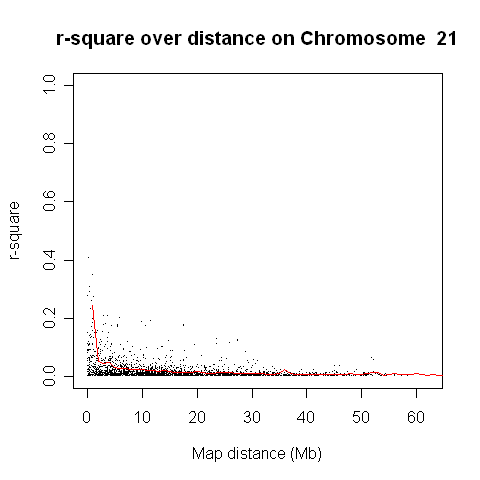

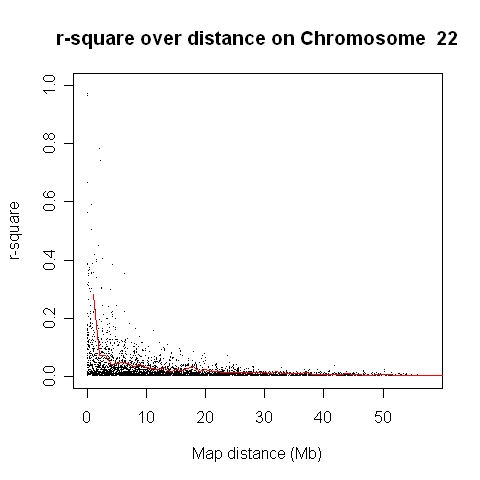

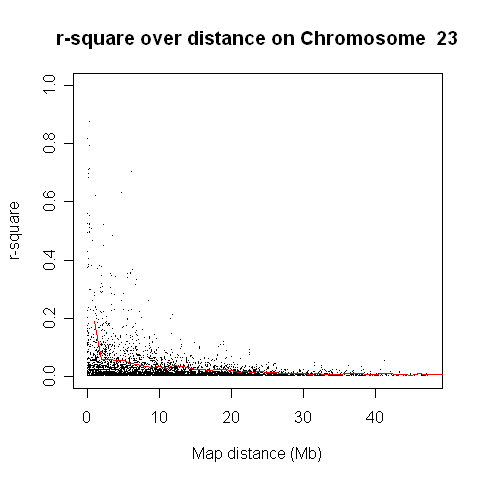

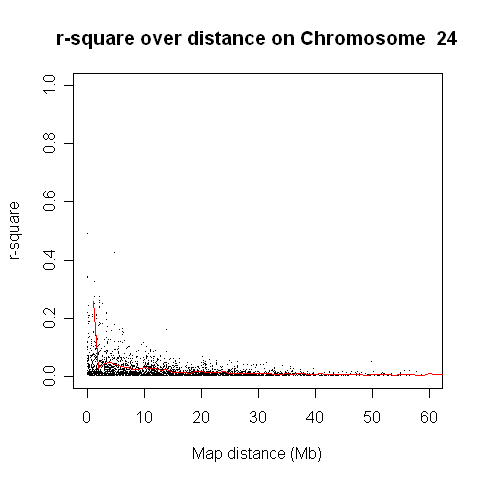

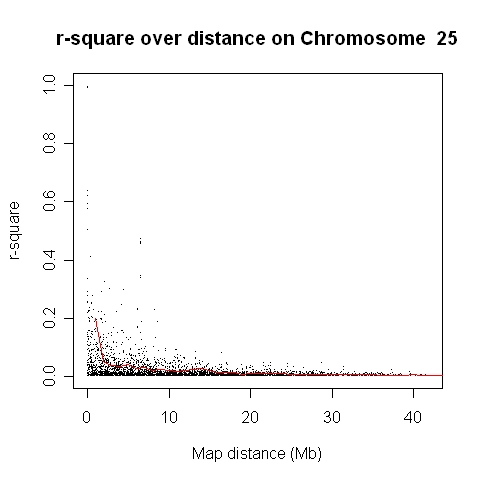

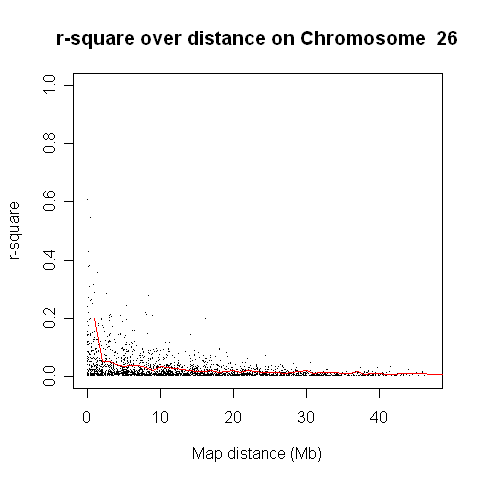

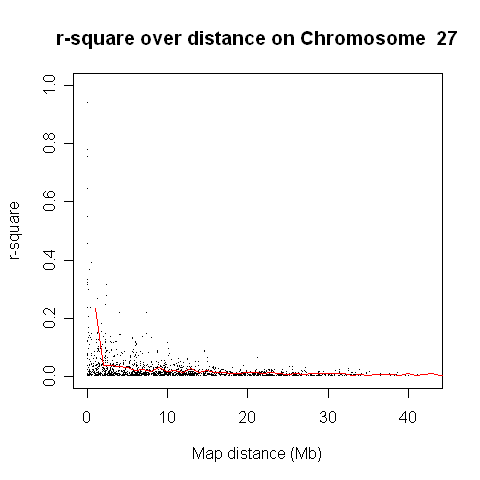

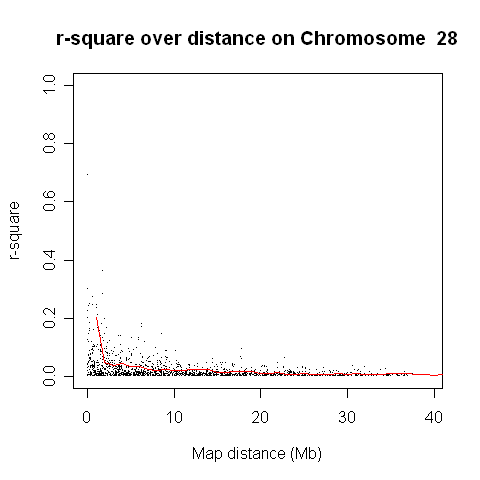

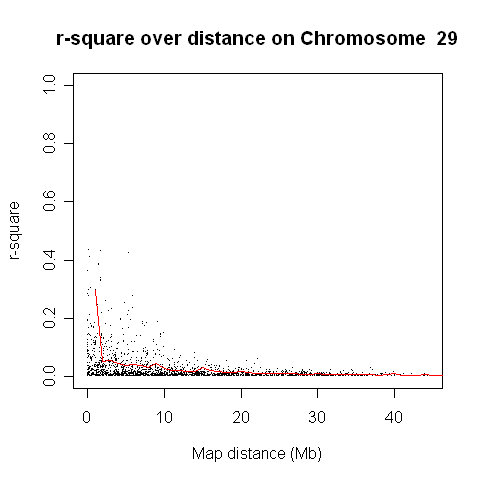
**
