# Supplementary material for: Extent of genome-wide linkage disequilibrium in Australian Holstein-Friesian cattle based on a high-density SNP panel
Source: BMC Genomics. 2008 Apr 24;9:187. doi: 10.1186/1471-2164-9-187 (PMC2386485; doi:10.1186/1471-2164-9-187)

Figure S12. Linkage disequilibrium (*r^2^*) between SNPs on all the bovine autosomal chromosomes (BTA1-BTA29) presented in the form of heatmap of *r^2^*. All SNP with the MAF of less than 0.05 and showing deviation from HWE were excluded in the LD measurement. These figures were prepared by Haploview software. Here black color indicates *r^2^*=1, shades of grey 0< *r^2^* <1 and white *r^2^*=0.

BTA1


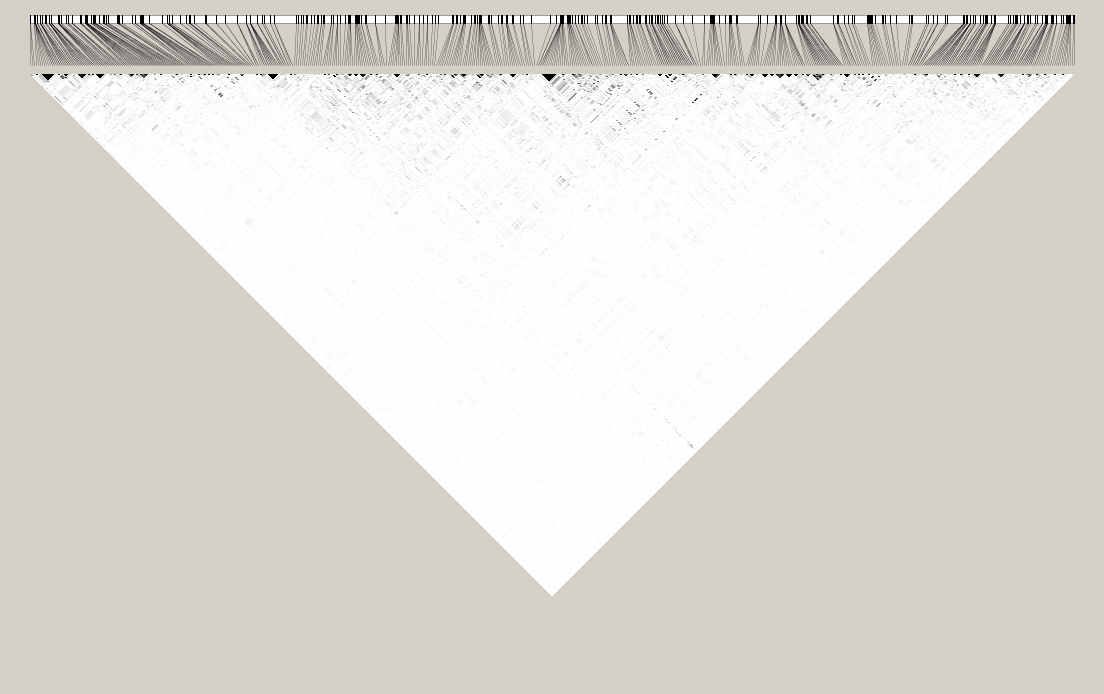


BTA2


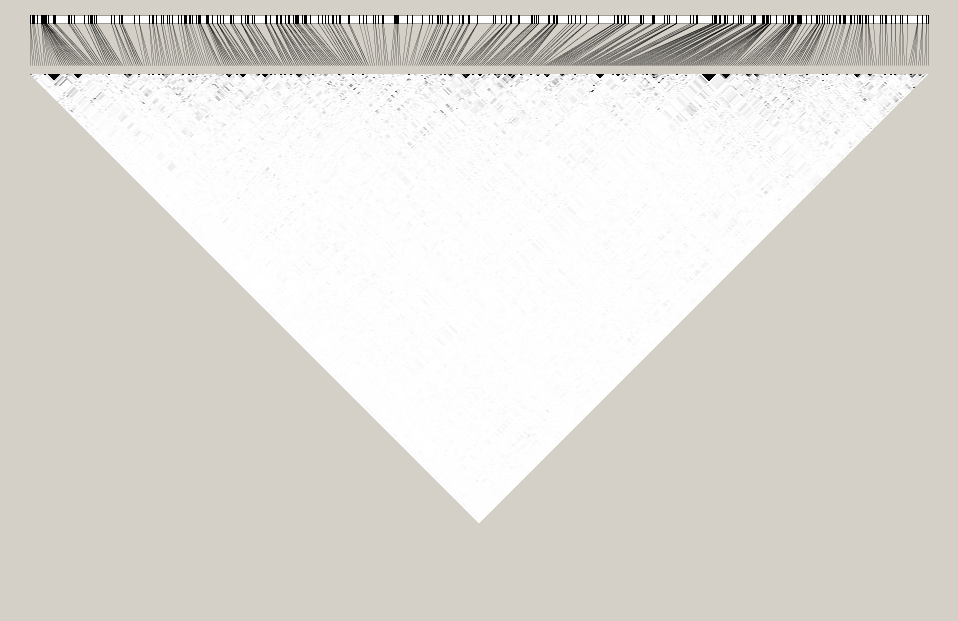


BTA3


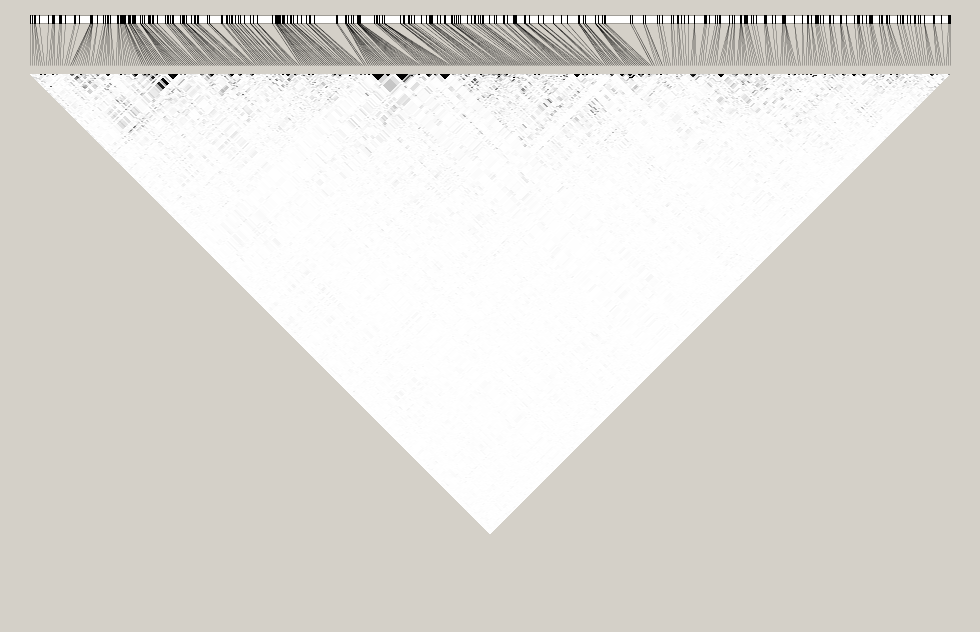


BTA4


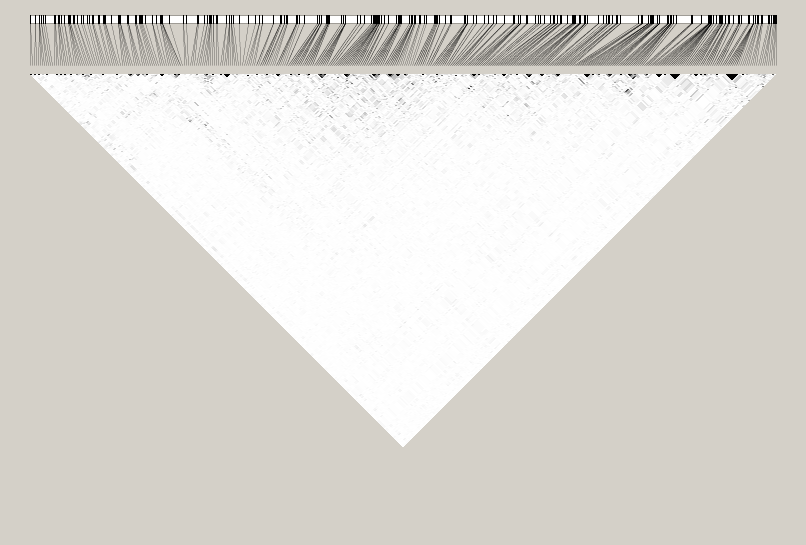


BTA5


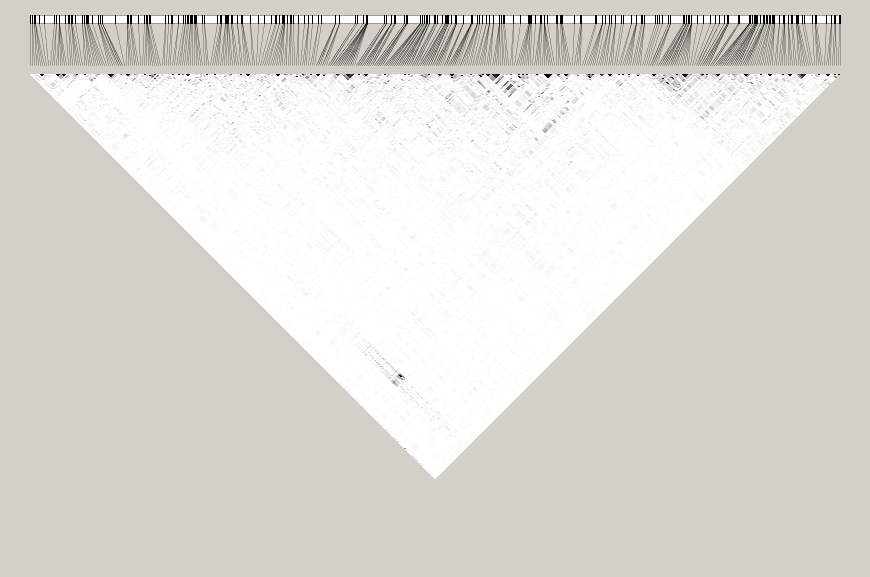


BTA6


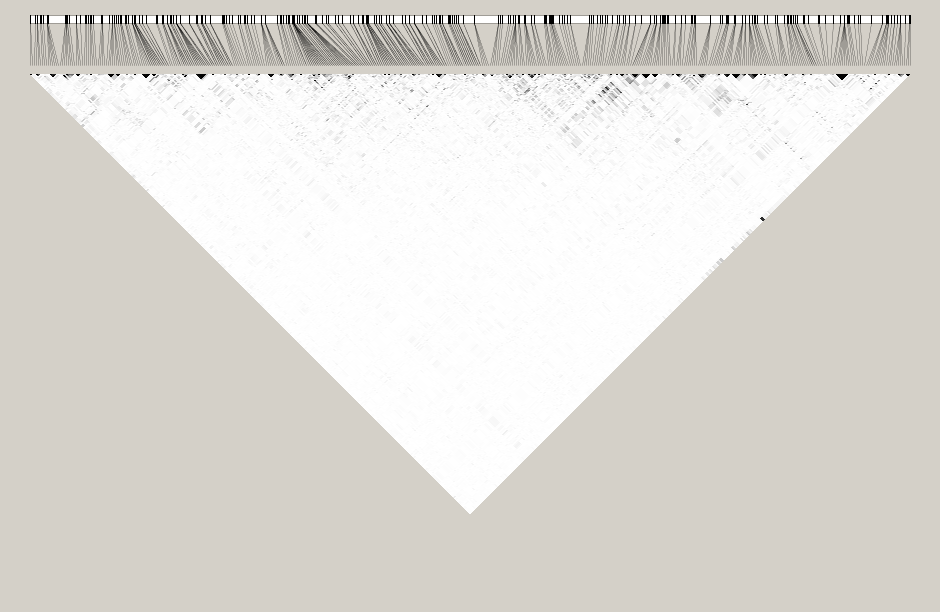


BTA7


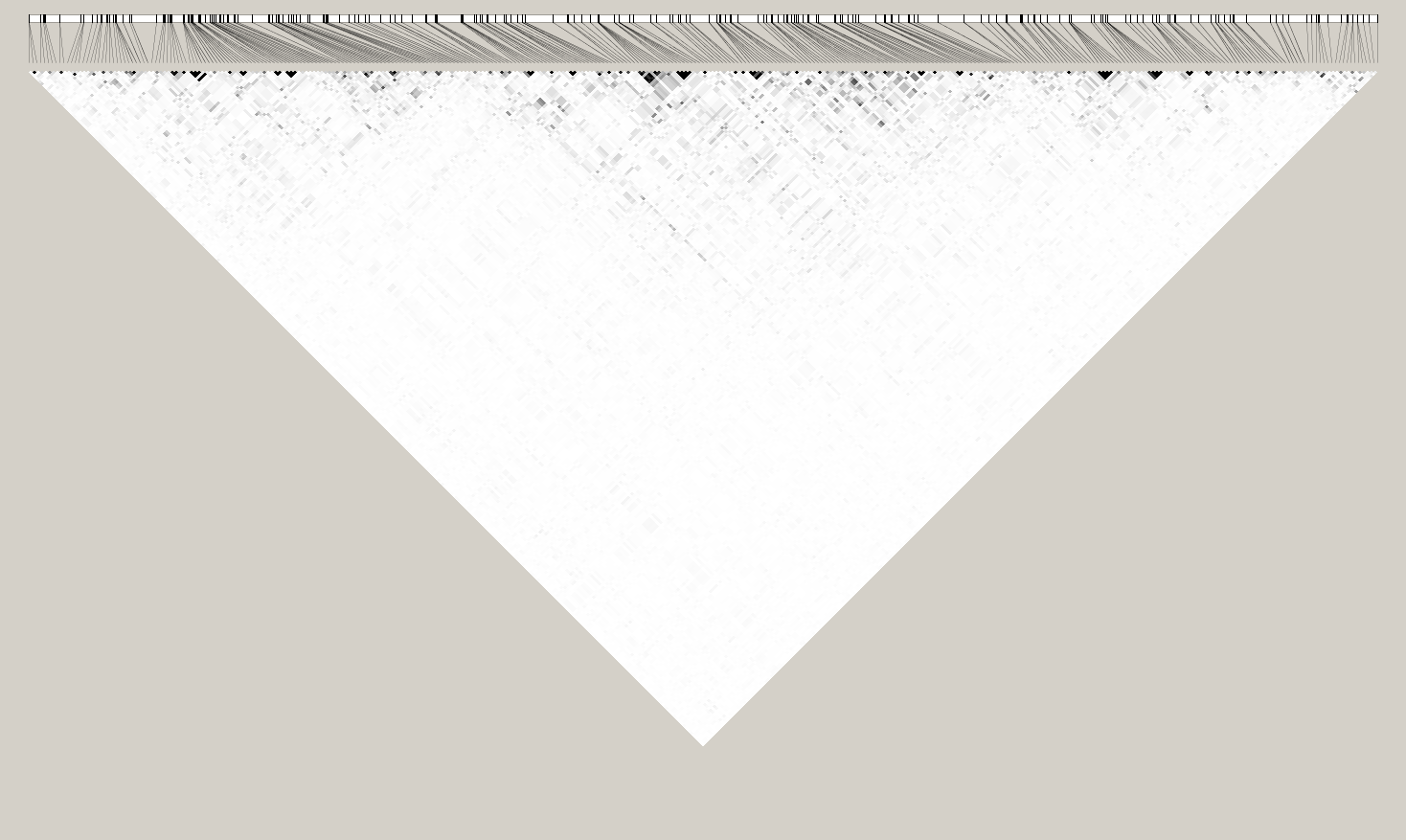


BTA8


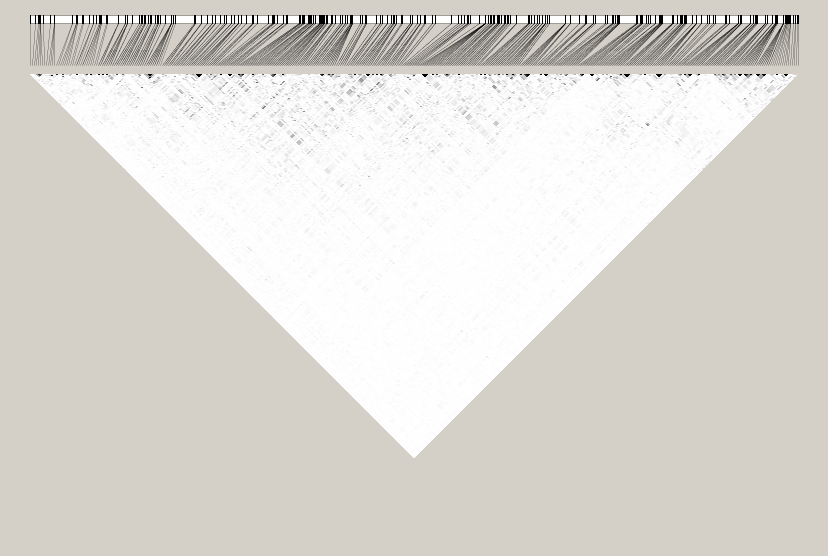


BTA9


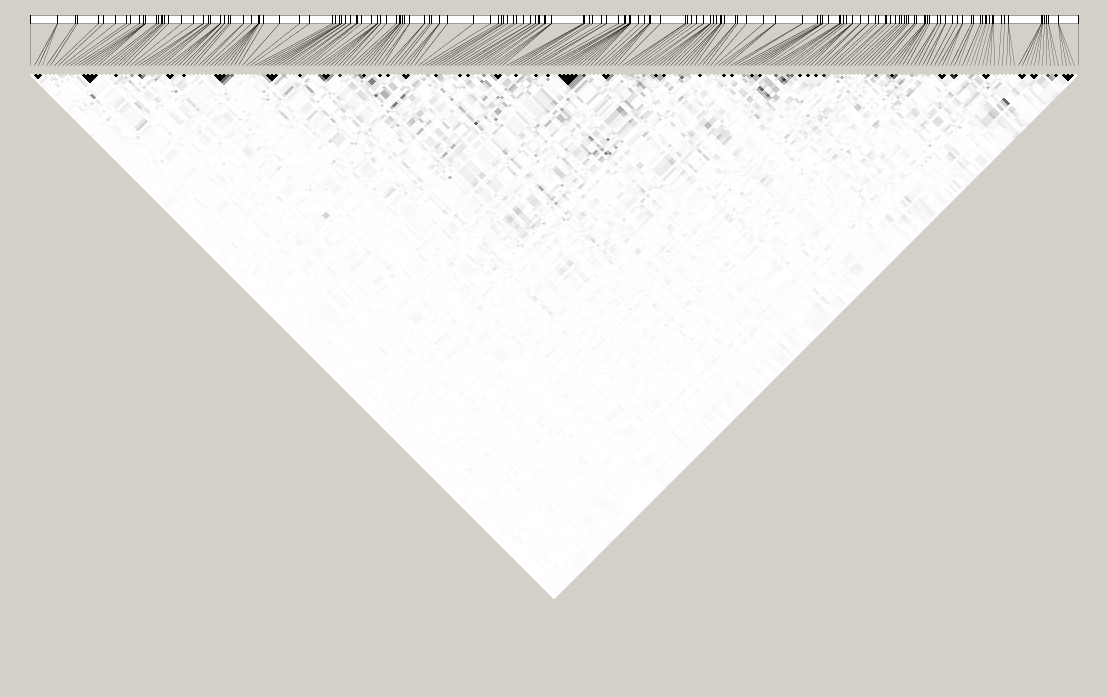


BTA10


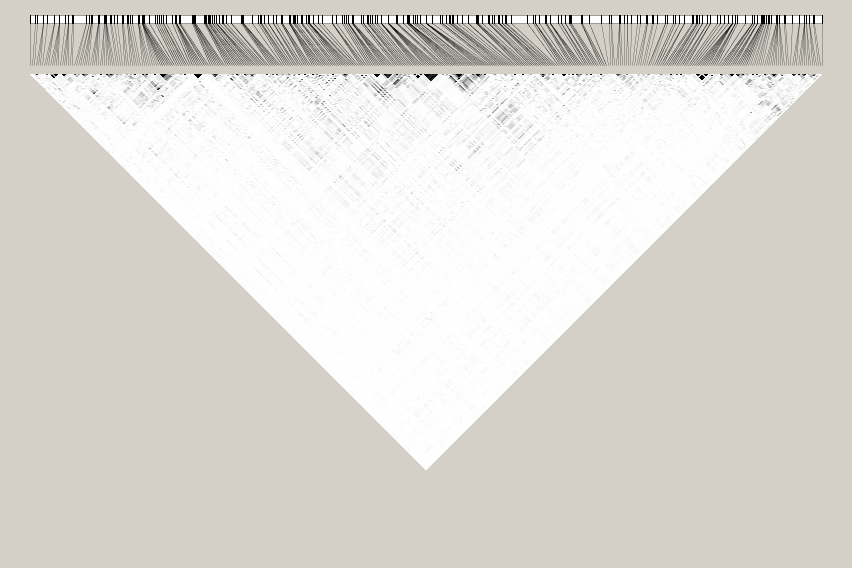


BTA11


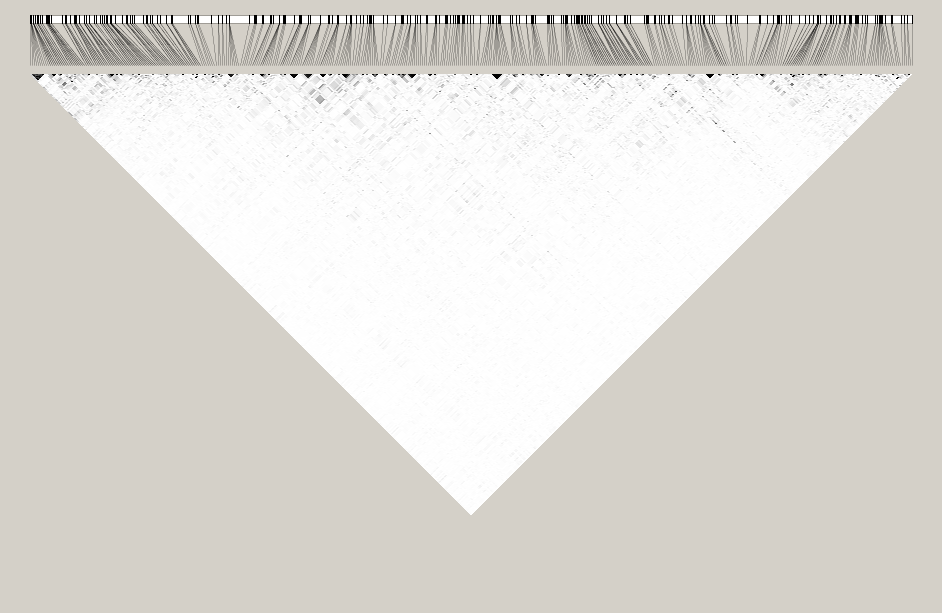


BTA12


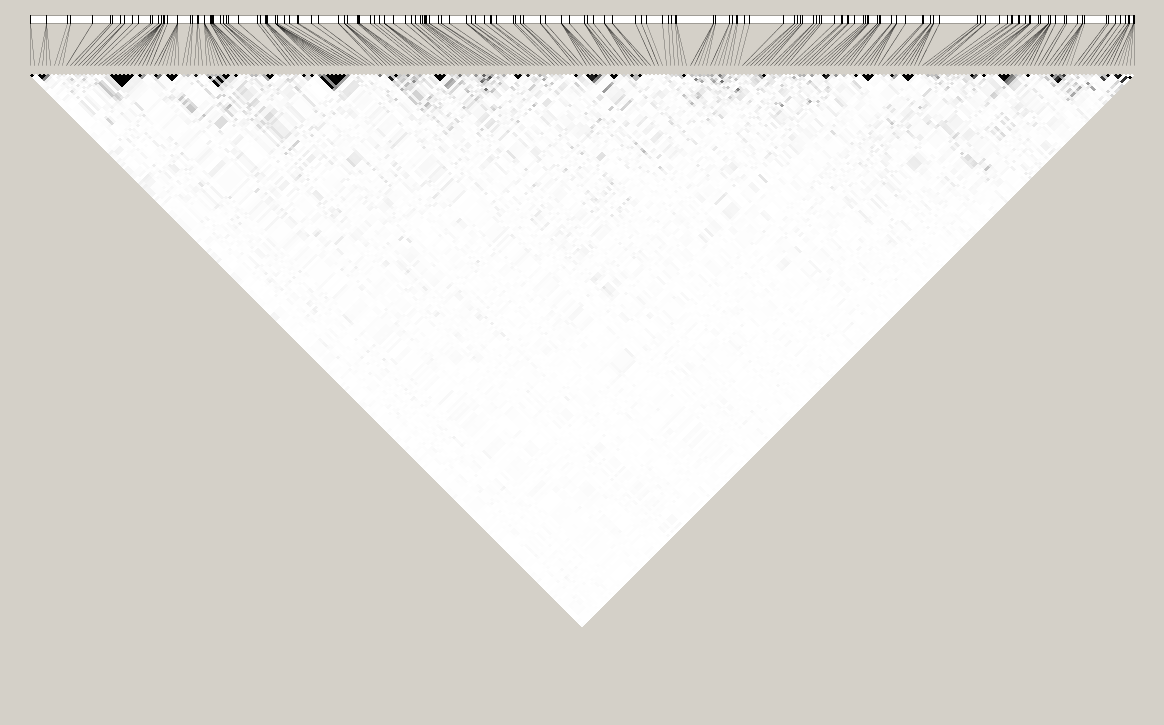


BTA13


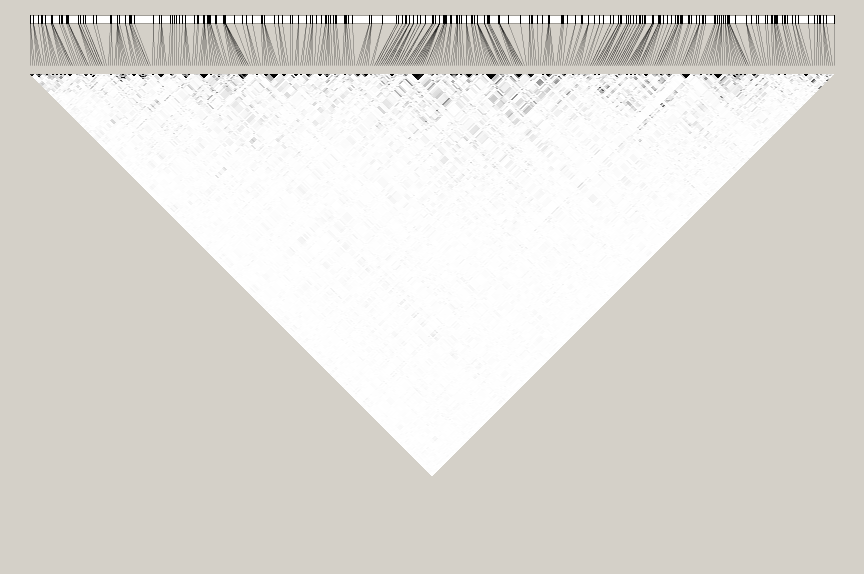


BTA14


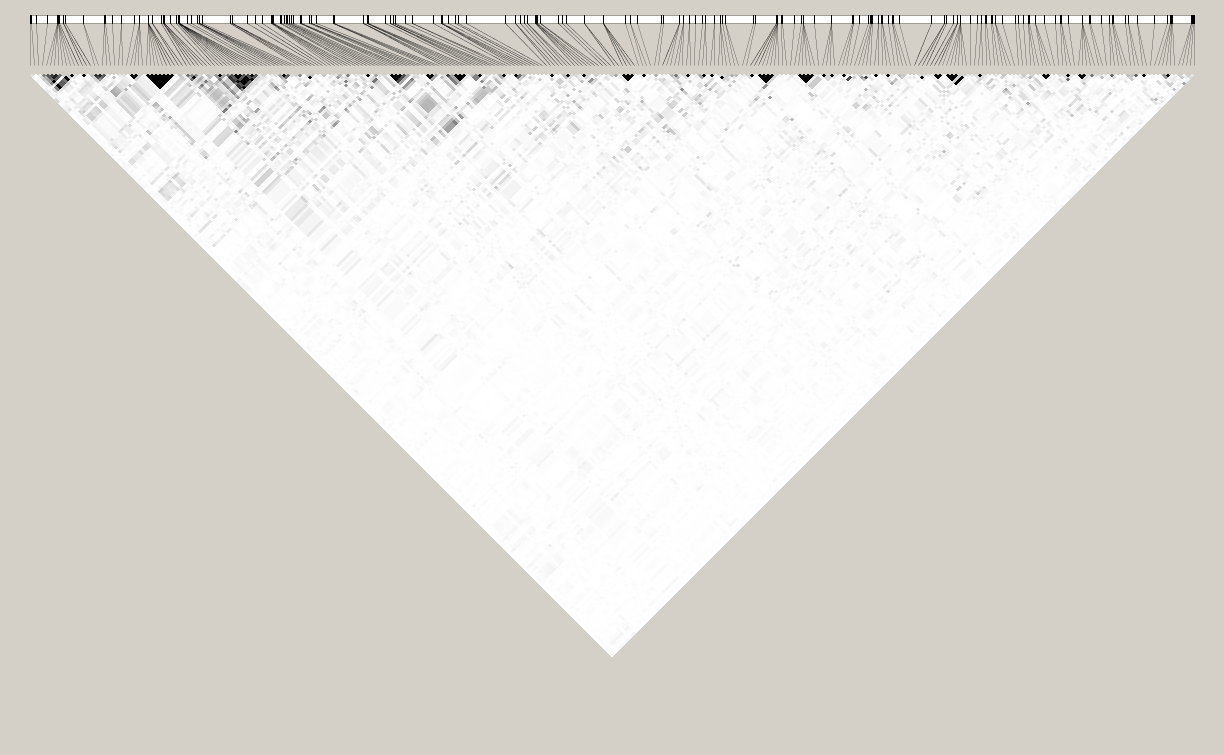


BTA15


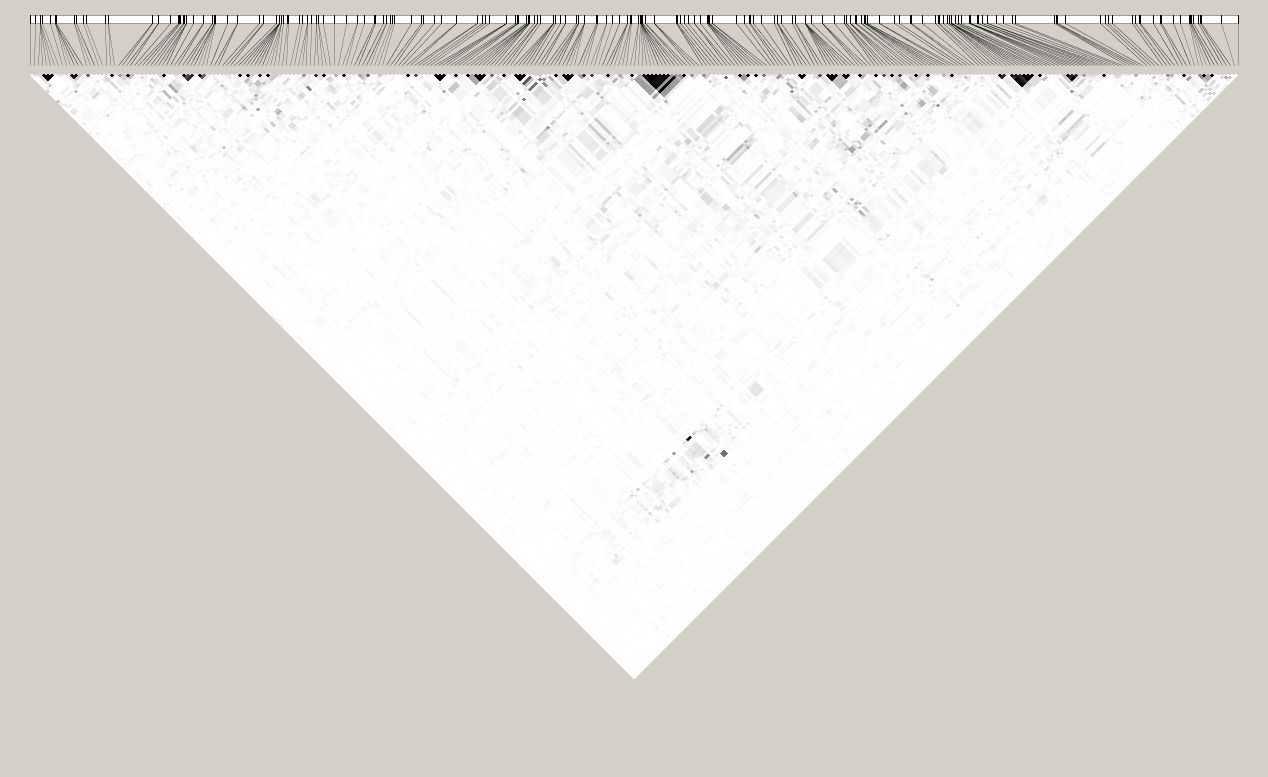


BTA16


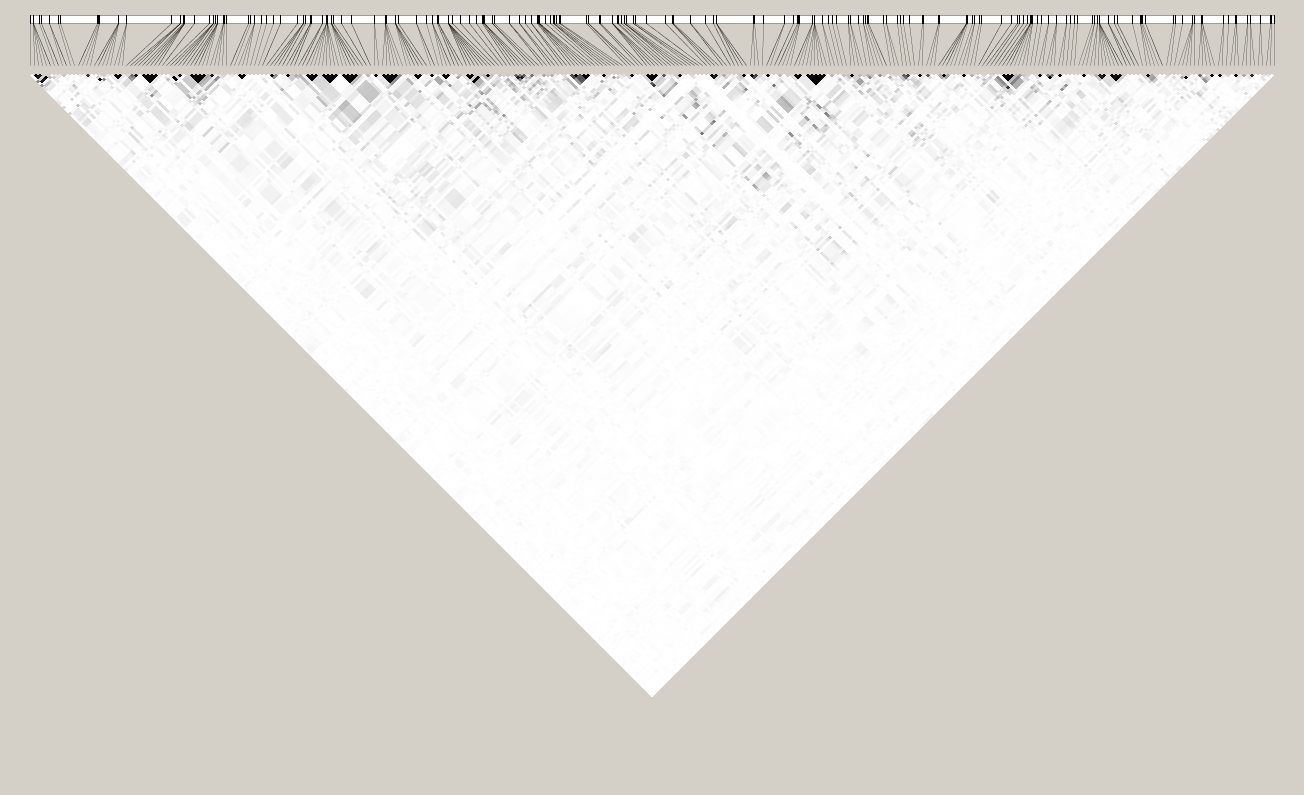


BTA17


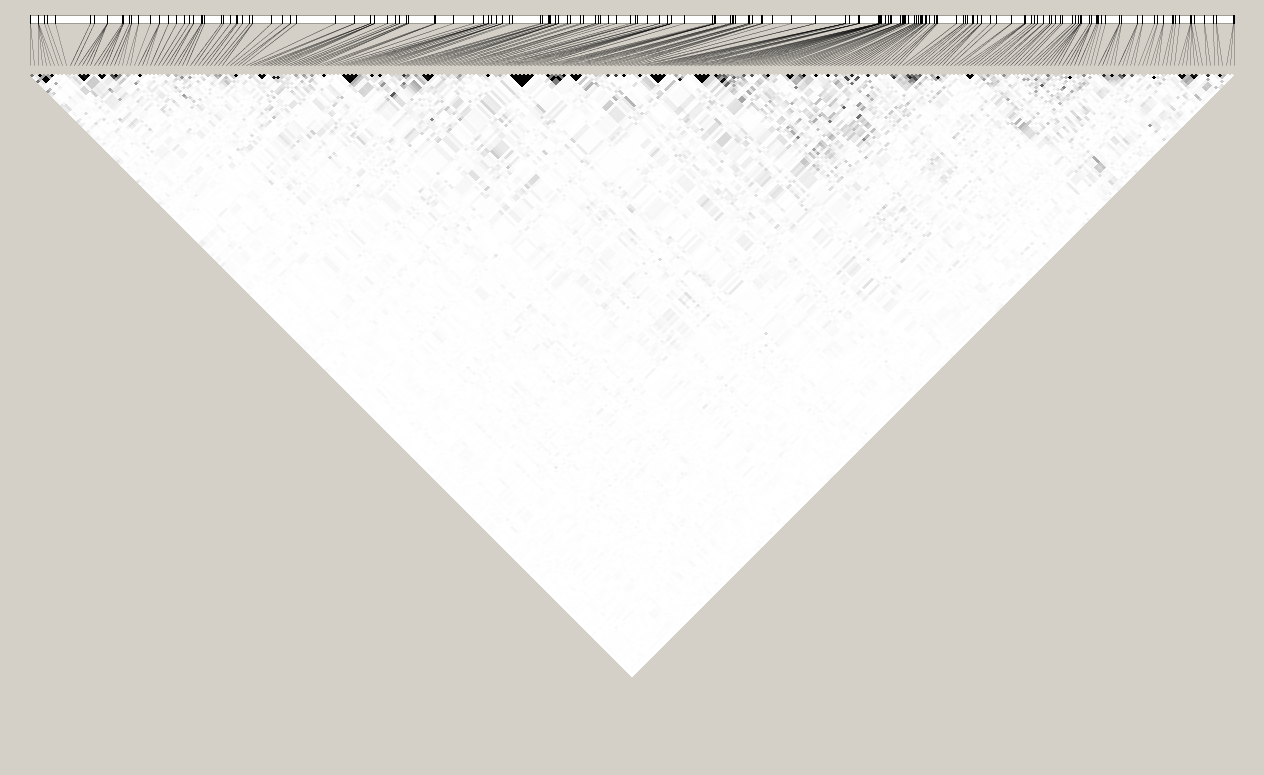


BTA18


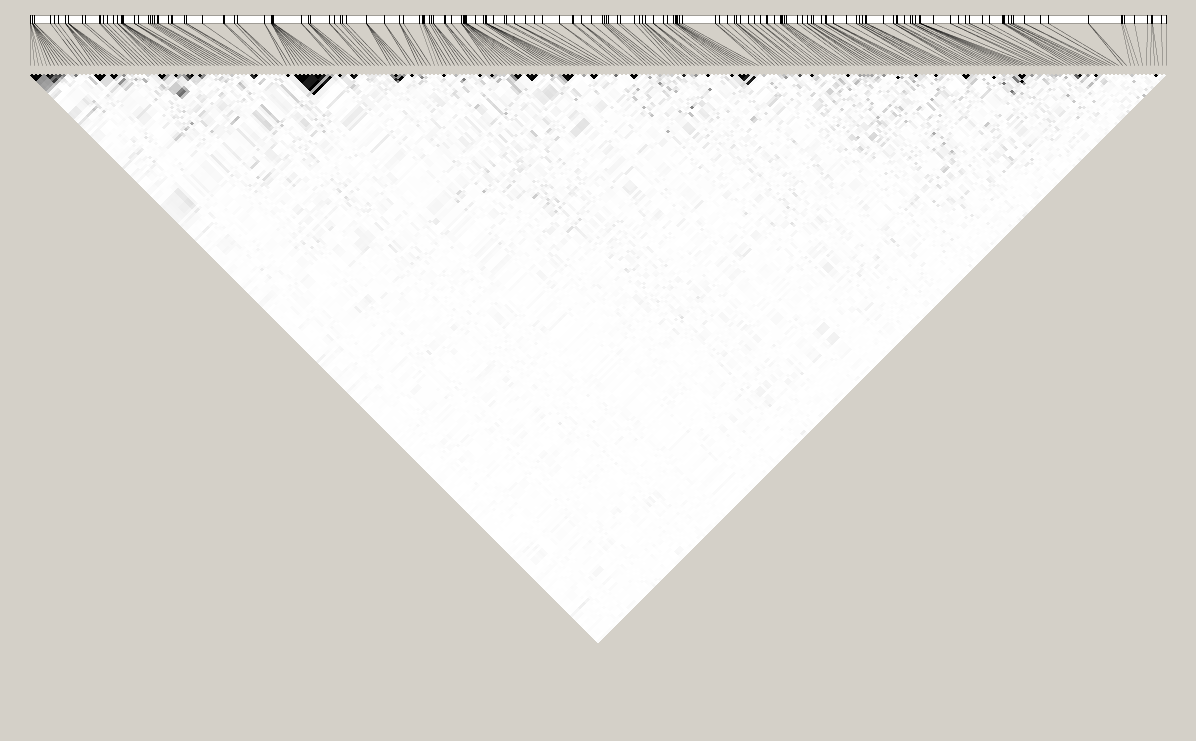


BTA19


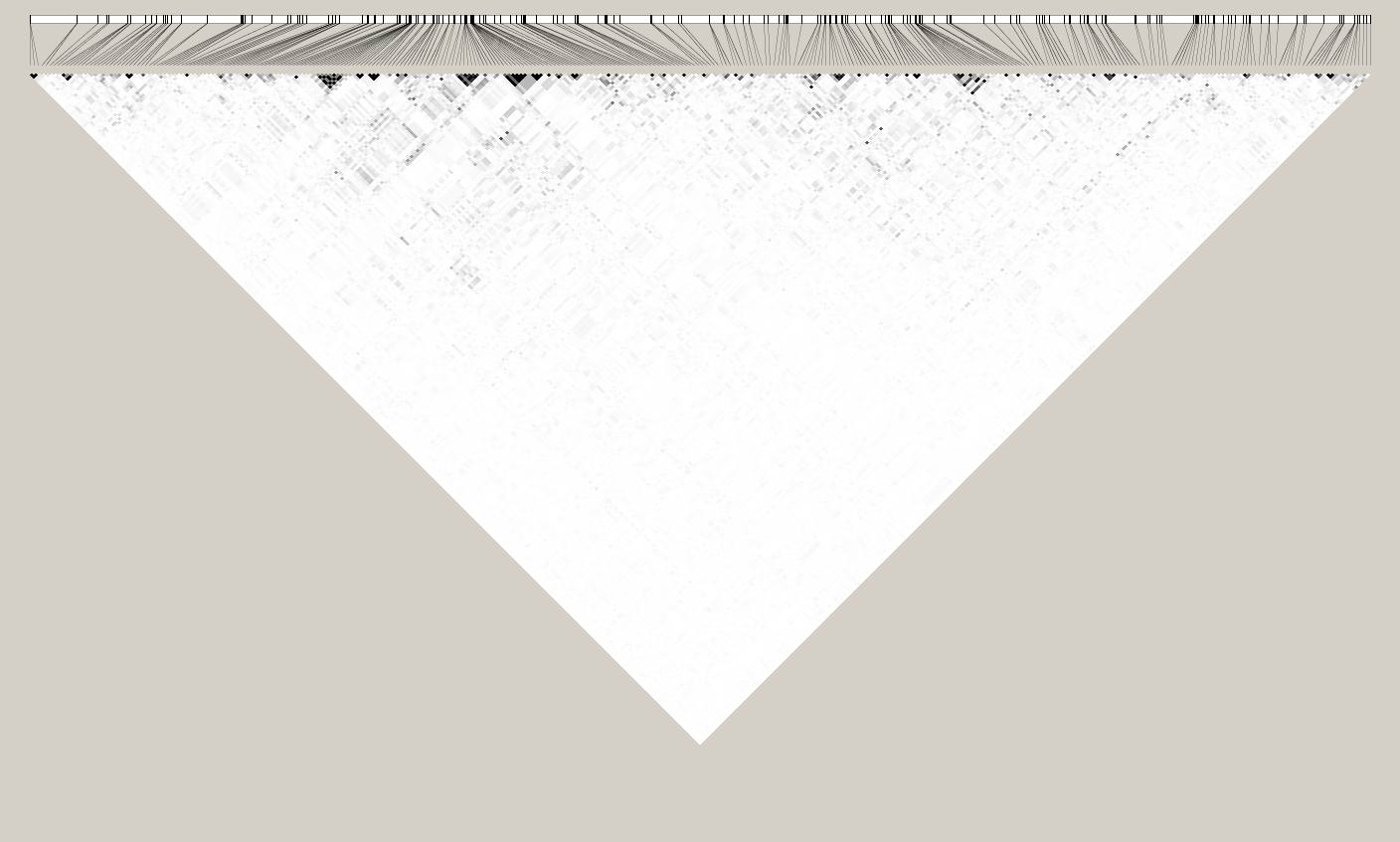


BTA20


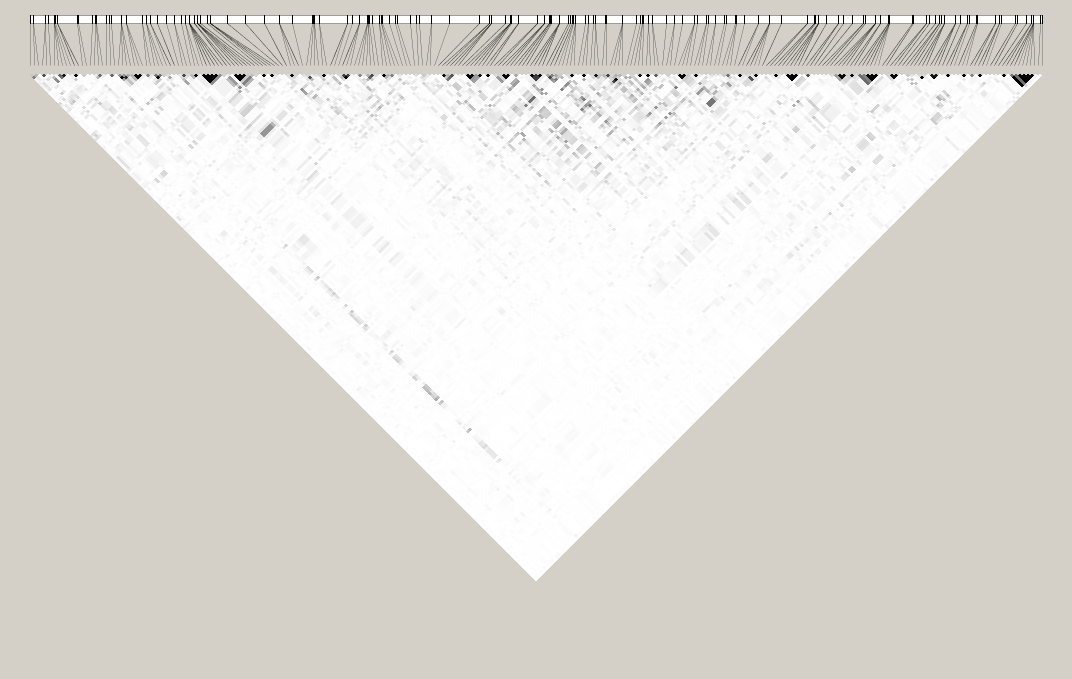


BTA21


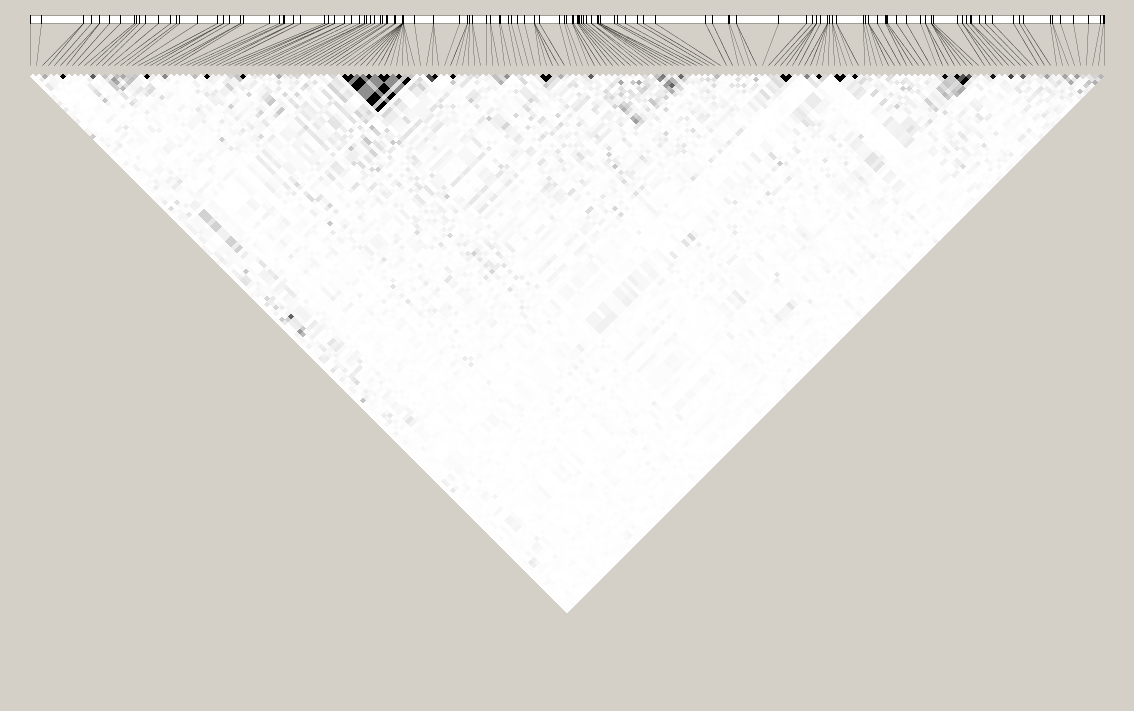


BTA22


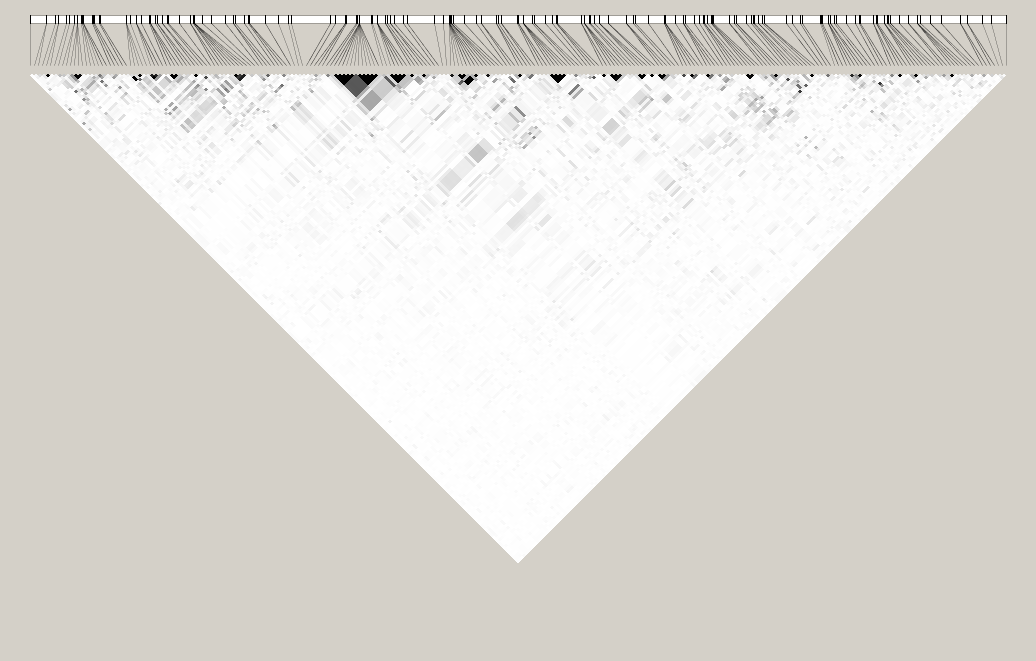


BTA23


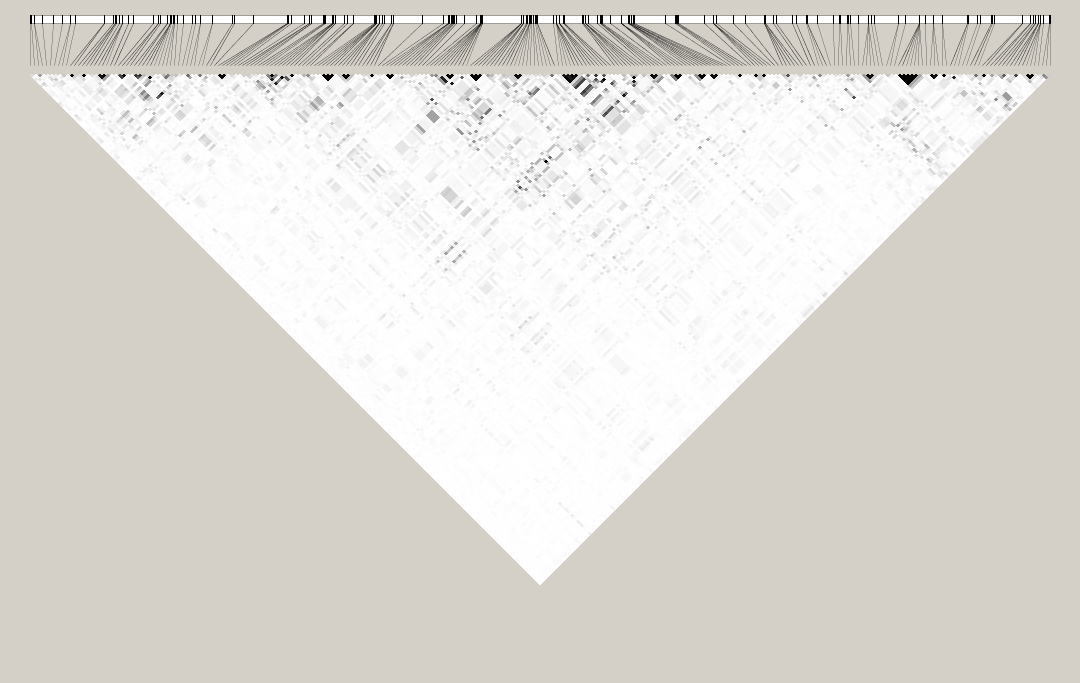


BTA24


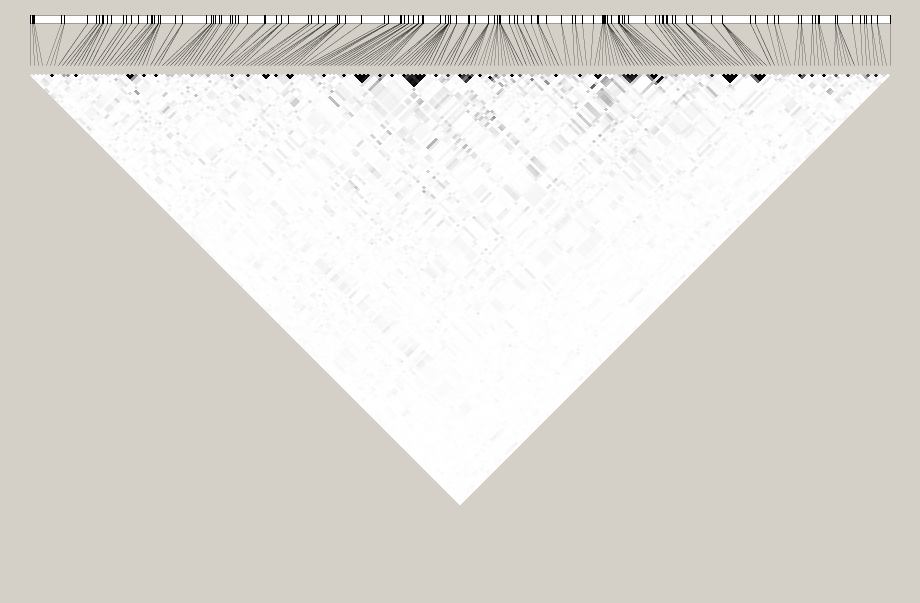


BTA25


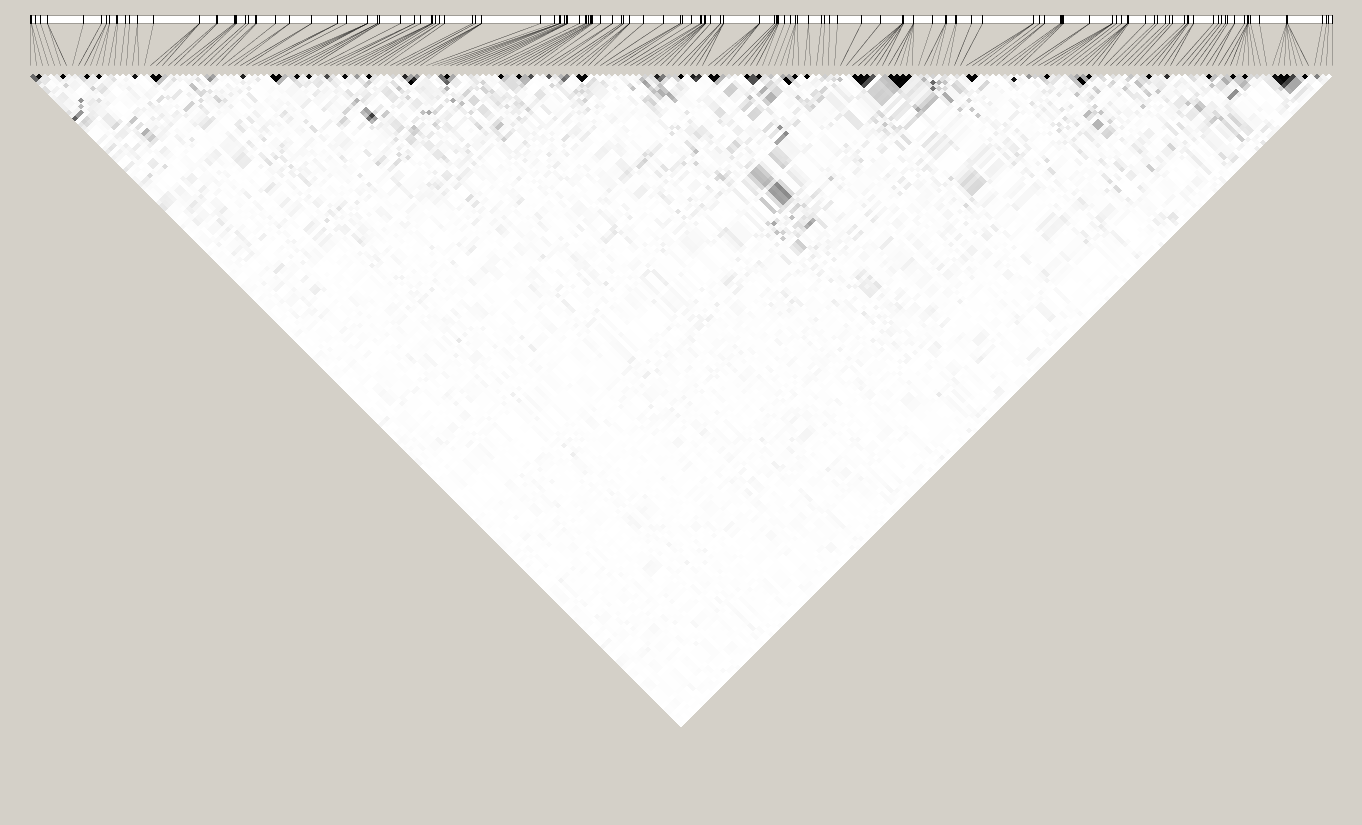


BTA26


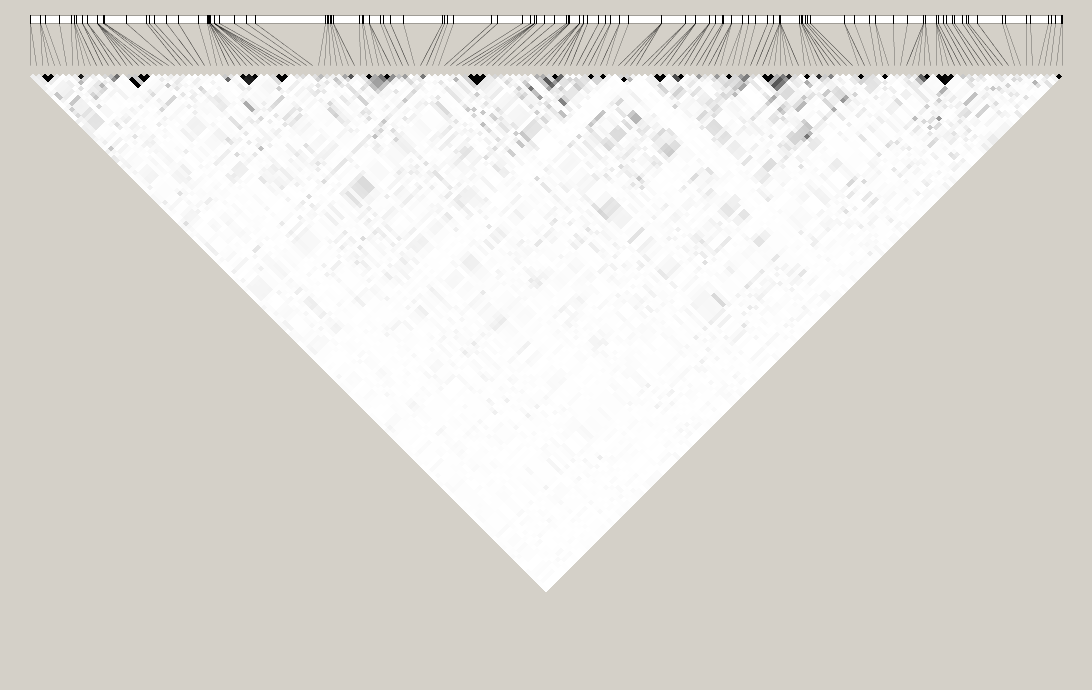


BTA27


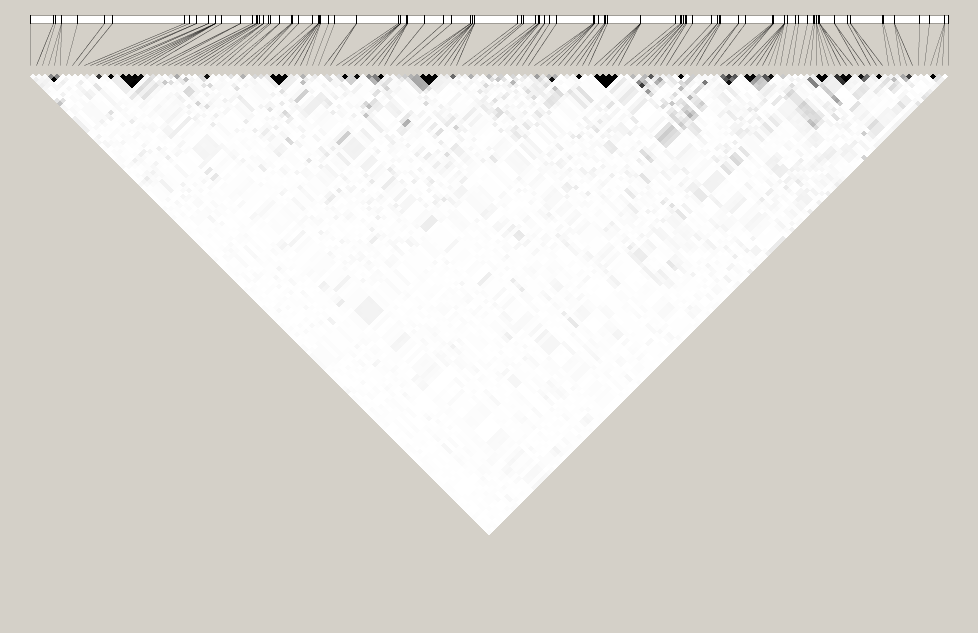


BTA28


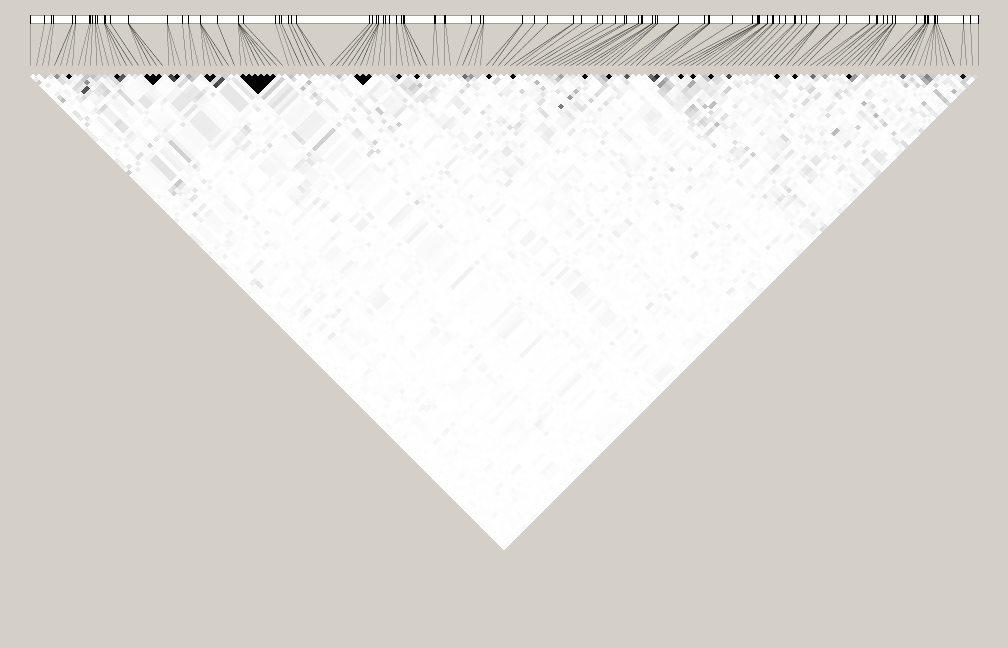


BTA29


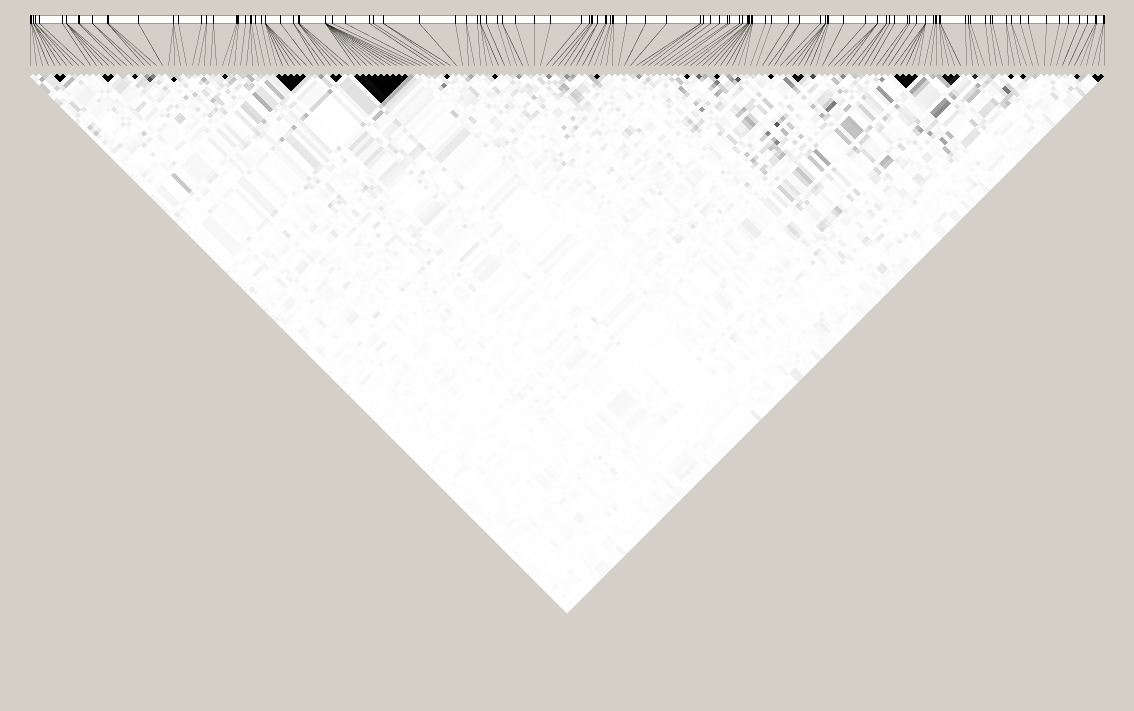

Supplement: Additional file 7 — Figure S12. Linkage disequilibrium (r2) between SNPs on all the bovine autosomal chromosomes (BTA1-BTA29) presented in the form of heatmap of r2 . All SNP with the MAF of less than 0.05 and showing deviation from HWE were excluded in the LD measurement. These figures were prepared by Haploview software. Here black color indicates r2 = 1, shades of grey 0 <r2 < 1 and white r2 = 0. [file 1471-2164-9-187-S7.doc]
