# Supplementary material for: Extent of genome-wide linkage disequilibrium in Australian Holstein-Friesian cattle based on a high-density SNP panel
Source: BMC Genomics. 2008 Apr 24;9:187. doi: 10.1186/1471-2164-9-187 (PMC2386485; doi:10.1186/1471-2164-9-187)
Supplement: Additional file 8 — Table S2. Comparison of pair-wise estimates D' and Dvol for two chromosomes obtained from different sample sizes and compared against a reference sample of 1000 animals. [file 1471-2164-9-187-S8.doc]

Table S2. Comparison of pair-wise estimates *D'* and *Dvol* for two chromosomes obtained from different sample sizes and compared against a reference sample of 1000 individuals.

| **Sample 1** | **Sample 2** | **chromosome** | *Correlation between the estimates from two different samples* | | | | |
| --- | --- | --- | --- | --- | --- | --- | --- |
|  |  |  | ***D' and D_sample_*** | ***Dvol and Dvol_sample_*** | **D_sample_ and Dvol_sample_** | ***D'* and Dvol_sample_** | **Number of common pairs** |
| 1000 | 75 | 28 | 0.559 | **0.548** | 0.927 | 0.548 | 12561 |
| 1000 | 75 | 29 | 0.615 | **0.595** | 0.927 | 0.596 | 15051 |
| 1000 | 100 | 28 | 0.646 | **0.635** | 0.963 | 0.636 | 12561 |
| 1000 | 100 | 29 | 0.695 | **0.686** | 0.963 | 0.686 | 15576 |
| 1000 | 200 | 28 | 0.802 | **0.796** | 0.985 | 0.795 | 12561 |
| 1000 | 200 | 29 | 0.802 | **0.795** | 0.987 | 0.794 | 15576 |
| 1000 | 300 | 28 | 0.885 | **0.882** | 0.995 | 0.882 | 12561 |
| 1000 | 300 | 29 | 0.883 | **0.881** | 0.994 | 0.880 | 15931 |
| 1000 | 400 | 28 | 0.906 | **0.905** | 0.997 | 0.905 | 12561 |
| 1000 | 400 | 29 | 0.918 | **0.919** | 0.996 | 0.918 | 15931 |
| 1000 | 500 | 28 | 0.933 | **0.932** | 0.997 | 0.932 | 12561 |
| 1000 | 500 | 29 | 0.940 | **0.939** | 0.996 | 0.938 | 16110 |

***D' and D_sample_*** are *D'* estimates based on 1000 and sample 2, respectively.

***Dvol and Dvol_sample_*** are *Dvol* estimates based on 1000 and sample 2, respectively.
